# Supplementary material for: Co-delivery of lentiviral vectors and Cas9-containing virus-like particles enables rapid, scalable manufacture of gene-edited CAR T cells
Source: Mol Ther Adv. 2026 Jul 9;34(3):201809. doi: 10.1016/j.omta.2026.201809 (PMC13446315; doi:10.1016/j.omta.2026.201809)
Supplement: Supplementary file 1 [file mmc1.pdf]

OMTA, Volume *34*

Supplemental information

Co-delivery of lentiviral vectors and Cas9-containing virus-like particles enables rapid, scalable manufacture of gene-edited CAR T cells

Francesca Ferrara, Matthew M. Wielgosz, Jeoungeun J. Park, Matthew Bauler, Chris Wincek, Nataly Mier, Yin Su, Li Tang, Brandon R. Lowe, Jordan A. Beard, Deanna M. Langfitt, Sheng Zhou, and Robert E. Throm

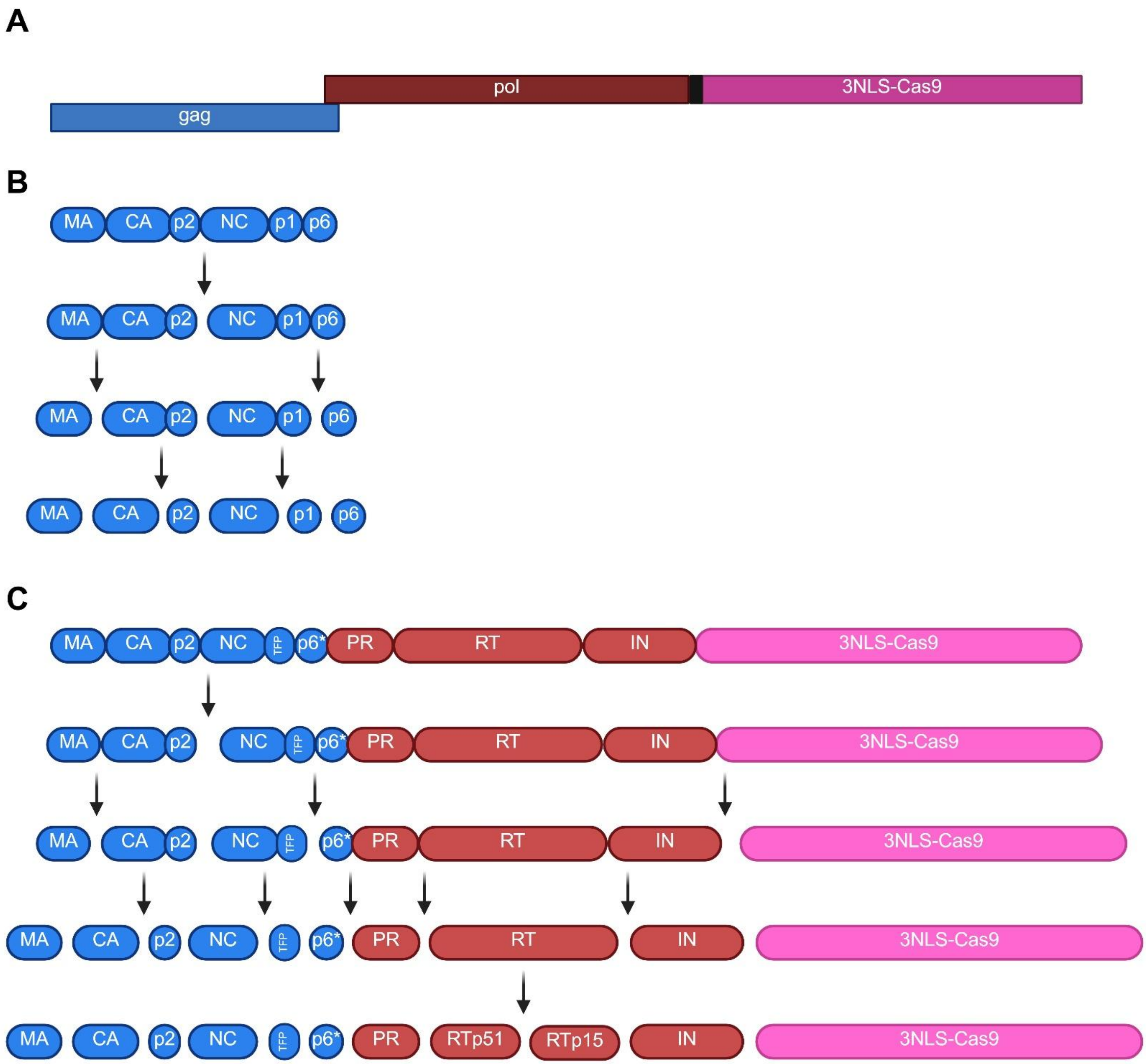

Figure S1: Expected translation and processing of the HIV-1 Gag-Pol-Cas9 fusion polyprotein.
Cas9 sequence was fused to the C-terminal domain of the Gag-Pol sequence using the HIV-1 matrix-capsid cleavage (QVSQNY↑PIVQNI) linker peptide (A). Two different polyproteins are translated from a common transcript and processed by the HIV-1 protease during particle maturation: a Gag polyprotein (B) and a Gag-Pol-Cas9 polyprotein (C).

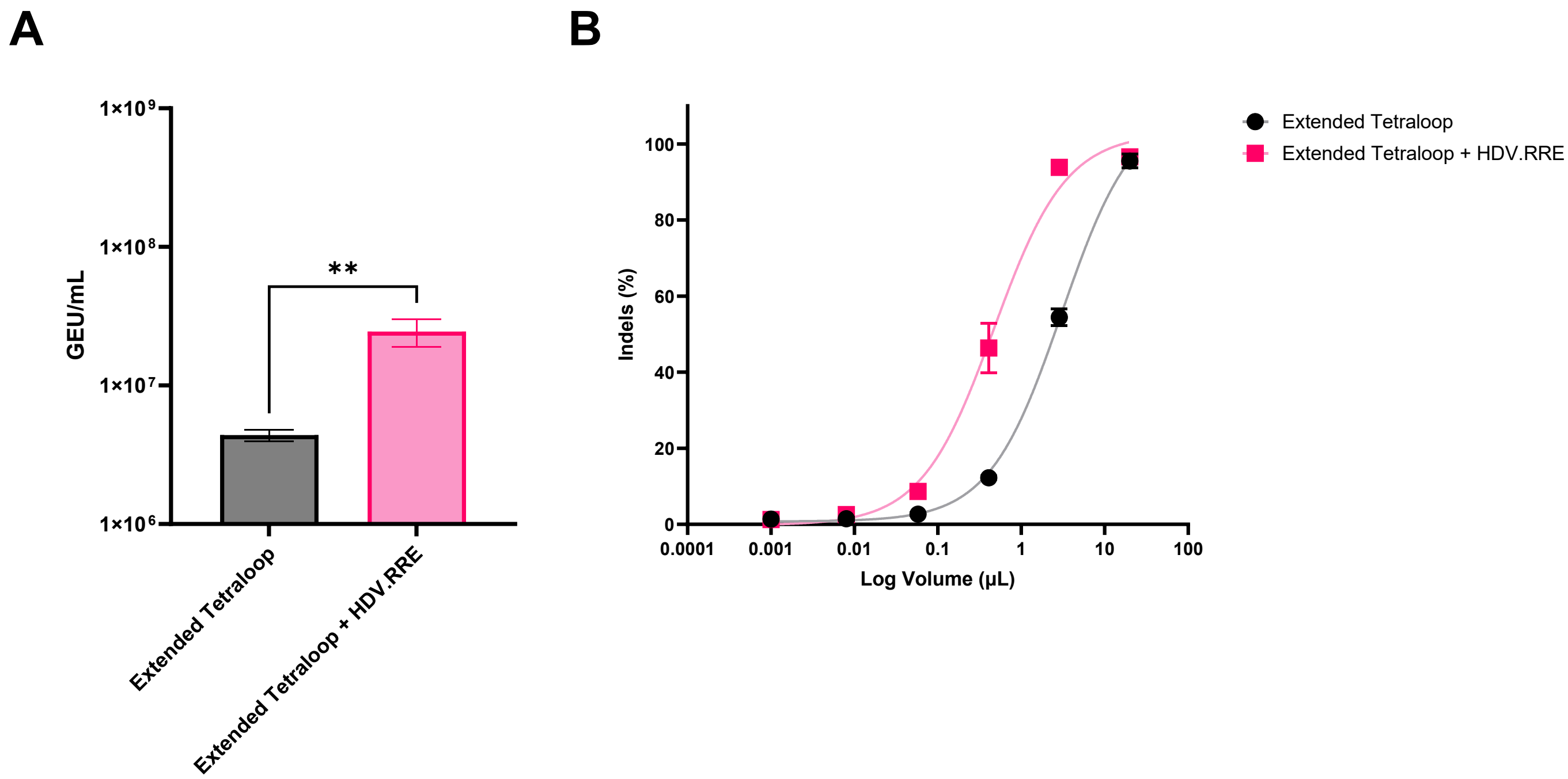

Figure S2: Effect of the Hepatitis D virus ribozyme and Rev Response Element in sgRNA scaffold for Cas9 VLP production

VLPs were produced using a plasmid expressing B2M sgRNA with a modified scaffold (extended tetraloop stem and without a premature termination sequence) with and without the HDV ribozyme and RRE sequences at its 3. Editing activity was evaluated in transduction of HOS-mCat1 cells and is reported as gene editing units per mL (GEU/mL) (A) and as percentage of indels per µL of VLP tested (B). n=3.

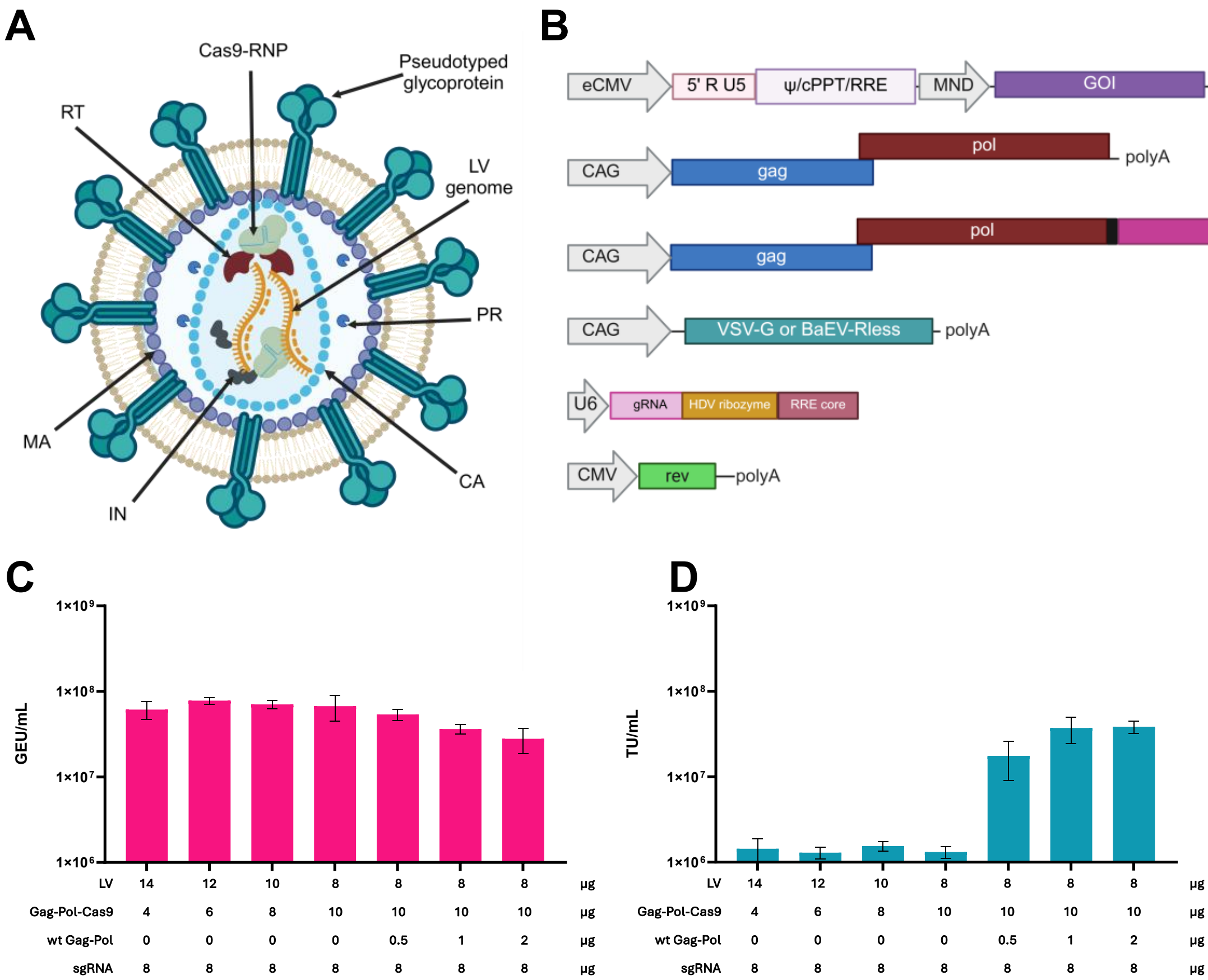

Figure S3: Production and activity of Lentiviral Vector Cas9 (LVC)
Schematic representation of the predicted structure of LVC based on HIV-1 structure (A). Design of plasmids used to produce LVC: a LV vector transfer plasmid; a structural plasmid expressing HIV-1 Gag-Pol; a structural plasmid expressing HIV-1 Gag-Pol fused with 3NLS-Cas9; a plasmid expressing an envelope protein (VSV-G); a plasmid expressing an sgRNA; and a plasmid encoding HIV-1 Rev (B). Evaluation of LVC gene editing activity on HOS-mCat1 cells produced using various plasmid ratios (C-D). Editing activity is reported as gene editing units per mL (GEU/mL), and transduction activity is reported as transduction units per mL(TU/mL). n = 3.

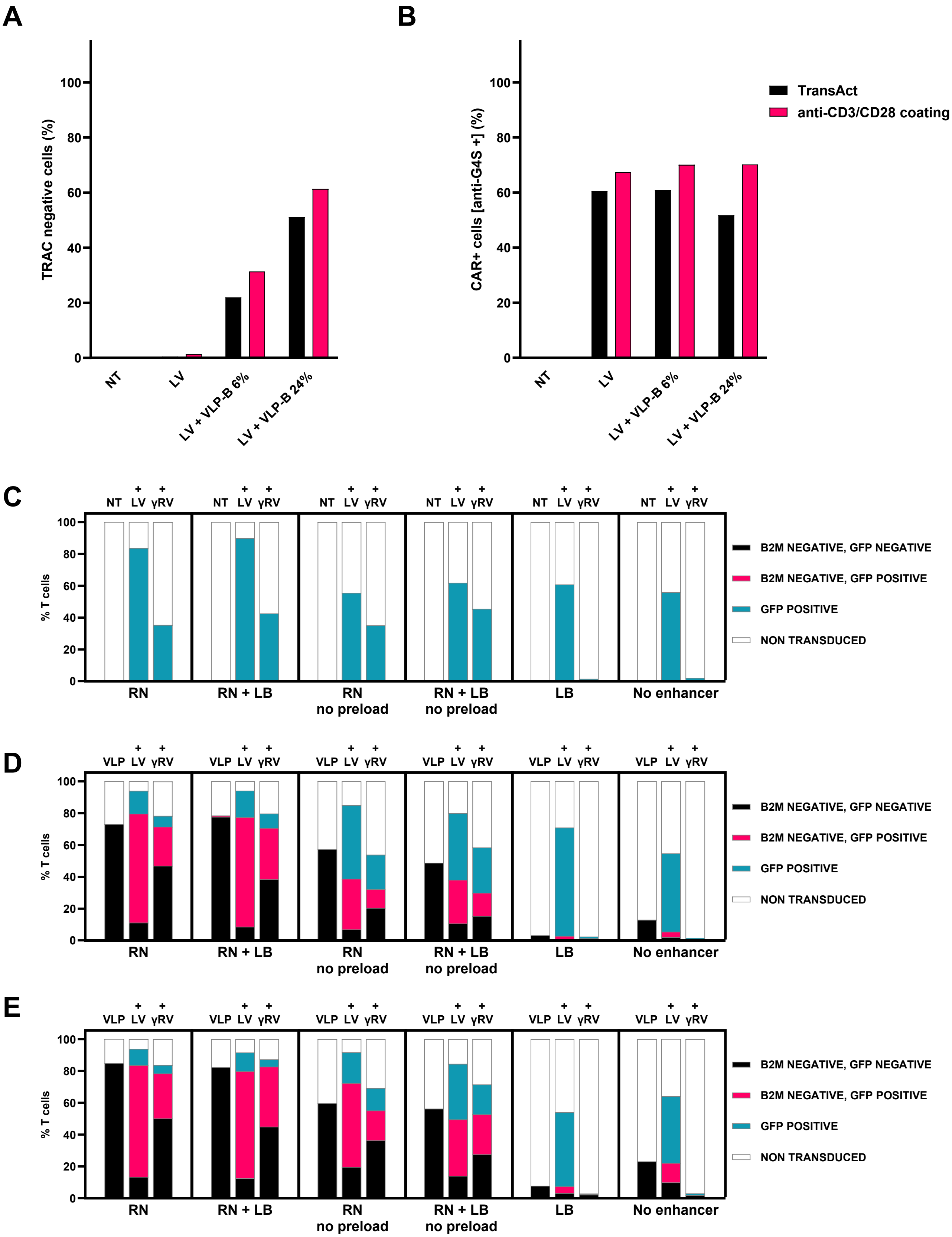
A
TRAC negative cells (%)
0
20
40
60
80
100
NT
LV
LV + VLP-B 6%
LV + VLP-B 24%
B
CAR+ cells [anti-G4S +] (%)
0
20
40
60
80
100
NT
LV
LV + VLP-B 6%
LV + VLP-B 24%
TransAct
anti-CD3/CD28 coating
C
NT
+ LV
+ γRV
% T cells
0
20
40
60
80
100
RN
RN + LB
RN no preload
RN + LB no preload
LB
No enhancer
B2M NEGATIVE, GFP NEGATIVE
B2M NEGATIVE, GFP POSITIVE
GFP POSITIVE
NON TRANSDUCED
D
VLP
+ LV
+ γRV
% T cells
0
20
40
60
80
100
RN
RN + LB
RN no preload
RN + LB no preload
LB
No enhancer
B2M NEGATIVE, GFP NEGATIVE
B2M NEGATIVE, GFP POSITIVE
GFP POSITIVE
NON TRANSDUCED
E
VLP
+ LV
+ γRV
% T cells
0
20
40
60
80
100
RN
RN + LB
RN no preload
RN + LB no preload
LB
No enhancer
B2M NEGATIVE, GFP NEGATIVE
B2M NEGATIVE, GFP POSITIVE
GFP POSITIVE
NON TRANSDUCED

Figure S4: Optimization of T-cells transduction conditions

Healthy donor T-cells were activated using TransAct™ or by incubation in anti-CD3 and anti-CD28 coated wells before being transduced with a CAR LV vector and two different quantities of VLPs pseudotyped with BaEV-Rless to edit the TRAC locus. TRAC knock-out (A), and CAR expressions (B) are measured using flow cytometry after staining with the anti-G4S and anti-TCR α/β antibodies.

The transduction enhancers are tested alone or in combination for transduction of GFP-expressing LV and γRV and 0 µL(C), 0.25 µl (D), or 0.75 µL (E) of VLP to edit the B2M locus. B2M knock-out and GFP expression were evaluated using flow cytometry. RN – RetroNectin; RN +LB – RetroNectin and LentiBOOST™; RN – RetroNectin without preloading; RN +LB – RetroNectin and LentiBOOST™ without preloading; LB – LentiBOOST. n=1

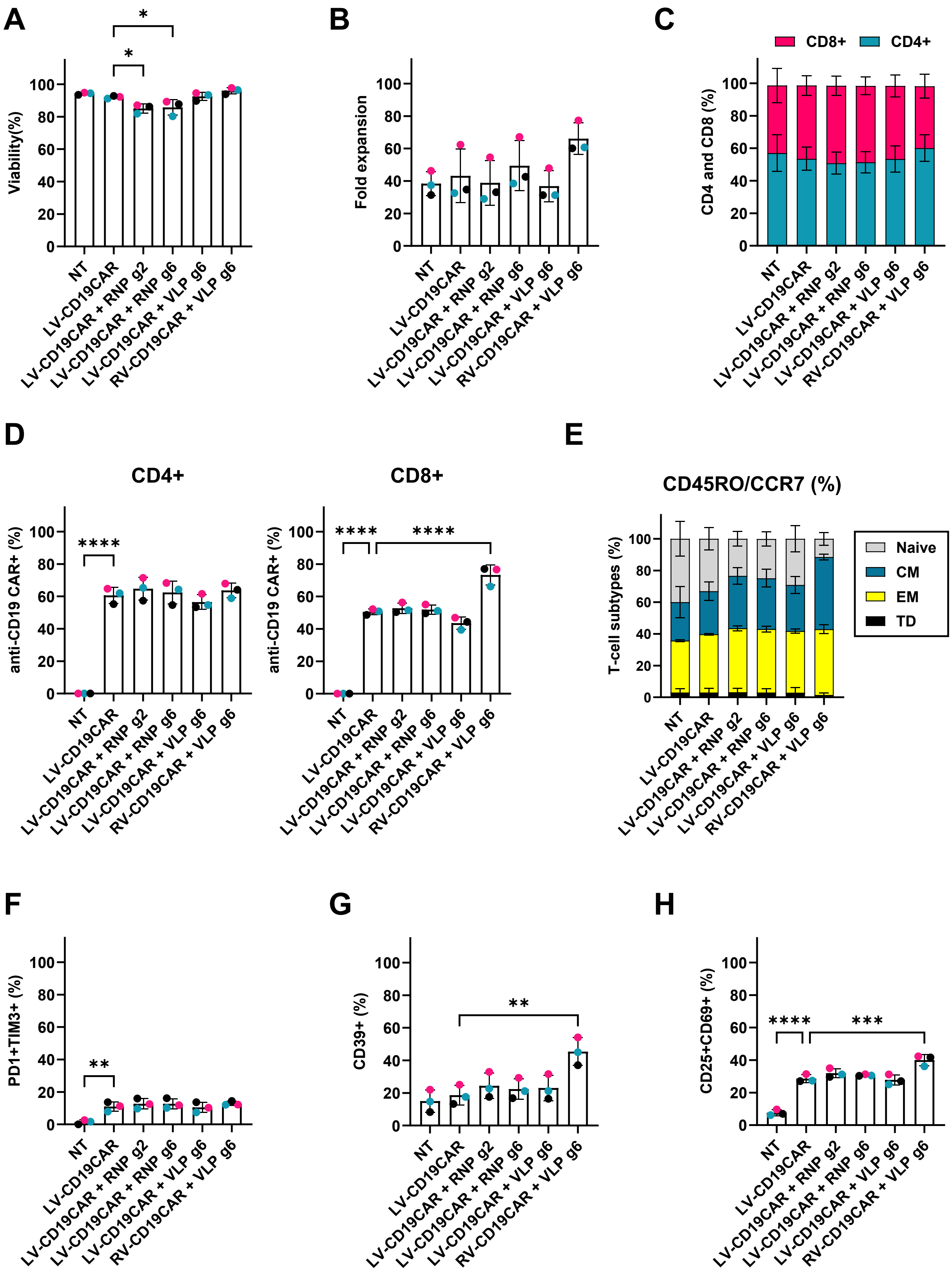
A
B
C
D
E
F
G
H
Viability(%)
Fold expansion
CD4 and CD8 (%)
CD8+
CD4+
anti-CD19 CAR+ (%)
CD45RO/CCR7 (%)
T-cell subtypes (%)
Naive
CM
EM
TD
PD1+TIM3+ (%)
CD39+ (%)
CD25+CD69+ (%)
NT
LV-CD19CAR
LV-CD19CAR + RNP g2
LV-CD19CAR + RNP g6
LV-CD19CAR + VLP g6
RV-CD19CAR + VLP g6
*
**
***
****

Figure S5: Characterization of gene edited CAR T cells.

On day 1, T cells from three healthy donors were transduced with LV vector, or co-transduced with Cas9-VLP targeting DNMT3A and LV vector, or co-transduced with Cas9-VLP targeting DNMT3A and γRV vector, or transduced with LV then electroporated with RNP the next day. On day 2, cell viability was assessed post-transduction and post-electroporation (A). On day 7, cell fold expansion was calculated (B). $CD4^+$ and $CD8^+$ cells percentages are evaluated by flow cytometry (C); CAR expression in percentage and as mean fluorescence intensity is evaluated in $CD4^+$ and $CD8^+$ cell subsets(D). T-cell subtypes were characterized based on CD45RO and CCR7 expression (E); Exhaustion markers PD-1, TIM3, and CD39 are assessed using flow cytometry (F-G). The activation markers CD25 and CD69 are also monitored (H). One-way ANOVA is used for statistical analysis (* $p \leq 0.05$; ** $p \leq 0.01$; *** $p \leq 0.001$; **** $p \leq 0.0001$). n=3

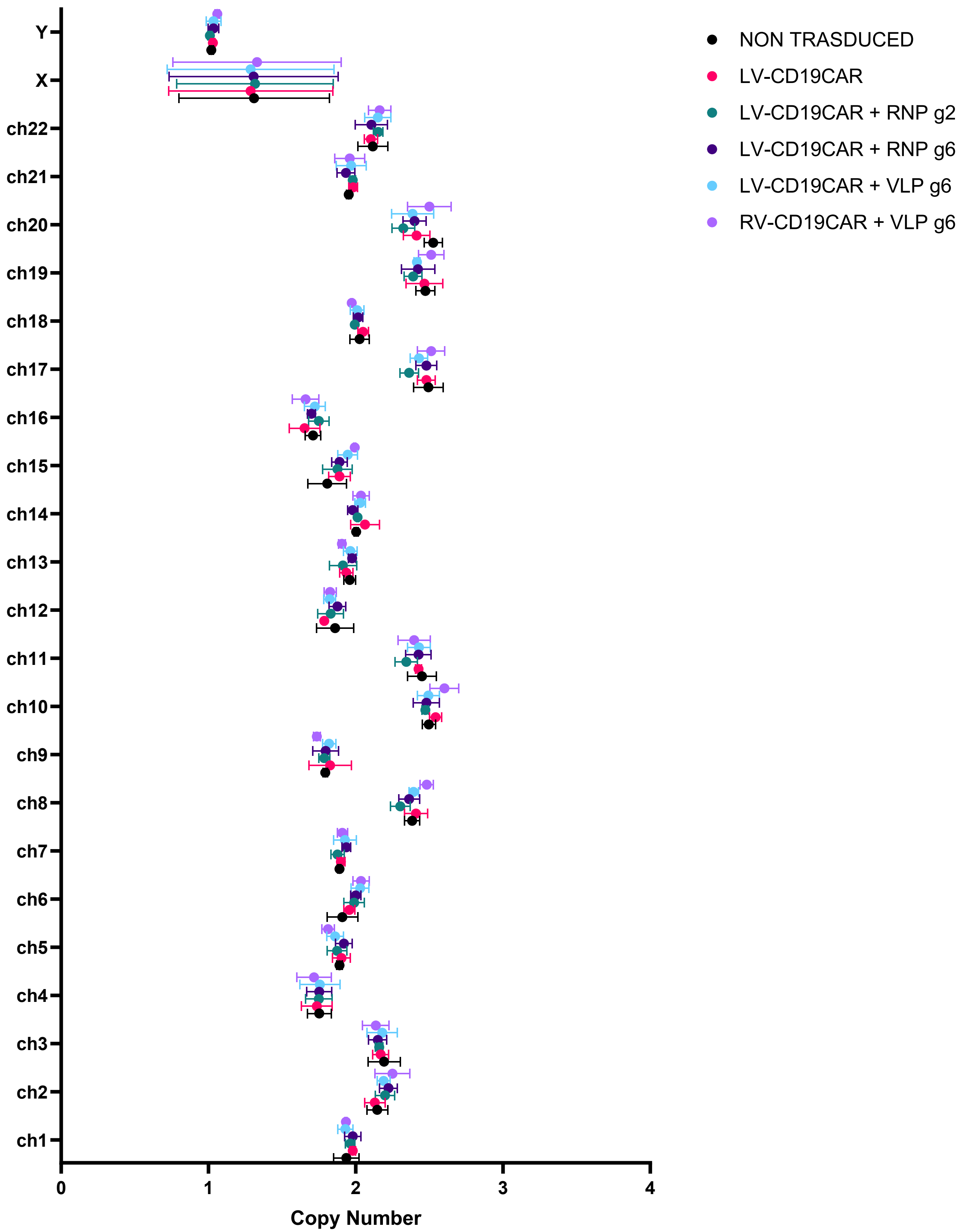
Y
X
ch22
ch21
ch20
ch19
ch18
ch17
ch16
ch15
ch14
ch13
ch12
ch11
ch10
ch9
ch8
ch7
ch6
ch5
ch4
ch3
ch2
ch1
0
1
2
3
4
Copy Number
NON TRASDUCED
LV-CD19CAR
LV-CD19CAR + RNP g2
LV-CD19CAR + RNP g6
LV-CD19CAR + VLP g6
RV-CD19CAR + VLP g6

Figure S6: Aneuploidy analysis of edited CAR T-cells

Genomic DNA of T-cells from three healthy donors co-transduced with an LV or γRV vector expressing CAR and edited by electroporation of RNPs or transduction using VLP was extracted and analyzed using the iCS-digital™ PSC: Aneuploidy test (Stem Genomics). Genomic DNA from paired, non-transduced cells were used as a control and as a baseline to establish chromosome copy number. Chromosome copy number with standard deviation was plotted for each chromosome. Standard deviations for the X chromosome were higher because one donor is female (two copies), and two donors are male (1 copy).

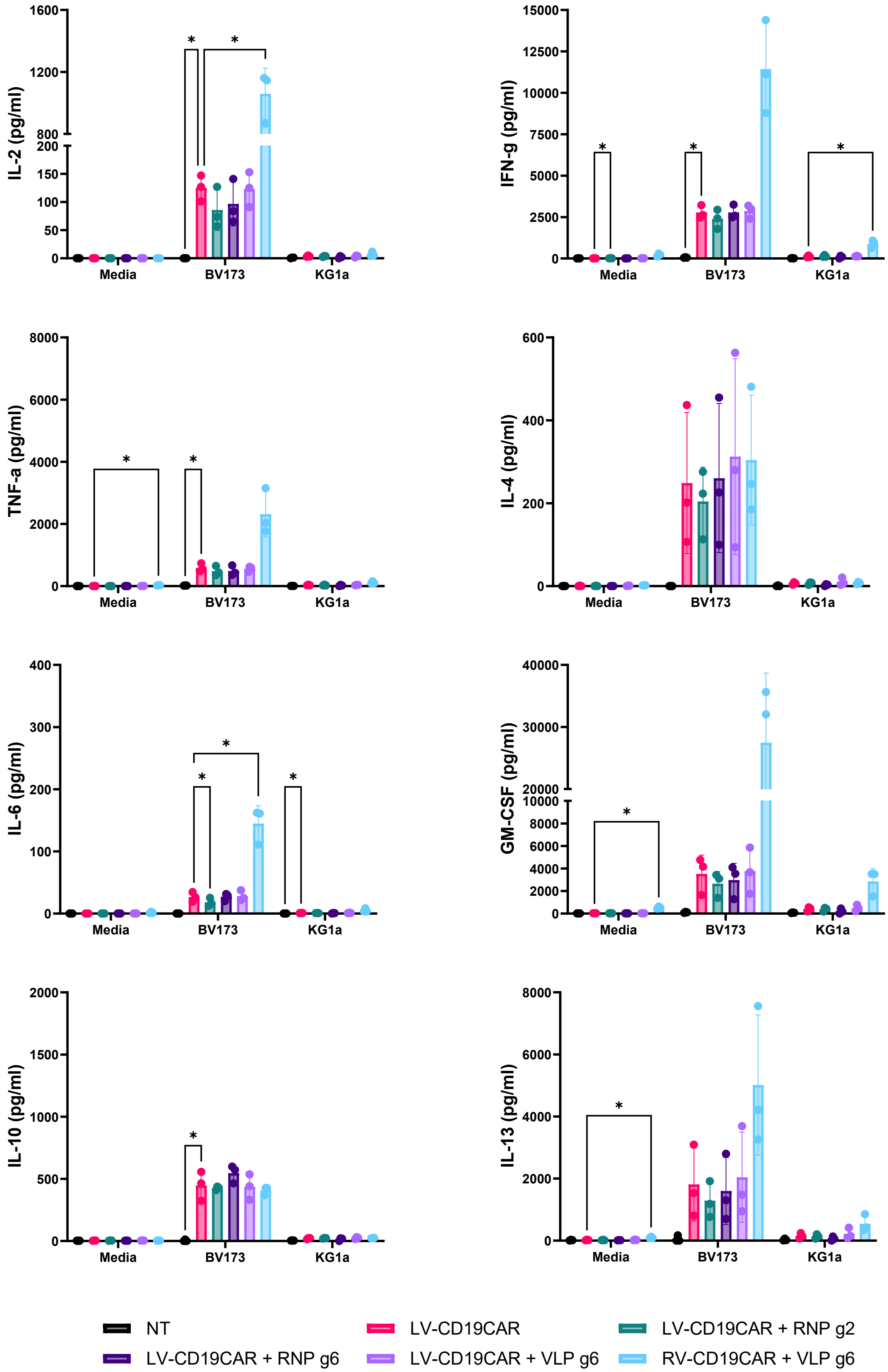
1600
1200
800
200
150
100
50
0
IL-2 (pg/ml)
Media
BV173
KG1a
15000
12500
10000
7500
5000
2500
0
IFN-g (pg/ml)
8000
6000
4000
2000
TNF-a (pg/ml)
600
400
200
IL-4 (pg/ml)
400
300
IL-6 (pg/ml)
40000
30000
20000
10000
GM-CSF (pg/ml)
2000
1500
1000
500
IL-10 (pg/ml)
IL-13 (pg/ml)
NT
LV-CD19CAR
LV-CD19CAR + RNP g2
LV-CD19CAR + RNP g6
LV-CD19CAR + VLP g6
RV-CD19CAR + VLP g6

Figure S7: Cytokine secretion during in vitro CAR-T cell–mediated killing

DNMT3A-edited CD19 CAR T cells generated using either ribonucleoprotein (RNP) or virus-like particle (VLP) delivery were co-cultured with CD19-positive (BV173) or CD19-negative (KG1a) target cells overnight. Following co-culture, supernatants were collected and analyzed for release of IL-2, IFN-γ, TNF-α, IL-4, IL-6, GM-CSF, IL-10, and IL-13. Two-way ANOVA is used for statistical analysis (* $p \leq 0.05$; ** $p \leq 0.01$; *** $p \leq 0.001$; **** $p \leq 0.0001$). n=3

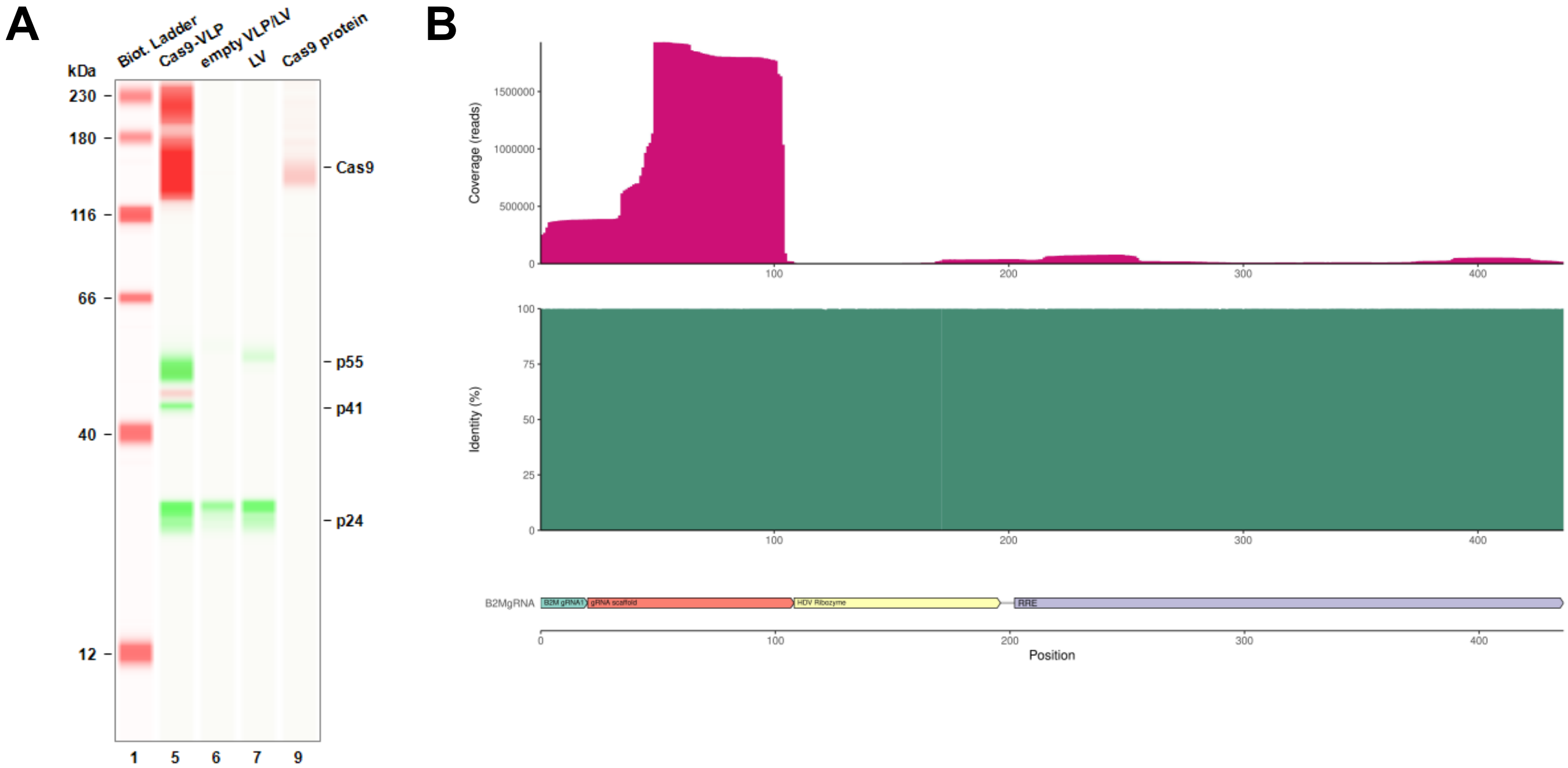

Figure S8: Characterization of Virus-Like particles

Cas9 and p24 in VLPs were detected by capillary western blotting (A). The B2M sgRNA loaded on VLP was extracted and subjected to next-generation sequencing and bioinformatic analysis to align with expected sequence (B).

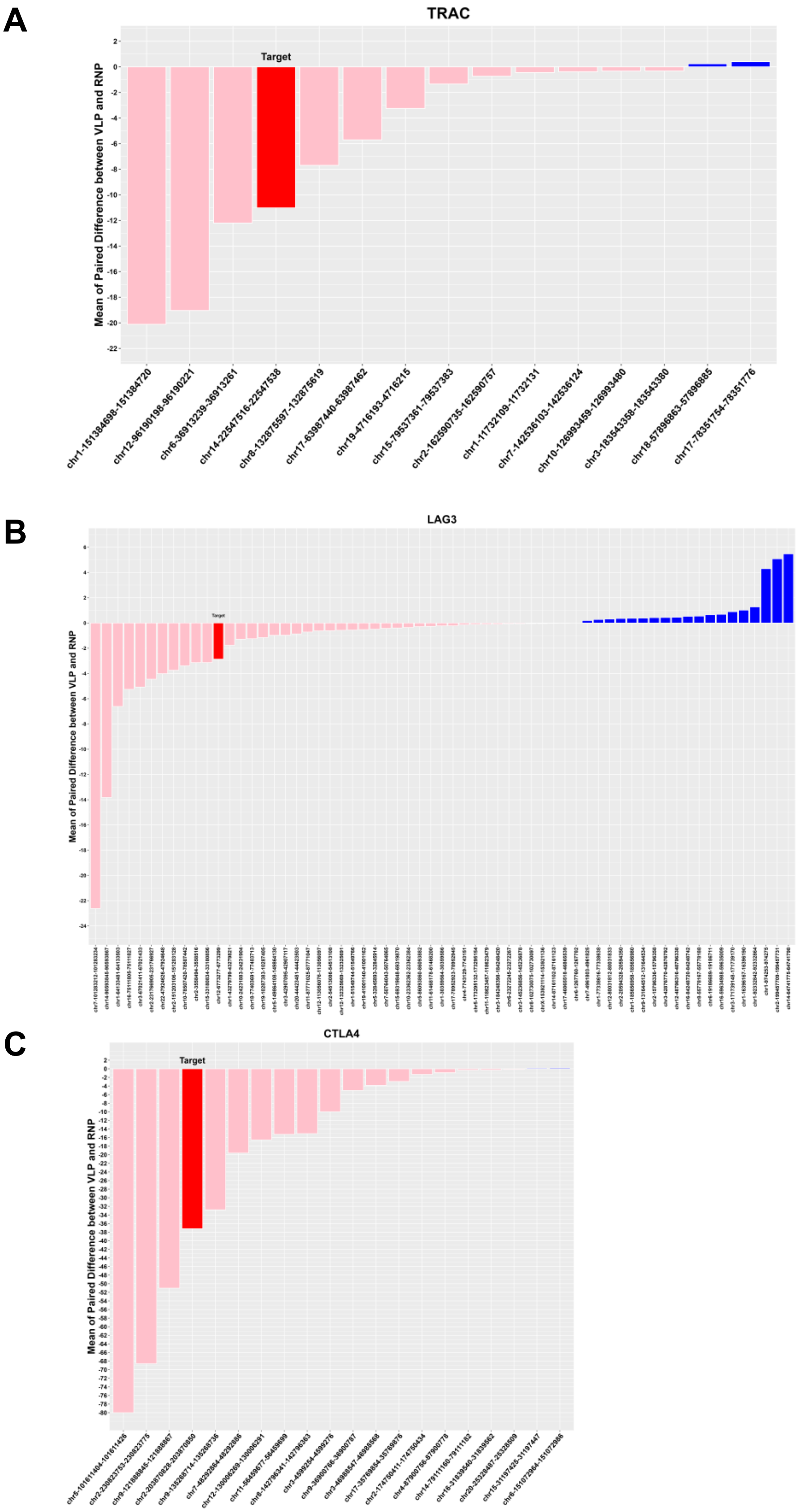
A
TRAC
Target
Mean of Paired Difference between VLP and RNP
chr1-151384698-151384720
chr12-96190198-96190221
chr6-36913239-36913261
chr14-22547516-22547538
chr8-132875597-132875619
chr17-63987440-63987462
chr19-4716193-4716215
chr15-79537361-79537383
chr2-162590735-162590757
chr1-11732109-11732131
chr7-142536103-142536124
chr10-126993459-126993480
chr3-183543358-183543380
chr18-57896863-57896885
chr17-78351754-78351776
B
LAG3
Target
Mean of Paired Difference between VLP and RNP
C
CTLA4
Target
Mean of Paired Difference between VLP and RNP
chr5-101611404-101611426
chr2-230823753-230823775
chr9-121888845-121888867
chr2-203870828-203870850
chr9-135268714-135268736
chr7-48292864-48292886
chr12-130006269-130006291
chr11-56459677-56459699
chr8-142796341-142796363
chr3-4599254-4599276
chr9-36900766-36900787
chr3-46988547-46988568
chr17-35769854-35769876
chr2-174750411-174750434
chr4-87900756-87900778
chr14-79111160-79111182
chr16-31839540-31839562
chr20-25328487-25328509
chr15-31197425-31197447
chr6-151072964-151072986

Figure S9: Mean of paired difference between off-targets detected after editing using Cas9-VLPs and electroporated RNPs

After curation of rhAmpSeq data for TRAC (A), LAG3 (B), and CTLA-4 (C), the mean of paired difference was calculated between Cas9-VLPs editing and electroporated RNPs editing for every in which editing was considered significant (above 7%). The means of paired differences were graphed and ordered to highlight which loci had significantly different editing between the two groups.

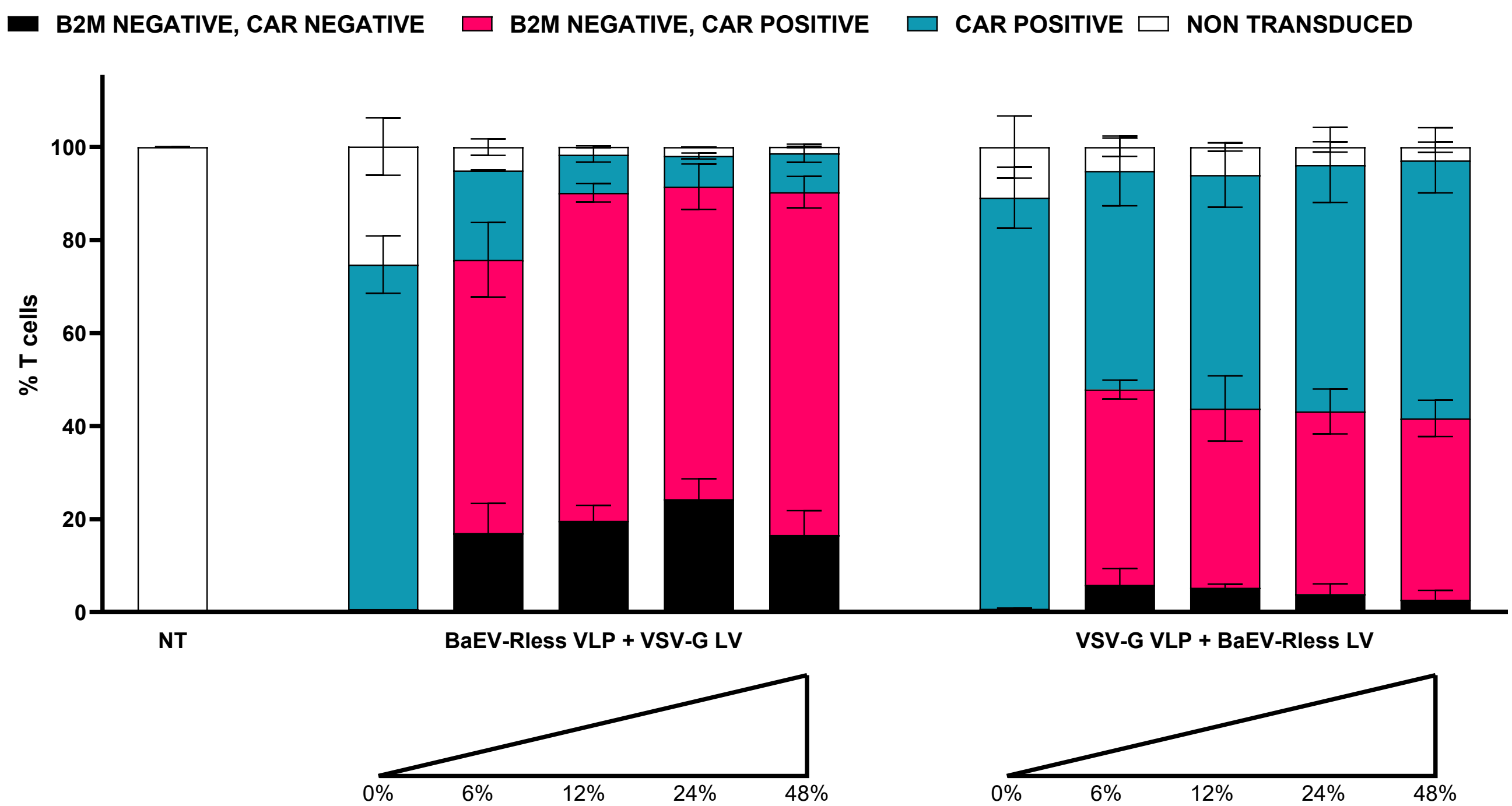

Figure S10: Evaluation of VSV-G and BaEV-Rless for pseudotyping LV and VLP

Healthy human T cells were co-transduced with either LV pseudotyped with VSV-G and an increasing amount of VLP pseudotyped with BaEV-Rless, or with LV pseudotyped with BaEV-Rless, and an increasing amount of VLP pseudotyped with VSV-G. Seven days post-transduction, CAR and TRAC expression were assessed by cell surface staining using an anti-G4S and anti-B2M. n=2

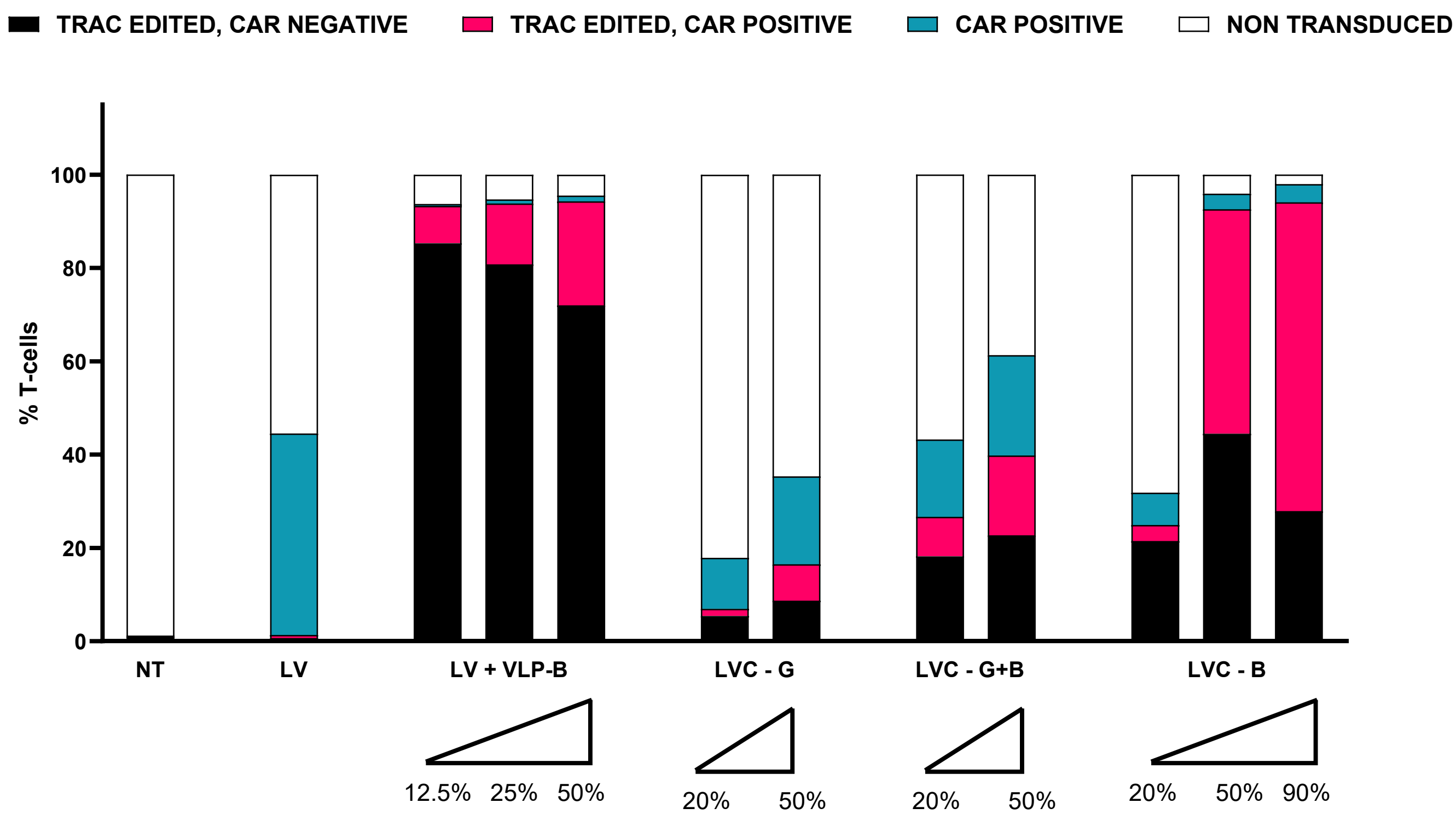

Figure S11: Comparison of transduction of LVC pseudotyped with different glycoproteins
Healthy human T cells were transduced with either VSV-G LV only, VSV-G LV and BaEV-Rless VLP, LVC pseudotyped with VSV-G, LVC pseudotyped with VSV-G and BaEV-Rless, or LVC pseudotyped with BaEV-Rless. LV and LVC expressed a bi-specific anti-CD19CD22 CAR. VLP and LVC targeted the TRAC locus. Seven days post-transduction, CAR and TRAC expression were assessed by cell surface staining using an anti-G4S and anti-TCR α/β antibodies. n=1

Table S1: Characterization of Virus-Like Particle Preparations used in healthy-donor T-cells transductions

Unique ID	Type	Envelope	Target	Figure	p24 (µg/mL)	Cas9 (µg/mL)	Average p24 (µg/mL)	Standard Deviation p24 (µg/mL)	Average Cas9 (µg/mL)	Standard Deviation Cas9 (µg/mL)
**13014**	LVC	VSV-G	B2M	2	68.50	5.17	54.43	30.26	4.09	2.21
**13210**	LVC	VSV-G	TRAC	2	19.70	1.55				
**13719**	LVC	VSV-G	DNMT3A	3	75.10	5.57				
**13800**	VLP	BaEV-Rless	B2M	2	201.61	5.16	175.29	45.14	8.66	4.08
**13207**	VLP	BaEV-Rless	TRAC	2	107.71	5.19				
**13724**	VLP	BaEV-Rless	DNMT3A	3	197.00	11.25				
**13958**	VLP	BaEV-Rless	DNMT3A	5, 6, S5	194.83	13.03				
**13798**	VLP	VSV-G	B2M	2	123.41	5.45	80.37	52.20	4.25	2.26
**13204**	VLP	VSV-G	TRAC	2	22.30	1.65				
**13723**	VLP	VSV-G	DNMT3A	3	95.41	5.66				

Table S2: Raw p-value and adjusted p-value of the paired t-test performed during the rhAmpSeq analysis for editing of TRAC locus by VLPs or electroporated RNPs

TargetID	Target Category	Raw P-value	Adjusted P-value
**chr14-22547516-22547538**	On-target	0.0182	
**chr1-151384698-151384720**	Off-target	0.0007	0.0045
**chr6-36913239-36913261**	Off-target	0.0007	0.0045
**chr12-96190198-96190221**	Off-target	0.0010	0.0045
**chr8-132875597-132875619**	Off-target	0.0046	0.0160
**chr17-63987440-63987462**	Off-target	0.0070	0.0196
**chr19-4716193-4716215**	Off-target	0.0107	0.0249
**chr15-79537361-79537383**	Off-target	0.0778	0.1557
**chr2-162590735-162590757**	Off-target	0.2004	0.3508
**chr7-142536103-142536124**	Off-target	0.3501	0.5331
**chr3-183543358-183543380**	Off-target	0.3808	0.5331
**chr1-11732109-11732131**	Off-target	0.4217	0.5367
**chr10-126993459-126993480**	Off-target	0.4738	0.5527
**chr17-78351754-78351776**	Off-target	0.6446	0.6942
**chr18-57896863-57896885**	Off-target	0.8115	0.8115

Table S3: Raw p-value and adjusted p-value of the paired t-test performed during the rhAmpSeq analysis for editing of LAG3 locus by VLPs or electroporated RNPs

TargetID	Target Category	Raw P-value	Adjusted P-value
chr12-6773277-6773299	On-target	0.2454	
chr2-231766905-231766927	Off-target	0.0011	0.0686
chr16-75111805-75111827	Off-target	0.0032	0.0983
chr7-101283212-101283234	Off-target	0.0116	0.2399
chr14-90593845-90593867	Off-target	0.0223	0.3418
chr1-43279799-43279821	Off-target	0.0298	0.3418
chr2-151203106-151203128	Off-target	0.0395	0.3418
chr1-64133481-64133503	Off-target	0.0422	0.3418
chr15-33180834-33180856	Off-target	0.0486	0.3418
chr22-47924626-47924648	Off-target	0.0551	0.3418
chr3-67021411-67021433	Off-target	0.0551	0.3418
chr16-59634988-59635009	Off-target	0.0617	0.3476
chr1-16396167-16396190	Off-target	0.0717	0.3614
chr12-80031812-80031833	Off-target	0.0758	0.3614
chr5-149864108-149864130	Off-target	0.1202	0.5323
chr1-185656958-185656980	Off-target	0.1828	0.7554
chr3-171739148-171739170	Off-target	0.1959	0.7591
chr8-55778167-55778188	Off-target	0.2535	0.8982
chr20-44423481-44423503	Off-target	0.2714	0.8982
chr1-92332842-92332864	Off-target	0.2945	0.8982
chr12-132325669-132325691	Off-target	0.3109	0.8982
chr2-15796336-15796358	Off-target	0.3144	0.8982
chr18-77403691-77403713	Off-target	0.3357	0.8982
chr7-4961803-4961825	Off-target	0.3468	0.8982
chr2-199457709-199457731	Off-target	0.3516	0.8982
chr12-48796316-48796338	Off-target	0.3636	0.8982
chr16-84248720-84248742	Off-target	0.4103	0.8982
chr2-3558494-3558516	Off-target	0.4243	0.8982
chr10-23362362-23362384	Off-target	0.4269	0.8982
chr2-54513086-54513108	Off-target	0.4633	0.8982
chr10-24231883-24231904	Off-target	0.4634	0.8982
chr14-64741775-64741798	Off-target	0.4726	0.8982
chr12-113056076-113056097	Off-target	0.4966	0.8982
chr10-78507420-78507442	Off-target	0.5031	0.8982
chr7-50764043-50764065	Off-target	0.5056	0.8982
chr3-42907095-42907117	Off-target	0.5070	0.8982
chr17-78952923-78952945	Off-target	0.5323	0.9068
chr19-41001140-41001162	Off-target	0.5537	0.9068
chr15-69319848-69319870	Off-target	0.5631	0.9068
chr3-42876770-42876792	Off-target	0.5897	0.9068

Tables S3 continued

TargetID	Target Category	Raw P-value	Adjusted P-value
chr5-86093860-86093882	Off-target	0.6139	0.9068
chr2-20594328-20594350	Off-target	0.6168	0.9068
chr5-32845893-32845914	Off-target	0.6224	0.9068
chr1-30359564-30359586	Off-target	0.6289	0.9068
chr11-87771025-87771047	Off-target	0.6512	0.9176
chr11-61468178-61468200	Off-target	0.6852	0.9434
chr9-131644512-131644534	Off-target	0.7000	0.9434
chr11-118623457-118623479	Off-target	0.7570	0.9816
chr4-7743129-7743151	Off-target	0.7776	0.9816
chr3-184248398-184248420	Off-target	0.8039	0.9816
chr6-19186689-19186711	Off-target	0.8102	0.9816
chr5-173295132-173295154	Off-target	0.8358	0.9816
chr3-145236856-145236878	Off-target	0.8519	0.9816
chr1-974253-974275	Off-target	0.8539	0.9816
chr1-77338616-77338638	Off-target	0.8701	0.9816
chrX-153921114-153921136	Off-target	0.8708	0.9816
chr1-51549744-51549766	Off-target	0.8921	0.9876
chr6-23272245-23272267	Off-target	0.9107	0.9905
chr8-102730075-102730097	Off-target	0.9266	0.9905
chr19-10287383-10287405	Off-target	0.9612	0.9936
chr6-1267760-1267782	Off-target	0.9831	0.9936
chr17-46865518-46865539	Off-target	0.9834	0.9936
chr14-87161102-87161123	Off-target	0.9936	0.9936

Table S4: Raw p-value and adjusted p-value of the paired t-test performed during the rhAmpSeq analysis for editing of CTLA-4 locus by VLPs or electroporated RNPs

TargetID	Target Category	Raw P-value	Adjusted P-value
**chr2-203870828-203870850**	On-target	0.0020	
**chr2-230823753-230823775**	Off-target	0.0005	0.0089
**chr5-101611404-101611426**	Off-target	0.0023	0.0177
**chr9-135268714-135268736**	Off-target	0.0028	0.0177
**chr11-56459677-56459699**	Off-target	0.0038	0.0181
**chr9-121888845-121888867**	Off-target	0.0080	0.0255
**chr3-4599254-4599276**	Off-target	0.0101	0.0255
**chr12-130006269-130006291**	Off-target	0.0105	0.0255
**chr7-48292864-48292886**	Off-target	0.0107	0.0255
**chr8-142796341-142796363**	Off-target	0.0373	0.0787
**chr17-35769854-35769876**	Off-target	0.0421	0.0799
**chr2-174750411-174750434**	Off-target	0.0811	0.1401
**chr9-36900766-36900787**	Off-target	0.0946	0.1457
**chr4-87900756-87900778**	Off-target	0.0997	0.1457
**chr3-46988547-46988568**	Off-target	0.1305	0.1771
**chr16-31839540-31839562**	Off-target	0.5753	0.7287
**chr14-79111160-79111182**	Off-target	0.6177	0.7335
**chr15-31197425-31197447**	Off-target	0.8981	0.9538
**chr6-151072964-151072986**	Off-target	0.9036	0.9538
**chr20-25328487-25328509**	Off-target	0.9779	0.9779

Table S5: Sequences of the primers used to generate the gRNA-expressing plasmid and for Targeted Deep Sequencing amplicons

Target	gRNA	Protospacer	Sequence-specific primer for mutagenesis	Targeted Deep Sequencing FOR primer	Targeted Deep Sequencing REV primer
**TRAC**	g2	GCTGGTACACGGCAGGGTCA	-	TCCCAGTCCATCACGAGCAGCTGGT	TTGCTCCAGGCCACAGCACTGTTGC
**TRAC**	g3	CAGGGTTCTGGATATCTGT	ACAGATATCCAGAACCCTGCGGTGTTTCGTCCTTTCC	TCCCAGTCCATCACGAGCAGCTGGT	TTGCTCCAGGCCACAGCACTGTTGC
**B2M**	g1	GAGTAGCGCGAGCACAGCTA	TAGCTGTGCTCGCGCTACTCGGTGTTTCGTCCTTTCC	TGGCTTGGAGACAGGTGACGGT	GGAGAACTTGGAGAAGGGAAGTCACG
**DNTM3A**	g2	CCTGCATGATGCGCGGCCCA	TGGGCCGCGCATCATGCAGGCGGTGTTTCGTCCTTTCC	TCCCGATGACCCTGTCTTCCCGTGC	AGGTAGAAGCCATTAGTGAGCTGGC
**DNTM3A**	g6	GCACTGCAAAACGAGCTCAG	CTGAGCTCGTTTTGCAGTGCGGTGTTTCGTCCTTTCC	GGTCTGGACTCCTCTTTGCATCGGGT	GCGAGGTGGAGAGAAAGGAGTCGCT
**LAG3**	g9	GAAGGCTGAGATCCTGGAGG	-	CCTGCACCCTGTTTCTCCCTCGGGA	GAGGGAGTGACACCTCAGGGCAGGG
**CTLA-4**	g9	GGACTGAGGGCCATGGACAC	-	GCTTCGGCAGGCTGACAGC	GCATAGAAGACTGCAATGCAACAGGT

Table S6: Antibodies used in the study

Target	Dye	Clone	Manufacturer	Cat#
**Biotin-APC**	APC	REA746	Miltenyi Biotec	130-110-952
**Cas9**	-	Polyclonal	Takara	632607
**CCR7**	FITC	REA546	Miltenyi Biotec	130-120-468
**CD19-biotin**	-	-	Miltenyi Biotec	130-129-550
**CD25**	PE	4E3	Miltenyi Biotec	130-113-282
**CD39**	BV605	A1	Biolegend	328236
**CD4**	BV785	SK3	Biolegend	344642
**CD4**	APC-Cy7	RPA-T4	BD Bioscience	561841
**CD45RO**	BV650	UCHL1	Biolegend	304232
**CD69**	APC-Cy7	FN50	Biolegend	310914
**CD8**	BV510	SK1	BD Bioscience	563919
**DNMT3A**	-	Polyclonal	GeneTex	GTX129125
**G4S Linker**	PE	E7O2V	Cell Signaling Technology	71645
**GAPDH**	-	0411	Santa Cruz	sc-47724
**HIV-1 Gag p24**	-	749122	R&D Systems Inc	MAB73602
**PD1**	PerCP5.5	EH12.2H7	Biolegend	329914
**TCR α/β**	APC	IP26	Biolegend	306718
**TIM3**	PE-Cy7	F38-2E2	Biolegend	345014
**β2-microglobulin**	PE-Cy™7	2M2	Biolegend	316318
