# Supplementary material for: Co-delivery of lentiviral vectors and Cas9-containing virus-like particles enables rapid, scalable manufacture of gene-edited CAR T cells
Source: Mol Ther Adv. 2026 Jul 9;34(3):201809. doi: 10.1016/j.omta.2026.201809 (PMC13446315; doi:10.1016/j.omta.2026.201809)
Supplement: Supplementary file 2 [file mmc2.pdf]

Molecular Therapy
Advances
Original Article

Co-delivery of lentiviral vectors and Cas9-containing virus-like particles enables rapid, scalable manufacture of gene-edited CAR T cells

Francesca Ferrara,[1] Matthew M. Wielgosz,[1] Jeoungeun J. Park,[2] Matthew Bauler,[1] Chris Wincek,[1] Nataly Mier,[1] Yin Su,[3] Li Tang,[3,5] Brandon R. Lowe,[1] Jordan A. Beard,[1] Deanna M. Langfitt,[4] Sheng Zhou,[2] and Robert E. Throm[1]

[1]St. Jude Vector Laboratory, St. Jude Children's Research Hospital, Memphis, TN 38105, USA; [2]Experimental Cellular Therapeutics Laboratory, St. Jude Children's Research Hospital, Memphis, TN 38105, USA; [3]Department of Biostatistics, St. Jude Children's Research Hospital, Memphis, TN 38105, USA; [4]Department of Bone Marrow Transplantation and Cellular Therapy, St. Jude Children's Research Hospital, Memphis, TN 38105, USA

Chimeric antigen receptor T cell immunotherapies are transforming therapies for hematological malignancies and solid tumors and can be enhanced by targeted gene knockout. Here, we report lentiviral-based virus-like particles that package and deliver Cas9 ribonucleoproteins to primary human T cells. Using distinct pseudotyping strategies for virus-like particles and for lentiviral or γ-retroviral vectors, we achieved chimeric antigen receptor expression and targeted gene disruption. Under optimized transduction conditions, more than 50% of T cells expressed a chimeric antigen receptor by flow cytometry, with vector copy numbers exceeding two. Editing efficiencies were above 70% at three different target loci tested: T cell receptor α constant chain, β2-microglobulin, and DNA methyltransferase 3α. When the editing efficiency of virus-like particles was directly compared to electroporation, electroporation achieved a higher editing efficiency (99% versus 70%–90%). However, virus-like particle treatment resulted in twice as many cells being recovered compared with electroporation with a 10% increase in cell viability. Furthermore, off-target editing in virus-like particle-treated cells was reduced compared to ribonucleoprotein electroporated cells. These results support the feasibility of using virus-like particle-mediated delivery of Cas9 ribonucleoprotein to disrupt genes of interest, enabling a more scalable and cost-effective process for generating T cell immunotherapies.

INTRODUCTION

Chimeric antigen receptor (CAR) T cell immunotherapies are revolutionizing the treatment of hematological malignancies and solid tumors. The United States Food and Drug Administration and the European Medicines Agency have now approved five CD19-targeted CAR T cell therapies for B cell malignancies, and two B cell maturation antigen-targeted CAR T cell therapies for treating multiple myeloma.[1] Development of new more effective CAR T therapies is under intense investigation by researchers. There are currently more than 1,800 CAR T clinical trials registered on ClinicalTrials.gov with over 200 new clinical trials registered in 2024 alone, which is a testament to the promise of this therapeutic approach.[2]

From these studies, several barriers to CAR T cell immunotherapy reaching its full potential were identified, including limited T cell expansion and persistence, antigen escape, and tumor microenvironment, which can restrict the efficacy and application of these therapies.[3] Furthermore, the labor-intensive manufacturing and the challenge of obtaining high-quality products from manipulated patient T cells, which are sometimes recovered in low numbers from apheresis, could suffer biological dysfunction or exhaustion, resulting in a low response rate.[4,5]

In order to improve CAR T cell immunotherapy, different strategies are being pursued: (1) the use of complex vector designs to express multiple CARs and avoid antigen escape, (2) or express cytokines and chemokine receptors to increase trafficking and homing to the tumor site; (3) the use of inducible and more regulated systems to reduce toxicities and increase specificity; (4) the modulation and modification of T cell phenotypes with small molecule drugs or gene editing approaches.[2,4,6–12]

Clustered regularly interspaced short palindromic repeats (CRISPR)-Cas9 gene editing and base editing approaches are compelling tools to enhance CAR T cell activity, increase their persistence and potency, and avoid exhaustion.[4,13] *In vivo* studies using xenograft models have shown that loss of programmed cell death protein 1 (PD-1) expression in T cells can improve the clearance of tumors expressing the inhibitory molecule programmed death-ligand 1. Furthermore, loss of DNA methyltransferase 3 alpha (DNMT3A) expression by Cas9 knockout has been shown to preserve CAR T cell proliferation and differentiation capacities, resulting in the CAR T cells maintaining a long-lasting effector function

Received 26 February 2026; accepted 8 July 2026;
https://doi.org/10.1016/j.omta.2026.201809.

[5]Present address: Department of Quantitative Health Sciences, Cleveland Clinic, Cleveland, OH, 44195, USA

Correspondence: Robert E. Throm, St. Jude Vector Laboratory, St. Jude Children's Research Hospital, 262 Danny Thomas Place, Memphis, TN 38105, USA.
E-mail: robert.throm@stjude.org

© 2026 The Author(s).
Published by Elsevier Inc. on behalf of The American Society of Gene and Cell Therapy.
This is an open access article under the CC BY license (http://creativecommons.org/licenses/by/4.0/).

under chronic antigen exposure in repeat stimulation assays *in vitro* and enhancing anti-tumor activity in *in vivo* models.[14] Cas9-mediated gene editing has also enabled the therapeutic potential of CAR T cells for the treatment of T cell acute lymphoblastic leukemia: one such example is the knockout of CD7 to prevent CAR T cell fratricide.[15–18] Lastly, Cas9-mediated gene editing can be used to bypass issues related to the production of CAR T cells from autologous sources, such as insufficient T cell expansion and dosing: allogeneic universal “off-the-shelf” CAR T cells can be produced and administered to multiple patients through knockout of the T cell receptor (TCR) α and TCRβ constant chains (TRAC and TRBC) and beta-2 microglobulin (B2M).[16,19,20]

To date, electroporation is the most used technique to deliver Cas9 and single-guide RNA (sgRNA) ribonucleoprotein (RNP) complexes into cells. However, there are disadvantages to this approach: the toxicity of the electroporation process results in reduced cell recovery and viability, potentially extending culture times to obtain sufficient therapeutic CAR T cell doses, which could increase T cell exhaustion.[21,22] The rising costs of specialized equipment and reagents for clinical application may also limit patient access, especially in low and middle-income countries. Furthermore, it has been shown that electroporation following lentiviral vector (LV) delivery, which currently requires T cell activation for efficient transduction, can result in chromosomal abnormalities whose long-term effects remain unknown.[23–25] Thus, novel delivery methods that can avoid the drawbacks of electroporation while remaining readily amenable to current good manufacturing practice (cGMP) settings are urgently needed.

One approach is to use virus-like particles (VLPs), which lack a viral genome, to deliver Cas9-RNP to cells by linking the Cas9 protein to the viral structural proteins and by expressing the sgRNA during particle production. Several groups have described strategies for using VLPs to deliver Cas9 RNP to cell lines and primary cells, including T- and natural killer (NK)-cells.[21,22,26–34] Early approaches to enable protein co-packaging for editing have focused on fusing Cas9 or other editing proteins directly to the Gag portion of retroviruses, usually matrix,[26] nucleocapsid,[21,27] or p6,[22,29] or on recruiting the editor at the same sites using dimerization systems or aptamers.[35,36] Subsequently, these approaches were further refined to optimize activity or improve delivery through direct cell targeting.[28,32–34] For a more comprehensive review of editing VLPs, their biology, and development, we refer the reader to the recent publication by Son et al.[37] Moreover, while current reports have shown that VLPs can efficiently deliver Cas9-RNP to cells, achieving clinically significant editing *in vitro*, none of the methods described are amenable to large-scale guanosine monophosphate (GMP) manufacturing.

Here, we describe the optimized production of Cas9-RNP-containing LV VLPs in serum-free suspension culture and their use to generate gene-edited primary human CAR T cells. We also highlight how the envelope used for pseudotyping VLPs influences their activity and how this can be exploited to simultaneously introduce the CAR-expressing cassette using LV or γ-retroviral (γ-RV) vectors, achieve high editing efficiency, and reduce T cell manipulations.

RESULTS

Cas9 fused to HIV-1 Gag-Pol allows the generation of Cas9-delivering VLPs

In retroviruses, the expression of the Gag polyprotein alone leads to VLP formation, since it encodes the structural proteins of the viral particle. Historically, to deliver a protein of interest through retroviral VLPs, the encoding sequence is placed in frame at the 3′ end of the Gag polyprotein encoding gene. However, the resulting fusion proteins remain fused to each other, potentially limiting intracellular trafficking, unless a cleavable linker and proteases are incorporated into the particle. To optimize Cas9-RNP activity after delivery, we fused Cas9 with three nuclear localization signals (3NLS)-Cas9, to the carboxyl terminus of the HIV-1 Gag-Pol polypeptide (Figures 1A and 1B). A peptide linker containing a cleavage site, which allows proteolytic separation of HIV-1 matrix and capsid protein (MA/CA cleavage) was inserted between the carboxyl terminus of HIV-1 Gag-Pol and the amino terminus of 3NLS-Cas9, with the intention that it would enable 3NLS-Cas9 release from the viral polyprotein during particle maturation (Figure S1).

It has been reported that during the production of Cas9-VLPs, sgRNA sequences are not efficiently exported from the nucleus when expressed using a U6 promoter[26]; preliminary experiments have confirmed this result in our hands (data not shown). For this reason, we designed a modified sgRNA expressing plasmid in which a Hepatitis D virus (HDV) ribozyme and the Rev-responsive element (RRE) of HIV-1 were added to the 3′ end of a modified scaffold with an extended tetraloop stem and without a premature termination sequence.[38] We hypothesize that in the presence of HIV-1 Rev, export from the nucleus would be efficient and that, subsequently, the HDV ribozyme would self-cleave, thereby removing the RRE signal and allowing the generation of a conventional sgRNA. Using this strategy, we evaluated the production of vesicular stomatitis virus glycoprotein (VSV-G) pseudotyped VLPs by transiently transfecting SJ293TS-DPB cells with two quantities (4 and 8 μg) of plasmid encoding sgRNA targeting B2M and three quantities (12, 18, or 22 μg) of HIV-1 Gag-Pol-Cas9 expressing plasmid. Cas9-VLP production was evaluated by analyzing short insertions and deletions (indels) using next-generation sequencing (NGS) at target sites after HOS-mCat1 cell transduction with serial dilutions of harvested supernatants (Figure 1C). Since, in preliminary experiments, a good correlation was observed between the percentage knockout by flow cytometry and indel percentages, titers were calculated using indel editing percentages between 1% and 25% and dilution tested; they were then expressed as gene editing unit (GEU) per mL. Although no significant differences in titers were detected in the various plasmid ratios, we chose to move forward with the conditions

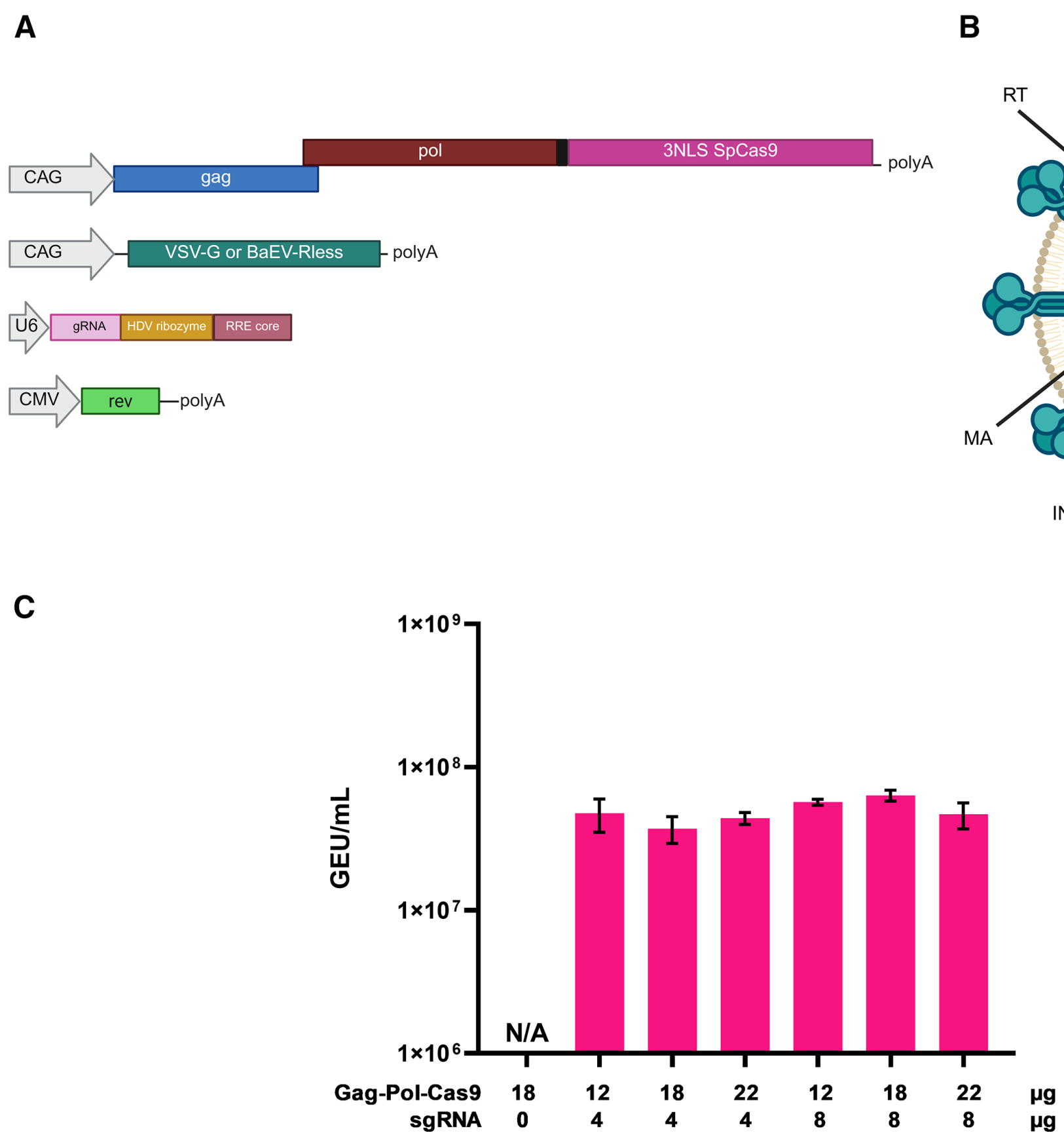

Figure 1. Production and activity of Cas9-VLPs

Design of plasmids used to produce Cas9-VLPs: a structural plasmid expressing HIV-1 Gag-Pol fused with 3NLS-Cas9; a plasmid expressing an envelope protein (VSV-G or BaEV-Rless); a plasmid expressing an sgRNA; and a plasmid encoding HIV-1 Rev (A). Schematic representation of the predicted structure of Cas9-VLP based on HIV-1 structure (B). Evaluation of Cas9-VLP gene editing activity on HOS-mCat1 cells produced using various plasmid ratios (C). Editing activity is reported as gene editing units per mL (GEU/mL). n = 3. Data are expressed as the mean ± the standard deviation.

that provided the highest overall titer, which was 18 μg of Gag-Pol-Cas9 and 8 μg of sgRNA-expressing plasmids.

To further validate our initial hypothesis regarding potential advantage in the use of sgRNA with the HDV ribozyme and the RRE, VLPs were produced using this optimized ratio and a plasmid with or without the two elements. VLP produced with sgRNA harboring the HDV ribozyme and RRE resulted in significant higher titer and editing activity evaluated by analyzing indels (Figure S2).

Fusion of Cas9 to HIV-1 Gag-Pol enables the generation of Cas9-delivering LV vectors

Next, we tested whether this particular Gag-Pol-Cas9 design could also mediate the packaging of LV genomes. To distinguish this new particle bearing both Cas9-RNP and traditional LV vector, we chose to call this LV-bearing Cas9, lentiviral vector Cas9 (LVC, Figure S3A).

To optimize LVC production from our standard LV production ratio, we reduced the amount of LV vector plasmid expressing GFP (SJL12-MND-GFP) in the transfection mix. Concurrently, the Gag-Pol-Cas9 expressing plasmid was increased to maintain a consistent amount of 18 μg of DNA between the two plasmids. Other helper plasmids, such as CMV-*rev*-AF and CAG-VSVG, were unchanged. Additionally, varying amounts of wild-type Gag-Pol expressing plasmid were spiked into the transfection reactions when 10 μg of Gag-Pol-Cas9 was tested, thereby increasing the total amount of transfected plasmid DNA. The amount of sgRNA plasmid was kept constant across all production conditions (Figure S3B). The particles generated with different plasmid ratios were then evaluated for their ability to transduce HOS-mCat1 cells, edit the B2M locus, or express GFP (Figures S3C and S3D).

In all conditions tested, a significant editing activity was detected. However, to obtain efficient delivery of the LV vector as measured by expression of GFP, it was necessary to provide wild-type Gag-Pol in *trans*, indicating that an additional unmodified Gag-Pol was required to restore particle activity in the presence of an LV genome.

Editing activity was not significantly reduced by the addition of wild-type Gag-Pol. Still, a trend of diminishing editing was observed, indicating that the presence of unmodified Gag-Pol reduced Cas9 activity.

Co-transduction of LV vector and Cas9-VLP allows high VCN and editing

Since LVC could represent an interesting approach to generate edited CAR T without the use of electroporation, we decided to compare this single drug substance approach based on LVC to a co-transduction approach in which a CAR vector is delivered by a traditional LV pseudotyped with VSV-G and the editing RNP by VLP pseudotyped with VSV-G or baboon endogenous virus glycoprotein lacking cytoplasmatic domain (BaEV-Rless).

Using TransAct to activate healthy donor T cells, and LentiBOOST as a transduction enhancer, we transduced cells with an anti-CD123 CAR LV vector using a fixed multiplicity of infection (MOI) of ∼20 and increasing volumes of VLP targeting B2M. In parallel, we tested increasing volumes of anti-CD123 CAR LVC targeting B2M. Seven days post-transduction, we evaluated CAR expression and B2M knockout using flow cytometry (Figure 2A). Four different cell populations were identified: non transduced cells, cells expressing CAR but unedited (CAR positive), cells expressing CAR and edited (B2M negative CAR positive), and cells only edited (B2M negative CAR negative). In the LVC arm, the percentage of these cell populations was the same for each amount of LVC tested, indicating no dose-response effect.

When testing co-transduction of LV vector and VLP pseudotyped with VSV-G, we observed that the percentage of CAR-positive cells decreased with increasing VLP volume, likely indicating competition for the same low-density lipoprotein receptor between LV vectors and VLP. The percentage of edited cells was also similar between the different conditions, indicating the absence of a dose-response effect when VSV-G pseudotyped VLPs were used. In contrast, when increasing amounts of BaEV-Rless pseudotyped VLPs were used with the anti-CD123 CAR LV vector, approximately 60% of cells were edited. The percentage of CAR-expressing cells was also comparable to LV vector transduction alone. Vector copy number (VCN) was also evaluated (Figure 2B). Consistent with the flow data, a reduction in the CAR VCN was observed in the VSV-G VLP group in comparison to the BaEV-Rless pseudotyped VLP, which remained mostly constant, strongly suggesting that VSV-G pseudotyped VLP outcompeted VSV-G pseudotyped LV vectors for binding to the cellular receptor.

To further confirm the trend and ensure that the observed pattern was not dependent on the particular vector or target site, we evaluated a different combination of LV vector (B7H3-CAR LV) and VLP targeting TRAC. Again, the same pattern of transduction and editing was observed (Figures 2C and 2D).

At last, we investigated the generation of edited-CAR T cells using a CD19-CD22 CAR LV vector and a sgRNA targeting DNMT3A. This sgRNA, hereafter named DNMT3A g6, was selected for optimized expression using the U6 promoter, compared with a previously described sgRNA[14] (DNMT3A g2) that efficiently knocked out DNMT3A but was unable to edit efficiently in the Cas9-VLPs context, likely due to incompatibility with U6-mediated transcription (data not shown). Since we do not have an optimized protocol for DNMT3A intracellular detection by flow cytometry, and we had independently validated the sgRNA by western blotting (data not shown), we evaluated the introduction of indels using targeted deep sequencing and used this measure as an indirect signal of knockout of DNMT3A for this experiment (Figure 2E). In this experimental setup, we observed that VLPs pseudotyped with BaEV-Rless efficiently introduced 40% of indels at the DNMT3A locus without influencing CAR expression and VCN (Figures 2F and 2G).

Optimizing transduction conditions, including T cell activation and the use of transduction enhancers, improves Cas9-VLPs editing

Having identified that BaEV-VLPs and VSV-G-LV vectors could be used together to efficiently obtain edited CAR T cells via co-transduction, we wanted to optimize our protocols to increase transduction and editing. First, we compared two different methods of T cell activation: TransAct, colloidal polymeric nano-matrix conjugated to humanized CD3 and CD28 agonists, versus anti-CD3 and anti-CD28 antibody coating on plastic surfaces. Indeed, T cells activated with anti-CD3 and anti-CD28 antibodies showed higher editing (Figure S4A) and had higher CAR expression (Figure S4B) than TransAct-activated T cells transduced with our LV-VLP combination. This is likely associated with the high percentage (>80%) of the T cell activation marker CD69 and CD25 positive cells in the anti-CD3 and anti-CD28 antibodies in comparison to the TransAct-activated T cells (∼40%) measured 1 day post-activation by flow cytometry (data not shown).

Next, we compared different transduction enhancers: LentiBOOST and RetroNectin. We also tested whether gibbon ape leukemia virus (GaLV) pseudotyped γ-RV vectors could be used to deliver a gene of interest instead of LV vectors in combination with Cas9-VLPs. In this case, for convenience, we used a GFP-expressing vector. Six different transduction protocols were evaluated: (1) pre-loading VLP and LV or γ-RV on RetroNectin-coated surface followed by supernatant removal after 2 h; (2) pre-loading of all viral particles VLP and LV or γ-RV vectors on RetroNectin-coated surface plus LentiBOOST; (3) addition of VLP and LV or γ-RV vectors to cells cultured in RetroNectin-coated wells; (4) addition of VLP and LV or γ-RV vectors on RetroNectin-coated surface in the presence of LentiBOOST; (5) using LentiBOOST during transduction only; and (6) performing transductions without enhancers (Figures S4C–S4E). The results indicated that while transduction enhancers were not strictly required for vector integration or

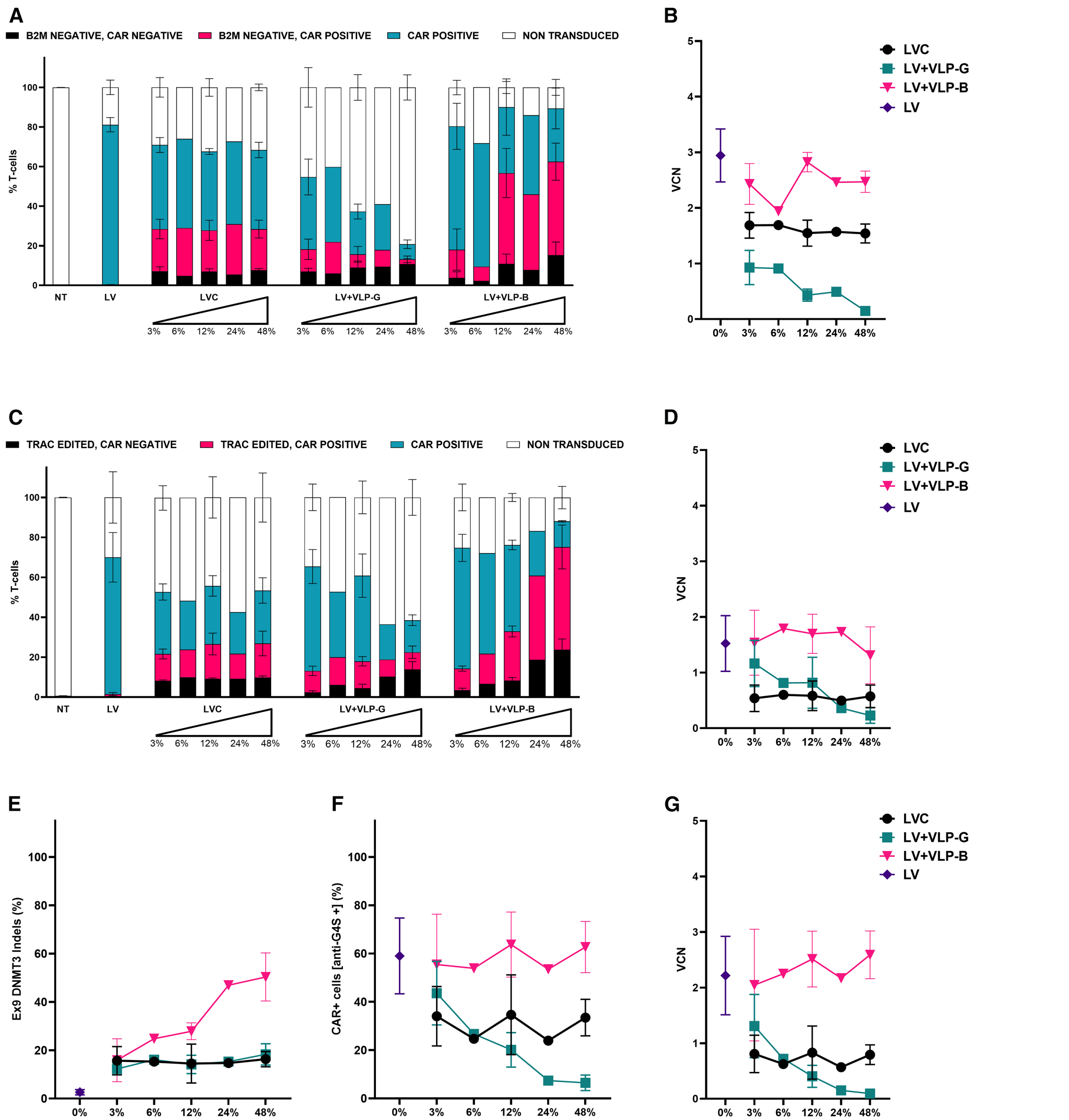

Figure 2. Comparison of LVC transduction and VLP/LV vector co-transduction for the generation of edited CAR T cells

Healthy human T cells from three different donors were transduced with either LV only, increasing amounts of LVC or co-transduced with LV and increasing amounts of Cas9-VLPs pseudotyped with either VSV-G or BaEV-Rless glycoproteins. LV and LVC expressed either an anti-CD123 CAR (A, B), anti-B7H3 CAR (C, D) or a bi-specific anti-CD19CD22 CAR (E–G). Cas9-VLP targeted either B2M (A, B), TRAC (C, D) or DNMT3A (E–G). Seven days post-transduction, CAR, B2M, and TRAC expression were assessed by cell surface staining using an anti-G4S, anti-B2M, and anti-TCR α/β antibodies, respectively (A, C, and F) followed by flow cytometry. Since DNMT3A is not expressed on the surface, indel frequency at the target site was determined by targeted deep sequencing (E). Vector copy number (VCN) was measured using ddPCR (B, D, and G). *n* = 3. Data are expressed as the mean ± the standard deviation.

gene editing, the use of RetroNectin provided the highest levels of GFP positive cells and gene editing. Interestingly, the addition of LentiBOOST reduced VLP editing efficiency and, consequently, knockdown. However, RetroNectin maximized editing and was essential for achieving high γ-RV transduction levels.

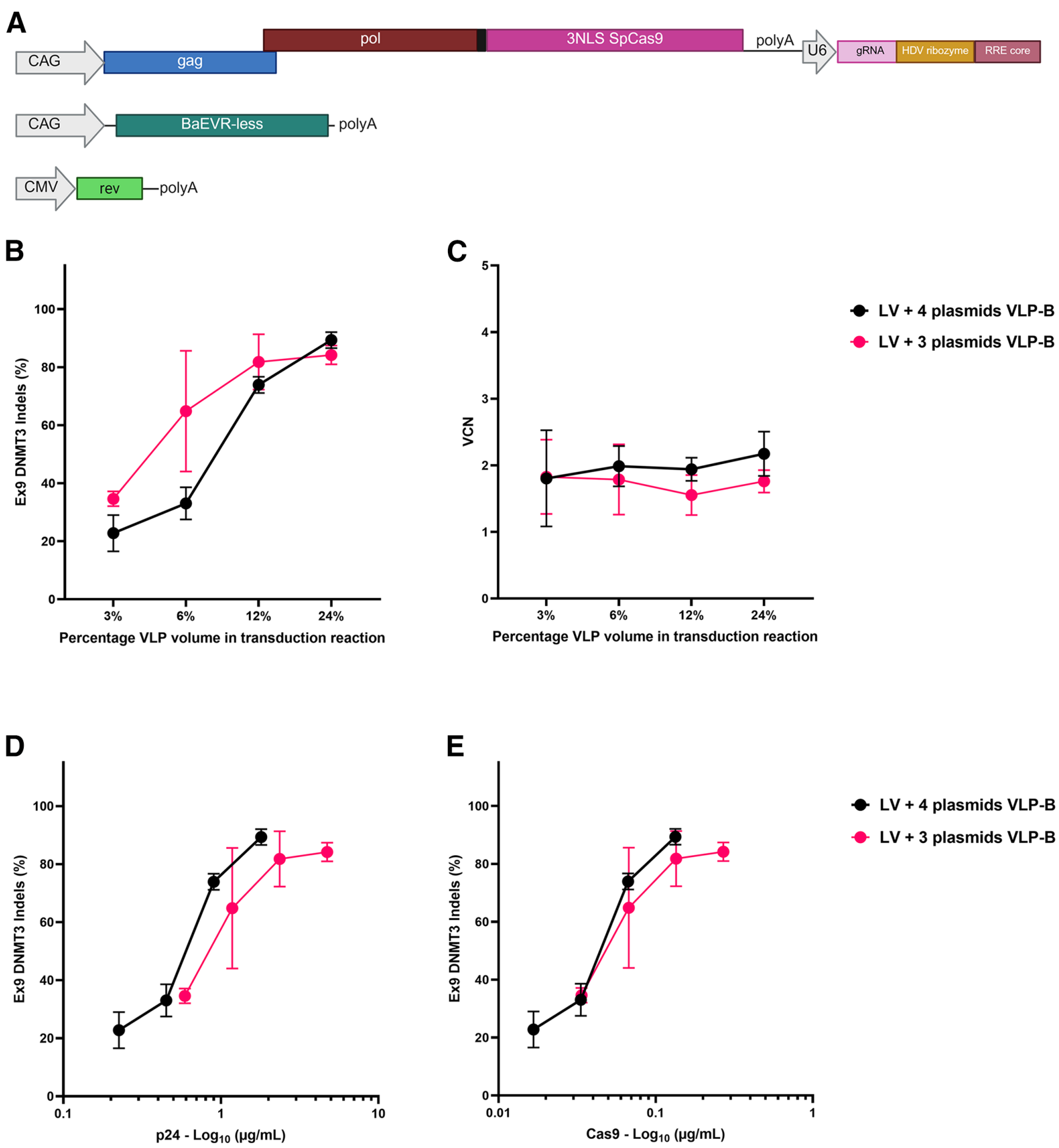

Figure 3. Comparison of Cas9-VLP activity produced using either three or four plasmid systems
Design of three plasmid system used to make Cas9-VLP (A). T cells from three healthy human donors were transduced with an anti-CD19 CAR LV vector and increasing amounts of Cas9-VLP targeting DNMT3A made using either the three or four plasmid system. DNMT3A indel frequency (B, D, E) and VCN (C) were determined seven days post-transduction. *n* = 3. Data are expressed as the mean ± the standard deviation.

Based on these results, we decided to use RetroNectin without preloading because it required the least manipulation and was suitable for both LV and γ-RV transduction.

Optimization of the plasmid design allows the production of VLPs with a three-plasmid system

Having optimized our transduction process, we focused on any improvements that could facilitate the scale-up in a GMP environment. Since VLP production is similar to LV vector production, we focused on finding additional bottlenecks related to costs and safety. In a cGMP setting, when a transient production system is used to produce LV vectors, DNA manufacturing might represent a significant cost in producing the drug substance. Thus, reducing the number of plasmids used during transfection was considered an important cost-saving step. To do this, we moved the sgRNA-expressing cassette onto the same plasmid backbone encoding the HIV-1 Gag-Pol-Cas9 fusion, to reduce the number of transfected plasmids from four to three (Figure 3A). Furthermore, we replaced the ampicillin resistance cassettes on all plasmids with the RNA-OUT antibiotic-free selection cassette to make the plasmids amenable to GMP requirements.

After further optimizing the plasmid ratio used (data not shown), VLPs produced with a four-plasmid system and VLPs produced with the new three-plasmid system were compared for their potency in editing DNMT3A in primary donor T cells co-transduced with anti-CD19 CAR LV. Different quantities of VLP were tested, and a curve of editing activity was obtained for each VLP preparation (Figure 3B). The curves generated were assessed for parallelism. No significant difference in the slope of the curves was observed, indicating that the two production systems were generating biologically equivalent particles and could be used interchangeably for production depending on needs (GMP or optimization/research). Equivalent activity is further demonstrated when comparing indels using p24 or Cas9 content normalization (Figures 3D and 3E). Furthermore, the level of LV transduction was comparable when the two VLP preparations were used (Figure 3C), indicating that the VLP drug substance produced with a three-plasmid system was not negatively impacting the VCN in the cell product.

VLPs can generate edited CAR T cells using a process amenable to cGMP settings

After optimizing transduction conditions, we investigated whether it was possible to produce edited CAR T cells at scale using VLPs following a process amenable to cGMP settings. Furthermore, we compared our VLP-LV co-transduction process to an LV transduction process followed by electroporation of Cas9 RNP. After activation of T cells from three healthy donors using anti-CD3 and anti-CD28-coated flasks, cells were transduced with VSV-G pseudotyped CD19 CAR LV vector alone, or with the same VSV-G pseudotyped LV vector plus BaEV-Rless pseudotyped VLP targeting DNMT3A using g6, or with the same BaEV-Rless pseudotyped VLP plus GaLV pseudotyped γRV vector. One day post-transduction, a portion of the LV vector-only cell sample was subjected to electroporation with RNP complexed with DNMT3A g2 or g6. All cells were then expanded into G-Rex6 multi-well plates (Figure 4A). Non-transduced cells and cells transduced with LV vector but not subjected to RNP electroporation or VLP transduction were used as controls. Prior to expansion, the

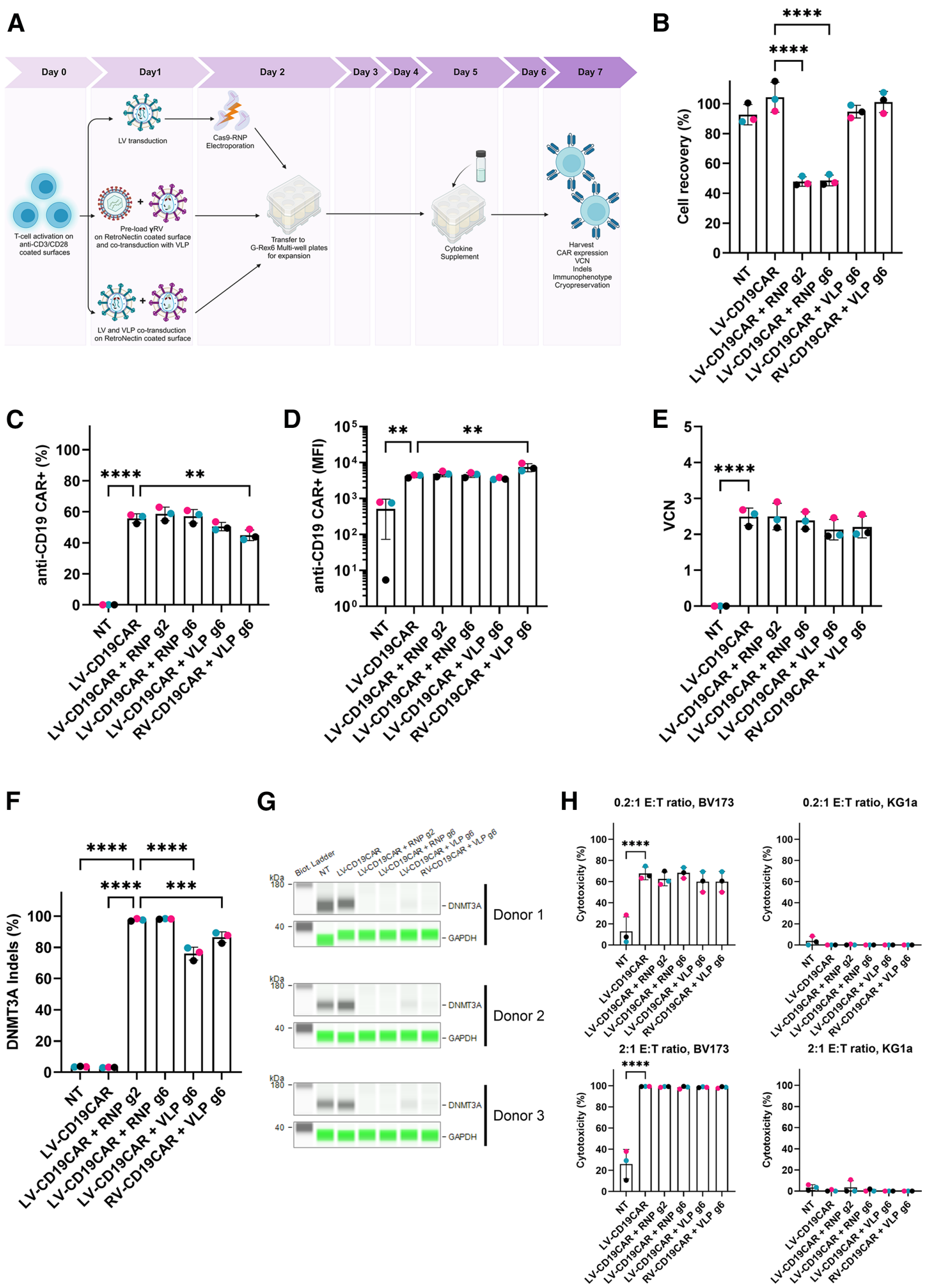

Figure 4. Comparison of the cGMP-compatible processes to generate edited CAR T cells using electroporation or VLP

(A and B) On day 7, samples were collected and assessed for anti-CD19 CAR expression by flow cytometry (C) and mean fluorescence intensity (MFI) (D). Vector copy number was determined by ddPCR (E). DNMT3A indels were assessed by targeted deep sequencing (F). The loss of DNMT3A expression was confirmed by capillary western blotting (G). Edited CAR T cells were evaluated in a killing assay against CD19-positive cells (BV173) and CD19-negative cells (KG1a) at two different ratios of effector: target cells (E:T) (H). One-way ANOVA was used for all the statistical analyses (*$p \leq 0.05$; **$p \leq 0.01$; ***$p \leq 0.001$; ****$p \leq 0.0001$). $n = 3$. Data are expressed as the mean ± the standard deviation.

number of cells recovered after electroporation was significantly lower ($p \leq 0.0001$) compared to controls and VLP-treated cells, ~45% versus ~90%, respectively (Figure 4B). Furthermore, electroporated cells also showed a lower viability (RNPg2 $p = 0.0274$, RNPg6 $p = 0.0486$) than LV transduced cells, ~91% versus ~97%, respectively (Figure S4A). However, 6 days after transduction, we were able to obtain equal fold-cell expansion, indicating that any viable cells recovered from electroporation were able to expand (Figure S4B).

We characterized the final cell product 6 days after transduction. No skewing of the CD4/CD8 ratio was observed in any of the samples analyzed (Figure S4C). The expression of CD19 CAR was evaluated by flow cytometry in the treated sample and controls. All the LV vector transduced samples showed a comparable percentage and level of expression of the CD19 CAR (Figures 4C and 4D). However, a statistical difference was evident when comparing CD19 CAR expression between cells transduced with γRV and LV vectors: cells transduced with γRV vectors had a lower percentage of positive cells ($p = 0.0067$) but a higher mean fluorescence intensity (MFI, $p = 0.0048$). When examining different cell compartments, we observed that, compared to LV-transduced cells, the percentage of CD19 CAR-positive cells was higher in the γRV-transduced cells within the CD8-positive cell compartment (Figure S4D). We attribute these differences to the intrinsic characteristics of γRV transduction, integration patterns, or vector design. The VCNs of the LV or γRV transduced sample were comparable and around 2.5 (Figure 4E). Thus, electroporation or VLP co-transduction did not result in any interference with VCN.

We confirmed the editing of the DNMT3A locus using target deep sequencing (Figure 4F) and verified the knockout of DNMT3A using capillary western blotting (Figure 4G). Delivery of RNP via electroporation was significantly more efficient ($p < 0.0001$) at editing the DNMT3A locus than VLPs (~99% vs. ~70%–80%).

Characterization of the T cell subsets in the final product (Figure S5E) did not show any peculiar skewing. Only an enrichment in the T central memory compartment and a reduction in the T cell naive compartment were observed in T cells transduced with γRV and edited with VLP. Exhaustion markers, PD1 and TIM3 were slightly elevated in transduced cells compared to the non-transduced controls (Figure S5F). Cells transduced with γRV showed a significant increase in CD39 marker expression (Figure S5G, $p = 0.0030$)

and an increased percentage of CD25$^+$ CD69$^+$ (Figure S4H, $p = 0.0005$). This overall indicates that γRV transduction might increase activation and consequently lead to exhaustion in T cells.

Additionally, since aneuploidy was reported following Cas9-RNP treatment of activated T cells, we employed the iCS-digital PSC: aneuploidy test (Stem Genomics) to evaluate whether we could detect loss or gain of a chromosome in our samples. No significant variation in chromosome numbers was observed in the samples analyzed (Figure S6).

The cell products were also evaluated for their specific killing activity at two different effector-to-CD19$^+$ BV173 cell and effector-to-CD19$^-$ KG1a cell ratios (Figure 4H). Cytokine release was also assessed (Figure S7). CAR-expressing cells were able to kill the BV173 cell line but not the KG1a cell line, indicating selectivity and specificity for recognition of CD19 on the cell surface. DNMT3A editing with VLP or RNP did not influence T cell killing activity.

Characterization of Cas9-VLP

We assessed p24 and Cas9 content using a Lumit immunoassay and ELISA, respectively (Table S1). Although the dataset is limited and confounded by the packaging of different sgRNA among the samples analyzed, BaEV-Rless pseudotyped VLP preparations contained on average $\geq$ 2-fold more p24 and Cas9 than VSV-G pseudotyped VLP or LVC preparations; further experiments are necessary to understand the biology of these particles.

To gain additional insight into the particle makeup, we performed capillary western blotting to assess p24 and Cas9 qualitatively (Figure S8A). Interestingly, using the Cas9 antibody, we observed a band at approximately 50 kDa not seen in the Cas9 only control, and we suspect that HIV-1 protease may cleave Cas9 with low efficiency, as previously reported by others.[36] Thus, the quantity of Cas9 detected by ELISA could be misleading if used as a surrogate for activity, as we do not know which epitopes are recognized by the ELISA kit, and likely, non-functional Cas9 may also be detected.

To further characterize the VLP, we extracted and sequenced the VLP total RNA content. After Illumina next-generation sequencing, we aligned the resulting sequences to our reference sequence (Figure S8B). Of the mapped reads, the majority corresponded to the sgRNA scaffold, indicating that the sgRNAs were likely in the proper conformation. Sequences aligned with HDV and RRE were also detected, albeit at a lower percentage, likely indicating successful HDV ribozyme activity in the majority of cases. Interestingly, the RRE sequence was detected at a higher rate than the HDV sequence.

Cas9-VLP has a different off-target profile than traditional RNP electroporation

Since our Cas9-VLP characterization data indicated that we were delivering fewer molecules than traditional RNP electroporation (for 1×10^7 cells, 0.4–0.6 nmol Cas9 using VLPs versus 0.8 nmol using electroporation), we wondered if potential off-target effects were reduced compared to conventional RNP delivery via electroporation. We produced BaEV-Rless pseudotyped VLP preparations using sgRNA sequences directed against TRAC, lymphocyte activation gene 3 (LAG3), and cytotoxic T lymphocyte antigen 4 (CTLA-4), which have been previously shown to exhibit significant off-target activity.[39] We used these VLPs to transduce T cells from three different donors, and in parallel, we also electroporated the same donor cells with RNP complexes containing the same sgRNAs. Then, we analyzed on- and off-targeted CRISPR edit sites with the rhAmpSeq CRISPR Analysis System and the previously described rhAmp Primer Pools.[39] Statistical analysis was conducted to determine whether differences in off-target levels were detected between the two conditions tested, VLP and electroporation. For this purpose, we excluded all loci in which at least one donor was showing a preexisting single-nucleotide polymorphism and identified all loci in which editing activity in the VLP or electroporation group was significantly above 7%. Thus, after data curation, 14 off-target loci were analyzed for the TRAC sgRNA, 62 off-target loci for the LAG3 sgRNA, and 19 off-target loci for CTLA-4. After graphing the data (Figure 5), we performed paired t test with Benjamini-Hochberg procedure to adjust for multiple comparisons and identify any statistically relevant difference between the VLP and RNP electroporation groups. For the TRAC sgRNA, statistical significance was observed in editing for the target locus ($p = 0.0182$) and for 6 off-target loci (Table S2). For LAG3 sgRNA, no statistical difference was identified for on-target or off-target editing (Table S3). For the CTLA-4 sgRNA, statistical significance was observed in editing for the target locus ($p = 0.002$) and for editing of 8 off-target loci (Table S4). To further identify whether certain off-target loci were more prone to be edited by VLP or electroporation, we also graphed the mean of paired differences for these datasets (Figure S9). Overall, the results show that even when statistical significance was not reached, potential differences in off-target editing are possible.

To further compare electroporation and VLP as RNP delivery method, a linear mixed model was also used to assess the overall editing effect while accounting for the correlation of multiple samples within donor and locus. For the TRAC sgRNA, the estimated odds of gene editing with electroporated RNPs are 2 times higher than the odds of editing with VLP ($p < 0.0001$). The estimated means of gene editing are 14.1% (95% confidence interval, CI: 7.5%–24.9%) for electroporation, and 7.5% (95% CI: 3.9%–14.2%), respectively. For LAG3 sgRNA, the result was not statistically significant ($p = 0.0751$). The estimated means of gene editing are 8.1% (95% CI: 6.5%–9.9%) for electroporation, and 7.4% (95% CI: 6%–9.1%), respectively. For the CTLA-4 sgRNA, the estimated odds of gene editing with electroporated RNP are 4.14 times higher than the odds of gene editing with VLP ($p < 0.0001$). The estimated means of gene editing are 21% (95% CI: 13.1%–31.8%) for electroporation, and 6% (95% CI: 3.5%–10.1%), respectively.

DISCUSSION

Recent advances in cancer treatment with CAR T cells have highlighted the need to further enhance the potency of these therapeutic

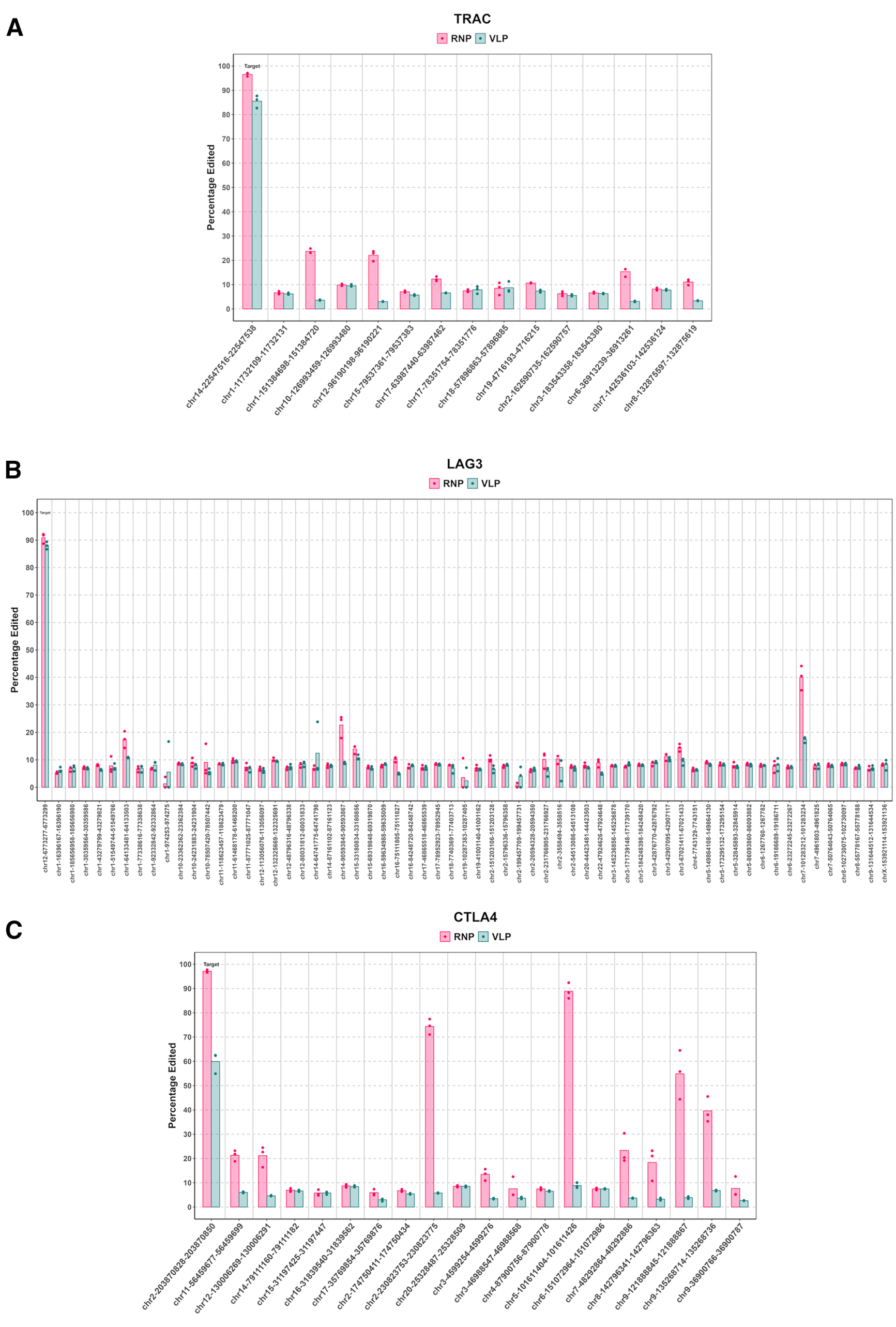

Figure 5. Evaluation of off-target editing of RNP delivered by VLP or by electroporation

T cells from three healthy donors were transduced with Cas9-VLP (VLP group) or subjected to RNP electroporation (RNP group). Three different sgRNA directed against TRAC (A), LAG3 (B), and CTLA-4 (C) were used. To evaluate off-targets, rhAmp seq analysis was performed, and after data curation, the percentage values of gene editing were plotted and compared across all loci that show an average editing above 7% in the VLP or RNP groups. Data are expressed as the mean ± the standard deviation.

tools.[3,8] Gene editing is one strategy proven to improve CAR T cell[4,13] efficacy by increasing cell expansion and persistence *in vivo*. There is also growing interest in using this approach to generate universal off-the-shelf CAR T cells[19] which could significantly expand their use and reduce manufacturing time and costs. Currently, gene-edited CAR T cells are primarily manufactured using electroporation with Cas9-RNP. This method offers multiple advantages, primarily being highly efficient and the ability to deliver components transiently.[40] However, there are also significant drawbacks, such as the high cost and time involved in acquiring GMP-grade Cas9-RNP components, need for specialized equipment, and the loss of cells after electroporation, which may be a significant concern for some patients depending on disease status and prior treatment.[40] Thus, it is unsurprising that researchers have focused on alternative delivery methods, such as cell-penetrating peptides, lipid nanoparticles, and VLPs, which can also deliver CRISPR components in protein or mRNA form.

Virus-like particles offer a promising approach, as they have been studied for years as vaccine candidates.[41] As a result, multiple strategies for the design and manufacture of VLP are well refined and characterized.[42] Various groups have focused on developing VLPs derived from retroviruses and lentiviruses, which have the advantage of leveraging knowledge of production and transduction protocols already applied in gene addition cell and gene therapy.[21,22,26,27,29,32,33,37] Methods for generating HIV-like particles that deliver exogenous proteins are also well described in the literature.[43] In general, these approaches are based on directly fusing the protein of interest, in this case Cas9, to the carboxyl terminus of gag or using additional adapters to mediate co-packaging. However, to allow efficient transduction of target cells, the viral particle must undergo a maturation process through cleavage of the gag polyprotein by the viral protease.[44] This cleavage step can also be exploited to release the protein intracellularly. In both cases, the co-expression of full-length wild-type Gag-Pol during particle production is required to provide protease activity. Instead of using both wild-type Gag-Pol and Gag-Cas9, we employed a different approach: we fused Cas9 directly to the integrase of the Gag-Pol polyprotein to leverage the natural Gag/Gag-Pol self-assembly ability, thus eliminating the need to use two plasmids to express Gag-Pol and Gag-Cas9 or optimize stoichiometry conditions for production, as other investigators have done.[21,22,27–29] A similar approach was previously described by Haldrup et al., but not fully developed.[30] Furthermore, by adding

an HDV ribozyme sequence and the RRE sequence to the 3′ end of the sgRNA, we were able to significantly improve VLP activity (Figure S2), likely through more efficient nuclear export of the sgRNA actively mediated by Rev. As shown by western blot analysis, this approach enabled cleavage of the Gag polyprotein and incorporation of Cas9 into VLPs (Figure S8). More importantly, our particles demonstrated a high editing activity in cell lines and, with optimized transduction conditions, in relevant primary T cells. To further improve our approach, we optimized our production system to reduce the number of plasmids required for functional VLPs to three. This was achieved by inserting the sgRNA-expressing sequence into the same plasmid backbone used to express Gag-Pol-Cas9, as shown in Figure 3. The small number of plasmids required represents a reduction in the costs and complexity associated with VLP production.

In contrast to the production of Cas9 protein and sgRNA, which requires two distinct manufacturing approaches, our production of Cas9-VLP is straightforward. The Cas9-VLPs described here are produced by transfecting cells and employing downstream processes commonly used in the field to generate clinical-grade LV vectors.[45] Specifically, we have produced our Cas9-VLP using 293T cells (SJ293TS and SJ293TS-DPB) adapted to grow in suspension using serum-free media.[46,47] SJ293TS cells have been used by our cGMP facility to produce clinical-grade LV, as well as a clinical AAV vector (unpublished data). All VLPs used in this study were purified by anion-exchange chromatography, concentrated, and formulated in medium using tangential flow filtration or diafiltration steps. Notably, since the Cas9-VLPs do not contain a vector genome, there is no need for time-consuming and expensive replication-competent lentivirus assays. Because the upstream and downstream processes for VLP production are highly similar to the clinical grade LV manufacturing, transfer to a cGMP setting is likely to be facilitated. This contrasts with comparable VLP systems that rely on adherent, serum-dependent cell lines. Moreover, it is likely that the characterization of residual process impurity (such as host cell protein and host cell DNA) using assays already used after current LV manufacturing could be easily applied to VLP.

An important step in optimizing the Cas9-VLP system was identifying which envelope protein to use. Since we were interested in using Cas9-VLP to generate CAR T-edited cells, our first approach was to investigate whether a single particle could deliver both a vector genome expressing CAR and Cas9-RNP. However, we found that this LVC system was associated with additional optimization drawbacks, such as the need to use wild-type Gag-Pol to obtain VCNs greater than 0.5 and was less efficient than anticipated. Furthermore, based on the transduction pattern observed (Figures 2 and S11), the data suggest that LVC are a mixture of LV vector particles, Cas9 VLPs, with a minimal portion of particles containing both the genome and Cas9 RNPs. Thus, we investigated whether a co-transduction approach would be more suitable for generating CAR T-edited cells. We realized that in order to avoid competition for cellular receptors between VSV-G pseudotyped LV vectors encoding CAR and gene-editing VLPs, it was necessary to pseudotype VLPs with a different glycoprotein, in this case, the BaEV glycoprotein lacking the R peptide. We demonstrated that in primary human T cells, BaEV-Rless VLPs exhibited superior transduction and editing efficiency compared to VSV-G VLPs, particularly in the presence of VSV-G pseudotyped LV CAR vector. Using BaEV-Rless VLPs and a VSV-G LV vector under non-optimized transduction conditions, we observed greater than 60% edited cells, with more than half of this population also expressing a CAR, as shown in Figure 2. Under optimized transduction conditions, our VLPs yielded editing percentages above 80% (Figure S4).

To further understand the biological makeup of VLPs, we characterized them using a series of complementary assays (Figure S8; Table S1). However, no single assay proved suitable to confirm the quality of the VLP preparation. Thus, as with any LV and γRV vector preparation, a functional assay on an appropriate cell line is needed to determine the editing efficiency of each VLP preparation. As shown in Figure 3, normalization of VLP activity using p24 and Cas9 content is also possible; however, a challenge we encounter is that ELISA-based assays to evaluate p24 and Cas9 can be time-consuming and often susceptible to significant assay variation or insufficient sensitivity. Furthermore, since commercial kits are primarily for research use and not validated for analyzing complex protein-RNA mixtures such as VLPs, optimizing and developing new assays that provide more insight into particle composition will be essential to establish batch-to-batch release criteria, as well as to evaluate any residual active Cas9-VLP in the final edited cell drug product.

It is well-known that BaEV-Rless LV vectors efficiently transduce T cells due to the upregulation of the receptors ASCT1 and ASCT2 upon activation using cytokines.[48,49] We hypothesize that the high gene editing observed with BaEV-Rless VLP may correlate with a different entry pathway compared with VSV-G VLP and is not only dependent on the higher number of particles delivered, as indicated by the measured amounts of p24 and Cas9 detected in the BaEV-Rless VLP preparations compared to VSV-G VLPs (Table S1). In fact, BaEV-Rless VLPs should fuse directly at the plasma membrane and avoid passage through the endosome, where innate immune sensors (e.g., toll-like receptors, RIG-I, and interferon-induced transmembrane proteins) could block transduction and editing by recognized RNPs or other viral components. Indeed, it has been shown that sgRNA can be recognized by RIG-1 and induce interferon-dependent antiviral responses, thereby rendering cells refractory to gene editing.[50] Further experiments are necessary to confirm the role of the VLP entry pathway in correlation to editing efficiency. For example, when testing LV vector pseudotyped with BaEV-Rless and VLP pseudotyped with VSV-G, which should not interfere with each other for the cellular receptor binding, we cannot detect the same level of editing and VCN (Figure S8), indicating that pseudotyping VLP with BaEV-Rless is essential to high editing. Additionally, when transducing healthy donor T cells with LVC pseudotyped with BaEV-Rless, rather than VSV-G, and using our

optimized transduction protocol (Figure S11), higher editing and CAR expression were achieved. Understanding the mechanisms governing RNP trafficking to the nucleus upon delivery by VLP or LVC could inform strategies to further enhance VLP editing capacity.

We also showed that achieving high editing efficiency in T cells using VLP requires optimizing the method for T cell activation (Figure S3). Having evaluated both the TransAct and the anti-CD3/anti-CD28 antibody-coated surface, we demonstrated that using immobilized antibodies resulted in a higher editing efficiency, likely correlated with the higher expression of the activation markers CD25 and CD69 in T cells 1 day post-activation. This result is not surprising: Arndt et al. previously showed that immobilized antibodies mediate T cell stimulation comparable to that dependent by T cell receptor signaling, whereas antibodies cross-linked in solution result in transient activation.[51] Furthermore, T cell activation has been reported to be essential for achieving high editing efficiency, even at the cost of off-target effects such as aneuploidy.[23] However, in our hands (Figure S6), no chromosome loss was detected.

Additionally, we showed that using RetroNectin as a transduction enhancer significantly increased transduction and editing efficiency, independent of the pseudotyped VLP, LV, or RV vector used (Figure S3). Likely, the ability of RetroNectin to bring viral particles and cells into proximity with each other provides an additional chance for cell receptor clustering with viral envelope proteins, resulting in a superior transduction enhancer effect compared with that provided by the reduction of electrostatic membrane repulsion mediated by LentiBOOST. It is also possible that, when pseudotyped envelope proteins use the cell membrane as an entry pathway (e.g., BaEV-Rless and GaLV), changes in the cell membrane charge negatively affect fusion.

Unfortunately, the use of both antibody-coating for T cell activation and RetroNectin hinders scale-up in the production of the cellular product. In fact, plastic surfaces demonstrate additional challenges in scale-up and require additional manual handling.[52] Further optimization with special microbeads that allow protein binding and testing different transduction enhancers, such as Vectorfusin may be necessary to adapt our transduction protocol to single-process setups, such as the CliniMACS Prodigy, which could enable automation and bedside therapy generation.[53] An additional option to improve scale-up and avoid the use of exogenous RetroNectin is co-display of the molecule on the VLP envelope surface, as recently described by Botchkarev et al.[34]

By directly comparing different delivery strategies, we have shown that RNP delivery via VLPs is feasible and may offer greater flexibility in experimental design than RNPs delivery using electroporation. By varying the VLP volume during transduction, we have demonstrated the percentage of edited cells in a given drug product can be readily adjusted. This "dialing in" ability may help in understanding how CAR T cell editing confers a persistence advantage *in vivo*. Furthermore, we have shown that VLPs deliver fewer RNPs than electroporation and, consequently, we observe a trend of reduced off-target editing relative to electroporation, thereby improving the product's safety profile. However, due to the low-statistical power inherent to this study, additional experiments are necessary to study off-target effects of VLPs compared to electroporation.

We envision that a single batch of clinical-grade VLPs could complement multiple cell therapies regardless of viral vector or expression cassette. Our Cas9-VLPs are pseudotyped with the BaEV-Rless envelope protein and do not interfere with the delivery of CAR-expressing LV or γ-RV vectors, often pseudotyped with either VSV-G or GaLV. By developing a set of VLPs that target different host genes, a "toolbox" from which investigators can select appropriate Cas9-VLPs to complement their therapeutic needs could be established.

Additionally, given interest in developing CAR T cells with multiple edits,[20,54] we were able to develop VLPs that can deliver adenosine base editors (data not shown) and are optimizing cytosine base editor-VLP. We are also further developing a system to produce VLPs that can simultaneously deliver multiple sgRNAs targeting different loci.

Overall, we have demonstrated that VLPs can be efficiently co-delivered with LV or γRV to generate edited CAR T cells, offering a significant advantage with respect to cell viability and recovery compared to electroporation. VLP production is scalable and offers a flexible, cost-effective alternative to electroporation for modifying CAR T cells, potentially enabling them to reach patients more quickly and achieve greater clinical benefit.

MATERIALS AND METHODS

Cloning of HIV-1 Gag-Pol-Cas9 construct

The *Streptococcus pyogenes* Cas9 sequence with 3NLSs[55] and the HIV-1 matrix and capsid cleavage site (QVSQNY↑PIVQNI) at the 5′ end was human codon-optimized using GenSmart Codon Optimization tool (GenScript, Piscataway, NJ, USA), before being divided into two overlapping fragments and synthesized as gBlocks HiFi Gene Fragments (Integrated DNA Technologies, Coralville, IA, USA). The HIV-1 Gag-Pol-Cas9 construct, CAGG-HIVgpco-Cas9co, was obtained using Gibson Assembly: after linearization of CAGG-HIVgpco-Cas9co by FseI and HindIII pCAGG-HIVgpco (codon-optimized Gag-Pol), the plasmid was mixed with a polymerase chain reaction fragment encoding the 3′ of integrase from pCAGG-HIVgpco, with the two previously mentioned fragments encoding Cas9, and with the fragment encoding the rabbit beta-globin polyadenylation signal (from pCAGG-HIVgpco).

Cloning of sgRNA constructs

To obtain the sgRNA encoding plasmids, a fragment containing a sgRNA directed against GFP with the "flip and extension" (F + E) sgRNA scaffold,[38] the HDV ribozyme, and the RRE was synthesized by GenScript and cloned using *DraIII* and *HindIII* into a BPK1520 (from Keith Joung, Addgene # 65777) plasmid previously cloned to express the same sgRNA to obtain the plasmid

GFP.gRNAx.HDV.RRE.core. All the other sgRNA-encoding plasmids were obtained from the GFP.gRNAx.HDV.RRE.core plasmid by mutagenesis with the Q5 Site-Directed Mutagenesis Kit (New England Biolabs) according to the manufacturer's instructions using primers binding the sgRNA scaffold (GTTTAAGAGCTATGCTGGAAAC) and an sgRNA-specific primers (reported in Table S5). The B2M, TRAC g3, DNTM3A g2, sequences were previously validated elsewhere.[14,56,57] Additional DNMT3A sequences were designed and selected with the CHOPCHOP web tool.[58] Plasmids to express TRAC, LAG3, and CTLA-4 sgRNA associated with high off-target activity were synthesized by GenScript.

Retroviral and LV transfer plasmids

The third-generation LV vector expressing mCherry under MND promoter, pSJL12 MND-mCherry, was used in optimization experiments to produce the LVC particles. pSJL12 MND-mCherry was obtained by exchanging the mCherry gene with the GFP cassette of pSJL12 MND-GFP, which was derived from pCL40-MND-GFP-AF[46] by adding back to the plasmid backbone the beta-lactamase gene flanked by *BsmBI* sites to allow fast conversion between an ampicillin resistance plasmid and RNA-OUT.

pSJL643-CD20.CD123CAR.CD28HTM.CD28Z-AF is the third-generation LV vector plasmid encoding the anti-CD123 CAR currently used in the CD123-Directed Autologous T cell Therapy for Acute Myelogenous Leukemia (CATCHAML) clinical trial at St. Jude Children's Research Hospital (NCT04318678).[59]

pSJL651-4L-B7H3CAR-AF is the third-generation LV vector plasmid currently used for the production of CAR T in the B7-H3-Specific Chimeric Antigen Receptor Autologous T cell Therapy for Pediatric Patients with Solid Tumors (3CAR) clinical trial (NCT04897321).[60]

The bispecific CD19/CD22 third-generation LV vector, which is going to be used in the upcoming clinical trial NCT06777979, was also used in some experiments.

pCFG-aCD19CAR, was generated by cloning the anti-CD19 CAR excised from pMSCV-antiCD19BBz[61] using *EcoRI* and *HincII* enzymes into *EcoRI* site and *NotI* site of pCFG-GFP, a non-self-inactivating gamma retroviral vector under CMV promoter; the *NotI* site was rendered compatible with *HincII* by using the Quick Blunting Kit (New England Biolabs, Ipswich, MA, USA).

pCL45-MND-CD19CAR was obtained by converting the second-generation vector pCL20-MND-CD19CAR into a third-generation vector using part of the pCL40-MND backbone.

Cell lines

HOS-mCat1 cells were derived from HOS cells (American Type Culture Collection, ATCC CLR-1543) after limiting dilution and transduction of a fourth-generation LV vector[62] encoding for the murine Slc7a1 protein under MND promoter to confer susceptibility to ecotropic vectors. Cells were maintained adherent in Dulbecco's modified Eagle medium (Corning Life Sciences, Corning, NY, USA) supplemented with 10% fetal bovine serum (FBS) (Atlanta Biologicals, Flowery Branch, GA, USA) and 2 mM L-alanyl-L-glutamine (Corning Life Sciences) at 37°C with 5% CO_2 (D10).

SJ293TS cells are a serum-free suspension cell line derived from HEK293T previously described by Bauler et al.[46]; SJ293TS-DPB cells were derived from SJ293TS after protein kinase R, and B2M were knocked out using CRISPR-Cas9.[47] Both suspension cell lines were maintained at 37°C, 8% CO_2, and 125 rpm.

BV173.ffLuc and KG1a.ffLuc were modified to express firefly luciferase from BV173 and KG1a, respectively. Both cells were cultured in RPMI1640 medium (Cytiva HyClone, Marlborough, MA, USA) supplemented with 10% FBS (Avantor Seradigm, Center Valley, PA) and 2 mM GlutaMAX (Gibco, Thermo Fisher Scientific, Waltham, MA, USA) at 37°C with 5% CO_2.

Production of LP Cas9 and Cas9-RNP VLPs

LV vectors, LVC, and Cas9-VLP were produced using SJ293TS or SJ293TS-DPB with variations to the Bauler et al. protocol.[46,47] Briefly, 50 mL culture SJ293TS or SJ293TS-DPB cells were seeded at 2×10^6 cells/mL for same-day transfection or 1×10^6 cells/mL for next-day transfection in 80% FreeStyle 293 Expression medium and 20% LV-MAX media (Thermo Fisher Scientific, Waltham, MA, USA). For the production of LV vectors, 35 μg of the transfer vector and 10 μg of pCAG-kGP1-1R-AF (*gag-pol*) were used in the transfection mixture; in contrast, 20 μg of the transfer vector, 25 μg of CAGG-HIVgpco-Cas9co, 0.325–2.5 μg of pCAGG-HIVgpco-AF (codon optimized *gag-pol* in the RNA-OUT plasmid backbone), and 20 μg of sgRNA expressing constructs were used to produce LVC. For VLP, 45 μg CAGG-HIVgpco-Cas9co and 20 μg of sgRNA-expressing constructs or 55 μg of the all-in-one HIV-1 Gag-Pol-Cas9-sgRNA plasmid were utilized. Additional helper plasmids were held constant between viral particle preparations: 5 μg of pCAG-VSV-G-AF or pCAG-BaEV-R-less-AF, and 0.625 μg pCMV-*rev*-AF. A ratio of 2:1 PEIpro (Polyplus Transfection, Strasbourg, France) to total DNA in PBS was maintained for all transfections. The transfection mixture was scaled down or up proportionally according to the culture volume. For initial optimization experiments in which different plasmid quantities were evaluated, 20 mL cultures were used. Twenty-four hours post-transfection, if the preparation was going to be further purified, the culture was diluted with an equal volume of FreeStyle 293 Expression medium containing Benzonase to obtain a final concentration of 6.25 U/mL. Vector or VLP-containing supernatants were collected 48 h post-transfection after removing cells using centrifugation at 330 ×*g* and filtered using 0.22 μm polyethersulfone membrane filters before being aliquoted or purified using XT Acrodisc Ion Exchange unit with Mustang Q membrane (Pall Life Science, Port Washington, NY, USA) per manufacturer's instruction and as previously described elsewhere.[46] Briefly, ÄKTA Avant chromatography system

was used to load the viral supernatant adjusted to 300 mM NaCl with 50 mM Tris (pH 8.0) on the 0.86 mL Acrodisc capsule. A wash step consisting of ten column volumes of 300 mM NaCl with 50 mM Tris (pH 8.0) was also included. Elution was performed manually using 1.2 mL of 2 M NaCl and 50 mM Tris (pH 8.0). Eluted particles were subjected to desalting using disposable Sephadex G-25 resin PD 10 Columns (Cytiva, Marlborough, MA, USA) and diluted with an equal volume of X-VIVO 15 Serum-Free Hematopoietic Cell Medium (Lonza, Basel, Switzerland). For processing of 1 L volumes, 5 mL Mustang Q XT5 ion-exchange capsules were used for the anion-exchange chromatography step. Diafiltration was performed with Vivaflow 50 with a 100,000 molecular weight cutoff (Sartorius AG, Göttingen, Germany) to X-VIVO 15 media as previously described.[46] Concentrated and purified particles were filtered at 0.22 μm before being aliquoted and stored at −80°C.

Titration, VCN determination, and targeted deep sequencing

LVs, LVC, and VLP were serially diluted in D10 and added to 1.6×10^4 HOS-mCat1 cells, which are predominantly diploid, in the presence of 8 μg/mL Polybrene (MilliporeSigma, Burlington, MA, USA). Twenty-four hours post-transduction, media was added. Four days post-transduction, the medium was removed, cells were lysed with 10 mM Tris pH 8.0, 2 mM EDTA pH 8.0, 0.2% Triton X-100, 200 μg/mL proteinase K, and transferred to a 96-well PCR plate and incubated for 6 min at 65°C and 2 min at 98°C to obtain PCR-ready genomic DNA. For particles containing a vector genome, cell genomic DNA was subjected to digital droplet PCR (ddPCR) using primer-probe sets against the HIV-1 *psi* packaging sequence (or MLV packaging sequence for γRV vectors) and host *RPP30* to evaluate VCN.[46] Assuming two copies of *RPP30* per cell, VCN and titers were determined by calculating the number of copies of HIV-1 *psi* to every two copies of *RPP30* and, if necessary, multiplying by the number of cells transduced and the dilution factor. LV vector titers were reported as transduction units per mL (TU/mL) as described previously.[46]

Genomic DNA of HOS cells transduced with particles containing Cas9 and sgRNA was subjected to targeted deep sequencing by next-generation sequencing, followed by analysis with CRIS.py by the Center for Advanced Genome Engineering at St. Jude Children's Research Hospital.[63] Indels percentages between 40% and 5% were used to calculate gene editing titers by multiplying the percentage of indels and the dilution factor, and expressed as GEU/mL.

Detection of HIV-1 p24 and Cas9 in viral particles

The Lumit p24 Immunoassay was used to measure VLP p24 content and physical titer following the manufacturer's instructions.

To evaluate the amount of Cas9 present in the VLP preparation, after lysis of the particles with Triton X-100 in PBS and 2-chloroacetamide (RETRO-TEK HIV-1 p24 Antigen ELISA Lysing Buffer, ZeptoMetrix, Buffalo, NY, USA), a Cas9 ELISA Kit (Cell Biolabs Inc, San Diego, CA, USA) was used following the manufacturer's instructions with a slight modification: the standard curve was generated using a 3NLS-Cas9 protein produced by Children's GMP, LLC instead of the provided Cas9, which was instead used as positive control.

To evaluate the Cas9 and p24 content of VLPs, qualitative western blots were performed using the Jess Automated Western Blot system (ProteinSimple, San Jose, CA, USA). Briefly, particles containing 330 ng of p24, previously suspended in X-VIVO15 media and 1 *X* Halt Protease Inhibitor Cocktail (Thermo Fisher Scientific) and denatured after the addition of 1.1% SDS and incubation at 90°C for 10 min, were loaded on a 12–230 kDa cartridge (Protein Simple) after the addition of the EZ standard Fluorescent Master mix containing 200 mM DTT solution (ProteinSimple). Streptavidin-NIR ready-made solution (Protein Simple) was used to detect the EZ biotinylated ladder. Primary antibodies HIV-1 Gag p24 Antibody (MAB73602, R&D Systems Inc, Minneapolis, MN, USA) and Guide-it Cas9 Polyclonal Antibody (632607, Takara Bio USA Inc., San Jose, CA, USA) were diluted into Milk-free antibody diluent (ProteinSimple) for a final concentration of 2 μg/mL and a 1:20 dilution, respectively. The 20*X* Anti-Mouse Secondary NIR Antibody (ProteinSimple) and the 20*X* Anti-Rabbit Secondary NIR Antibody (ProteinSimple) were diluted in the Milk-free antibody diluent.

Single guide RNA sequencing

RNA from VLP was extracted using the Maxwell RSC Viral Total Nucleic Acid Purification Kit (Promega, Madison, WI, USA) and the Maxwell RSC Instrument (Promega). Library preparation for Illumina sequencing was performed at the St. Jude Children's Research Hospital Hartwell Center for Bioinformatics & Biotechnology using the NEXTFLEX Small RNA-Seq Kit v.4 (Revvity, Waltham, MA, USA). Sequencing was performed on a NovaSeq platform (Illumina Inc., San Diego, CA, USA), yielding 100 bp paired-end reads.

Sequencing FASTQ file results were analyzed using FastQC (v.0.11.5) and Cutadapt (v.4.4) to remove adapters before proceeding with alignment to the reference sequence with minimap2 (v.2.26). Samtools (v.1.7) and bedtools (v.2.31.0) were used to extract mapped reads and to evaluate coverage and percentage identity, in conjunction with a Python (v.3.9.9) script, basepercentage.py. The script "graph_for_sequencing.R" was used with R (v.4.4.0) to generate the final image. All the bioinformatics analyses were performed using the St. Jude Children's Research Hospital High Performance Computing (HPC) facility.

T cell transduction

Healthy donor-derived $CD4^+/CD8^+$ T cells were purified using the CliniMACS system (Miltenyi Biotec, Bergisch Gladbach, Germany) after labeling human peripheral blood Leukopak (StemExpress) using CD4/CD8 microbeads. T cells were then in 20% FBS and 10% DMSO in X-VIVO15 medium using CryoMed freezer with Profile #1 program at 2×10^7 cells per aliquot. When used experimentally, T cells were thawed and suspended in X-VIVO 15 media supplemented with 5% human AB serum (Valley Biomedicals, Winchester,

VA, USA) and 10 ng/mL of recombinant human IL-7 and IL-15 (Miltenyi Biotec) (X-VIVO 15, 5% human AB serum/IL-7/IL-15, hereafter). Cells were activated using 1*X* T cell TransAct (100*X* stock, Miltenyi Biotec) or by incubation on anti-CD3/anti-CD28 (Takara Bio, San Jose, CA, USA) coated plates or flasks on the day of thawing. Twenty-four hours after thaw, cells were transduced with LV, LVC, and VLP in a total volume of 100 μL and cell density of 2×10^6 cells/mL and in the presence of 8 μg/mL Protamine Sulfate (Fresenius Kabi, Lake Zurich, IL, USA) and when specified 1 mg/mL LentiBOOST (SIRION Biotech, Waltham, MA, USA). When RetroNectin was used as transduction enhancer cells were placed into wells of plates precoated with 7 μg/mL RetroNectin (Takara Bio) in PBS. One day post-transduction, cells were washed and suspended X-VIVO 15, 5% human AB serum/IL-7/IL-15 and expanded until further analysis.

For scale-up experiments, 2×10^6 healthy donor T cells were used for transduction. The same protocol was followed, but cells were placed into a well of a G-Rex 6 M culture plate (Wilson Wolf Manufacturing, St Paul, MN, USA) in 100 mL of X-VIVO 15, 5% human AB serum/IL-7/IL-15 after wash and suspension following transduction.

On day six post-transduction, cells were collected by centrifugation at 400× *g*. Cell staining followed by flow cytometry was used to evaluate the residual expression of protein localized at the cell surface. For DNMT3A, whole cell lysates were subjected to western blot analysis to confirm knockout. VCN analysis and indels measurement using targeted deep sequencing were performed to evaluate vector integration and genome editing, respectively. For aneuploidy analysis, genomic DNA extracted using the Quick-DNA Miniprep kit (Zymo Research, Irvine, CA, USA) was sent to Stem Genomics (Grabels, France) for iCS-digital PSC: aneuploidy test.

Cas9 ribonucleoprotein electroporation of T cells

For electroporation controls, after cell activation and transduction with LV, 1×10^7 cells per condition were prepared in electroporation buffer at 1×10^8 cells/mL before adding an equal volume of RNP mix to have a final concentration of 4 μM research-grade SpyFiCas9 (Aldevron, Madison, WI, USA) and 8 μM of synthetic guide RNA. Cells/RNP mix were electroporated using a MaxCyte GTX electroporator with an OC100× 2 static electroporation processing assembly (MaxCyte, Rockville, MD, USA), and the Expanded T cell 1 pre-optimized transfection protocol. Cells were initially incubated in 6-well plates for 20 min and then transferred and expanded into G-Rex 6 M culture plates.

Flow cytometry

Transduced T cells were stained for flow cytometry analysis to evaluate CAR expression, knockout of targeted genes, and CD4/CD8 expression. Briefly, 1×10^6 cells per sample were collected by centrifugation at 400× g and stained for 30 min at 4°C in autoMACS Running Buffer 15 (Miltenyi Biotec) with Allophycocyanin-Cyanine7 (APC-Cy7) Mouse Anti-Human CD4 (Clone RPA-T4, RUO, BD Biosciences, Franklin Lakes, NJ, USA), Brilliant Violet 510 (BV510) Mouse Anti-Human CD8 (Clone SK1, RUO, BD Biosciences) and the other relevant experiment specific antibodies. The poly-Glycine-Serine (G4S) Linker Rabbit monoclonal antibody Phycoerythrin (PE) Conjugate (clone E7O2V, Cell Signaling Technology, Danvers, MA, USA) was used to detect the G4S linker present in all the CARs single chain fragment variable (scFv). The APC anti-human TCR α/β antibody (Clone IP26, BioLegend, San Diego, CA, USA) or the PE-Cy7 anti-human β2-microglobulin antibody (Clone 2M2, BioLegend) were used to detect knockout of TRAC and B2M, respectively. NucBlue Fixed Cell ReadyProbes Reagent (DAPI, Thermo Fisher Scientific) was used to distinguish between live and dead cells. Samples were analyzed with Attune NxT Flow Cytometer with blue (488 nm), red (638 nm), violet (405 nm), and yellow (561 nm) lasers configuration using VL1 (440/50, DAPI), VL2 (512/25, BV510), YL1 (585/16, PE), YL4 (780/60, PE-Cy7), RL1 (670/14, APC) and RL3 (780/60, APC-Cy7) channels. Ten thousand live single events were collected for each sample.

For scale-up experiments, cells were stained to check for CD4, CD8, CD45RO, CCR7, CD39, PD1, and TIM3 expression, and CD19 CAR Detection Reagent, followed by Biotin-APC antibody, was used to stain CAR-positive cells. Antibodies used are reported in Table S6. BD Pharmingen DAPI Solution (BD Biosciences) was used for viability staining. The CytoFlex Flow cytometer (Beckman Coulter) was used for the analysis.

Data analysis and representation were performed using FlowJo v.10.8 (FlowJo, Ashland, OR, USA).

Capillary western blot to detect DNMT3A

The Jess automated western blot system (Protein Simple) was used according to the manufacturer's instructions to detect DNMT3A and GAPDH in cell lysates. Briefly, 3 million cells were lysed using RIPA buffer, and 3 μg of cell lysate were loaded on a 12–230 kDa cartridge (ProteinSimple) after the addition of the EZ standard Fluorescent Master mix containing 200 mM DTT solution (ProteinSimple). The EZ biotinylated ladder was used and detected with streptavidin-HRP ready-made solution (ProteinSimple). The antibody diluent 2 (Protein Simple) was used during the blocking step and for dilution of primary and secondary antibodies. A 1:250 dilution of Rabbit Polyclonal anti-DNMT3A antibody (GTX129125, GeneTex, Irvine, CA, USA) and a 1:50 dilution of anti-GAPDH monoclonal Antibody (0411, Santa Cruz Biotechnology, Dallas, TX, USA) were used, respectively, for the detection of the proteins in the cell lysates. The Anti-Rabbit HRP Conjugate (ProteinSimple) and the Anti-Mouse Secondary NIR Antibody (ProteinSimple) were used as secondary antibodies at 1:20 dilution.

rhAmpSeq off-target analysis

For rhAmpSeq, T cells were transduced as previously described. In parallel, T cells were also electroporated at day 2 with 3NLS-Cas9 complexed with synthetic sgRNA (Synthego Corporation, Redwood

City, CA, USA) as previously described, but using a R50× 3 static electroporation processing assembly. On day 6 post-transduction or day 5 post-electroporation, cells were collected, and DNA was extracted using the Maxwell RSC Cultured Cells DNA Kit and the Maxwell RSC (Promega, Madison, WI, USA).

Dr. Shengdar Q. Tsai (St. Jude Children's Research Hospital) kindly provided RhAmpSeq CRISPR Panels previously described by Lanzarotto et al.[39] Amplicons were generated using the rhAmpSeq CRISPR Library Kit (Integrated DNA Technologies, Coralville, IA, USA) according to the manufacturer's instructions. Amplicons were pooled, cleaned, and sequenced using the NovaSeq 6000 System (Illumina, San Diego, CA, USA) by the Hartwell Center for Bioinformatics & Biotechnology to obtain at least 90 million 150 bp paired-end reads.

Sequences were then analyzed using rhAmpSeq CRISPR Data Analysis Tool (Integrated DNA Technologies) according to the manufacturer's instructions.

Statistical analysis

GraphPad Prism 9 (GraphPad Software, San Diego, CA, USA) was employed for basic analyses (ANOVA) with Dunnett's multiple-comparison test when necessary, and graph design. Additional statistical analysis and figures for evaluating off-targets effect was performed using SAS 9.4 and R (v.4.3.1). As an initial descriptive summary, the means of paired differences and range for VLP versus electroporated RNP were reported across loci to show the magnitude and variability of differences between VLP and RNP. After the illustration, the percentage values of gene editing were logit-transformed following a small-offset correction to accommodate observations at or near 0% or 100%. Paired *t* tests were performed to compare VLP versus RNP for each on-/off-target locus. Raw *p* values were adjusted for multiple comparisons using the Benjamini-Hochberg false discovery rate procedure. To account for the correlation among multiple samples within each donor and locus, a linear mixed-effects model was used to estimate the overall difference in logit-transformed values between VLP and RNP. Corresponding 95% CIs are reported. All *p* values are two-sided, with statistical significance defined at the 0.05 level unless otherwise specified.

DATA AND CODE AVAILABILITY

- Sequence files for all plasmids used in this study are available upon request.
- Bioinformatic code and scripts for the analysis are available upon request.

ACKNOWLEDGMENTS

This study was supported with funding from the American Lebanese Syrian Associated Charities. Graphical abstract and graphical figures were created with BioRender.com. The authors would like to thank the Dr. Shengdar Tsai's laboratory for providing reagents for rhAmp seq library preparation, and St. Jude Children's Research Hospital shared resources, Hartwell Center for Bioinformatics & Biotechnology and the Center for Advanced Genome Engineering (CAGE), for assisting with rhAmp seq library sequencing, sgRNA sequencing, and indels evaluation, respectively.

AUTHOR CONTRIBUTIONS

F.F., conceptualization, methodology, software, formal analysis, investigation, visualization, and writing – original draft; M.M.W., conceptualization, methodology, and investigation; J.J.P., conceptualization, methodology, formal analysis, and investigation; M.B., C.W., N.M., B.R.L., and J.A.B., investigation; Y.S. and L.T., formal analysis; D.M.L., methodology and resources, S.Z., conceptualization and resources; R.E.T., conceptualization, resources, writing – review and editing, supervision, and project administration. All authors have read and approved the final manuscript.

DECLARATION OF INTERESTS

F.F., M.M.W., S.Z., and R.E.T. have filed patent applications related to cell and gene therapy products. No other competing financial interests exist.

SUPPLEMENTAL INFORMATION

Supplemental information can be found online at https://doi.org/10.1016/j.omta.2026.201809.

REFERENCES

1. Cappell, K.M., and Kochenderfer, J.N. (2023). Long-term outcomes following CAR T cell therapy: what we know so far. Nat. Rev. Clin. Oncol. *20*, 359–371. https://doi.org/10.1038/s41571-023-00754-1.
2. Ogusuku, I.E.Y., Knelangen, N., Engels, B., Subklewe, M., and Lock, D. (2026). Mapping the clinical landscape of multifunctional CAR T cells: targets, trends, and synergies. Mol. Ther. https://doi.org/10.1016/j.ymthe.2026.02.006.
3. Shah, N.N., and Fry, T.J. (2019). Mechanisms of resistance to CAR T cell therapy. Nat. Rev. Clin. Oncol. *16*, 372–385. https://doi.org/10.1038/s41571-019-0184-6.
4. Dimitri, A., Herbst, F., and Fraietta, J.A. (2022). Engineering the next-generation of CAR T-cells with CRISPR-Cas9 gene editing. Mol. Cancer *21*, 78. https://doi.org/10.1186/s12943-022-01559-z.
5. Ceja, M.A., Khericha, M., Harris, C.M., Puig-Saus, C., and Chen, Y.Y. (2024). CAR-T cell manufacturing: Major process parameters and next-generation strategies. J. Exp. Med. *221*, e20230903. https://doi.org/10.1084/jem.20230903.
6. Roybal, K.T., Rupp, L.J., Morsut, L., Walker, W.J., McNally, K.A., Park, J.S., and Lim, W.A. (2016). Precision Tumor Recognition by T Cells With Combinatorial Antigen-Sensing Circuits. Cell *164*, 770–779. https://doi.org/10.1016/j.cell.2016.01.011.
7. Mestermann, K., Giavridis, T., Weber, J., Rydzek, J., Frenz, S., Nerreter, T., Mades, A., Sadelain, M., Einsele, H., and Hudecek, M. (2019). The tyrosine kinase inhibitor dasatinib acts as a pharmacologic on/off switch for CAR T cells. Sci. Transl. Med. *11*, eaau5907. https://doi.org/10.1126/scitranslmed.aau5907.
8. Rafiq, S., Hackett, C.S., and Brentjens, R.J. (2020). Engineering strategies to overcome the current roadblocks in CAR T cell therapy. Nat. Rev. Clin. Oncol. *17*, 147–167. https://www.nature.com/articles/s41571-019-0297-y.
9. Roybal, K.T., Williams, J.Z., Morsut, L., Rupp, L.J., Kolinko, I., Choe, J.H., Walker, W.J., McNally, K.A., and Lim, W.A. (2016). Engineering T Cells with Customized Therapeutic Response Programs Using Synthetic Notch Receptors. Cell *167*, 419–432.e16. https://doi.org/10.1016/j.cell.2016.09.011.
10. Zimmermann, K., Kuehle, J., Dragon, A.C., Galla, M., Kloth, C., Rudek, L.S., Sandalcioglu, I.E., Neyazi, B., Moritz, T., Meyer, J., et al. (2020). Design and Characterization of an "All-in-One" Lentiviral Vector System Combining Constitutive Anti-GD2 CAR Expression and Inducible Cytokines. Cancers *12*, 375. https://doi.org/10.3390/cancers12020375.
11. Labanieh, L., and Mackall, C.L. (2023). CAR immune cells: design principles, resistance and the next generation. Nature *614*, 635–648. https://doi.org/10.1038/s41586-023-05707-3.
12. Ramachandran, M., Dimberg, A., and Essand, M. (2017). The cancer-immunity cycle as rational design for synthetic cancer drugs: Novel DC vaccines and CAR T-cells. Semin. Cancer Biol. *45*, 23–35. https://doi.org/10.1016/j.semcancer.2017.02.010.
13. Bonini, C., Chapuis, A.G., Hudecek, M., Guedan, S., Magnani, C., and Qasim, W. (2023). Genome Editing in Engineered T Cells for Cancer Immunotherapy. Hum. Gene Ther. *34*, 853–869. https://doi.org/10.1089/hum.2023.128.
14. Prinzing, B., Zebley, C.C., Petersen, C.T., Fan, Y., Anido, A.A., Yi, Z., Nguyen, P., Houke, H., Bell, M., Haydar, D., et al. (2021). Deleting DNMT3A in CAR T cells prevents exhaustion and enhances antitumor activity. Sci. Transl. Med. *13*, eabh0272. https://doi.org/10.1126/scitranslmed.abh0272.

15. Gomes-Silva, D., Srinivasan, M., Sharma, S., Lee, C.M., Wagner, D.L., Davis, T.H., Rouce, R.H., Bao, G., Brenner, M.K., and Mamonkin, M. (2017). CD7-edited T cells expressing a CD7-specific CAR for the therapy of T-cell malignancies. Blood *130*, 285–296. https://doi.org/10.1182/blood-2017-01-761320.

16. Chiesa, R., Georgiadis, C., Syed, F., Zhan, H., Etuk, A., Gkazi, S.A., Preece, R., Ottaviano, G., Braybrook, T., Chu, J., et al. (2023). Base-Edited CAR7 T Cells for Relapsed T-Cell Acute Lymphoblastic Leukemia. N. Engl. J. Med. *389*, 899–910. https://doi.org/10.1056/nejmoa2300709.

17. Cooper, M.L., Choi, J., Staser, K., Ritchey, J.K., Devenport, J.M., Eckardt, K., Rettig, M.P., Wang, B., Eissenberg, L.G., Ghobadi, A., et al. (2018). An "off-the-shelf" fratricide-resistant CAR-T for the treatment of T cell hematologic malignancies. Leukemia *32*, 1970–1983. https://doi.org/10.1038/s41375-018-0065-5.

18. Liu, J., Zhang, Y., Guo, R., Zhao, Y., Sun, R., Guo, S., Lu, W., and Zhao, M. (2023). Targeted CD7 CAR T-cells for treatment of T-Lymphocyte leukemia and lymphoma and acute myeloid leukemia: recent advances. Front. Immunol. *14*, 1170968. https://doi.org/10.3389/fimmu.2023.1170968.

19. Aparicio, C., Acebal, C., and González-Vallinas, M. (2023). Current approaches to develop "off-the-shelf" chimeric antigen receptor (CAR)-T cells for cancer treatment: a systematic review. Exp. Hematol. Oncol. *12*, 73. https://doi.org/10.1186/s40164-023-00435-w.

20. Diorio, C., Murray, R., Naniong, M., Barrera, L., Camblin, A., Chukinas, J., Coholan, L., Edwards, A., Fuller, T., Gonzales, C., et al. (2022). Cytosine Base Editing Enables Quadruple-Edited Allogeneic CAR-T Cells for T-ALL. Blood *140*, 619–629. https://doi.org/10.1182/blood.2022015825.

21. Mangeot, P.E., Risson, V., Fusil, F., Marnef, A., Laurent, E., Blin, J., Mournetas, V., Massouridès, E., Sohier, T.J.M., Corbin, A., et al. (2019). Genome editing in primary cells and in vivo using viral-derived Nanoblades loaded with Cas9-sgRNA ribonucleoproteins. Nat. Commun. *10*, 45. https://doi.org/10.1038/s41467-018-07845-z.

22. Hamilton, J.R., Tsuchida, C.A., Nguyen, D.N., Shy, B.R., McGarrigle, E.R., Sandoval Espinoza, C.R., Carr, D., Blaeschke, F., Marson, A., and Doudna, J.A. (2021). Targeted delivery of CRISPR-Cas9 and transgenes enables complex immune cell engineering. Cell Rep. *35*, 109207. https://doi.org/10.1016/j.celrep.2021.109207.

23. Tsuchida, C.A., Brandes, N., Bueno, R., Trinidad, M., Mazumder, T., Yu, B., Hwang, B., Chang, C., Liu, J., Sun, Y., et al. (2023). Mitigation of chromosome loss in clinical CRISPR-Cas9-engineered T cells. Cell *186*, 4567–4582.e20. https://doi.org/10.1016/j.cell.2023.08.041.

24. Nahmad, A.D., Reuveni, E., Goldschmidt, E., Tenne, T., Liberman, M., Horovitz-Fried, M., Khosravi, R., Kobo, H., Reinstein, E., Madi, A., et al. (2022). Frequent aneuploidy in primary human T cells after CRISPR–Cas9 cleavage. Nat. Biotechnol. *40*, 1807–1813. https://doi.org/10.1038/s41587-022-01377-0.

25. Rezalotfi, A., Fritz, L., Förster, R., and Bošnjak, B. (2022). Challenges of CRISPR-Based Gene Editing in Primary T Cells. Int. J. Mol. Sci. *23*, 1689. https://doi.org/10.3390/ijms23031689.

26. Montagna, C., Petris, G., Casini, A., Maule, G., Franceschini, G.M., Zanella, I., Conti, L., Arnoldi, F., Burrone, O.R., Zentilin, L., et al. (2018). VSV-G-Enveloped Vesicles for Traceless Delivery of CRISPR-Cas9. Mol. Ther. Nucleic Acids *12*, 453–462. https://doi.org/10.1016/j.omtn.2018.05.010.

27. Banskota, S., Raguram, A., Suh, S., Du, S.W., Davis, J.R., Choi, E.H., Wang, X., Nielsen, S.C., Newby, G.A., Randolph, P.B., et al. (2022). Engineered virus-like particles for efficient in vivo delivery of therapeutic proteins. Cell *185*, 250–265.e16. https://doi.org/10.1016/j.cell.2021.12.021.

28. Hamilton, J.R., Chen, E., Perez, B.S., Espinoza, C.R.S., and Doudna, J.A. (2022). Cell type-programmable genome editing with enveloped delivery vehicles. Preprint at bioRxiv *2022*. https://doi.org/10.1101/2022.08.24.505004.

29. Gutierrez-Guerrero, A., Recalde, M.J.A., Mangeot, P.E., Costa, C., Bernadin, O., Périan, S., Fusil, F., Froment, G., Martinez-Turtos, A., Krug, A., et al. (2021). Baboon Envelope Pseudotyped "Nanoblades" Carrying Cas9/gRNA Complexes Allow Efficient Genome Editing in Human T, B, and CD34+ Cells and Knock-in of AAV6-Encoded Donor DNA in CD34+ Cells. Frontiers Genome *3*, 604371. https://doi.org/10.3389/fgeed.2021.604371.

30. Haldrup, J., Andersen, S., Labial, A.R.L., Wolff, J.H., Frandsen, F.P., Skov, T.W., Rovsing, A.B., Nielsen, I., Jakobsen, T.S., Askou, A.L., et al. (2023). Engineered lentivirus-derived nanoparticles (LVNPs) for delivery of CRISPR/Cas ribonucleoprotein complexes supporting base editing, prime editing and in vivo gene modification. Nucleic Acids Res. *51*, 10059–10074. https://doi.org/10.1093/nar/gkad676.

31. Jo, D.-H., Kaczmarek, S., Shin, O., Wang, L., Cowan, J., McComb, S., and Lee, S.-H. (2023). Simultaneous engineering of natural killer cells for CAR transgenesis and CRISPR-Cas9 knockout using retroviral particles. Mol. Ther. Methods Clin. Dev. *29*, 173–184. https://doi.org/10.1016/j.omtm.2023.03.006.

32. Hamilton, J.R., Chen, E., Perez, B.S., Sandoval Espinoza, C.R., Kang, M.H., Trinidad, M., Ngo, W., and Doudna, J.A. (2024). In vivo human T cell engineering with enveloped delivery vehicles. Nat. Biotechnol. *42*, 1684–1692. https://doi.org/10.1038/s41587-023-02085-z.

33. An, M., Raguram, A., Du, S.W., Banskota, S., Davis, J.R., Newby, G.A., Chen, P.Z., Palczewski, K., and Liu, D.R. (2024). Engineered virus-like particles for transient delivery of prime editor ribonucleoprotein complexes in vivo. Nat. Biotechnol. *42*, 1526–1537. https://doi.org/10.1038/s41587-023-02078-y.

34. Botchkarev, V.V., Harrington, S., Stoppato, M., Justen, A., Kimber, C., Kapuria, A., Gibson, K.M., Chu, C.-S., Xu, Y., Haugh, K., et al. (2025). In vivo gene editing of human hematopoietic stem and progenitor cells using envelope-engineered virus-like particles. Nat. Biotechnol. 1–13. https://doi.org/10.1038/s41587-025-02915-2.

35. Lyu, P., Javidi-Parsijani, P., Atala, A., and Lu, B. (2019). Delivering Cas9/sgRNA ribonucleoprotein (RNP) by lentiviral capsid-based bionanoparticles for efficient 'hit-and-run' genome editing. Nucleic Acids Res. *47*, e99. https://doi.org/10.1093/nar/gkz605.

36. Gee, P., Lung, M.S.Y., Okuzaki, Y., Sasakawa, N., Iguchi, T., Makita, Y., Hozumi, H., Miura, Y., Yang, L.F., Iwasaki, M., et al. (2020). Extracellular nanovesicles for packaging of CRISPR-Cas9 protein and sgRNA to induce therapeutic exon skipping. Nat. Commun. *11*, 1334. https://doi.org/10.1038/s41467-020-14957-y.

37. Son, S.H., Woo, S., Choi, A., Ahn, S., and Yoo, H.C. (2026). Advances in Engineered Virus-Like Particles for Genome Editing and Therapy. BioDrugs *40*, 317–333. https://doi.org/10.1007/s40259-026-00767-z.

38. Chen, B., Gilbert, L.A., Cimini, B.A., Schnitzbauer, J., Zhang, W., Li, G.-W., Park, J., Blackburn, E.H., Weissman, J.S., Qi, L.S., and Huang, B. (2013). Dynamic Imaging of Genomic Loci in Living Human Cells by an Optimized CRISPR/Cas System. Cell *155*, 1479–1491. https://doi.org/10.1016/j.cell.2013.12.001.

39. Lazzarotto, C.R., Malinin, N.L., Li, Y., Zhang, R., Yang, Y., Lee, G., Cowley, E., He, Y., Lan, X., Jividen, K., et al. (2020). CHANGE-seq reveals genetic and epigenetic effects on CRISPR–Cas9 genome-wide activity. Nat. Biotechnol. *38*, 1317–1327. https://doi.org/10.1038/s41587-020-0555-7.

40. Lee, W.G., Demirci, U., and Khademhosseini, A. (2009). Microscale electroporation: challenges and perspectives for clinical applications. Integr. Biol. *1*, 242–251. https://doi.org/10.1039/b819201d.

41. Bachmann, M.F., van Damme, P., Lienert, F., and Schwarz, T.F. (2025). Virus-like particles: a versatile and effective vaccine platform. Expert Rev. Vaccines *24*, 444–456. https://doi.org/10.1080/14760584.2025.2508517.

42. Gupta, R., Arora, K., Roy, S.S., Joseph, A., Rastogi, R., Arora, N.M., and Kundu, P.K. (2023). Platforms, advances, and technical challenges in virus-like particles-based vaccines. Front. Immunol. *14*, 1123805. https://doi.org/10.3389/fimmu.2023.1123805.

43. Kaczmarczyk, S.J., Sitaraman, K., Young, H.A., Hughes, S.H., and Chatterjee, D.K. (2011). Protein delivery using engineered virus-like particles. Proc. Natl. Acad. Sci. USA *108*, 16998–17003. https://doi.org/10.1073/pnas.1101874108.

44. Bell, N.M., and Lever, A.M.L. (2013). HIV Gag polyprotein: processing and early viral particle assembly. Trends Microbiol. *21*, 136–144. https://doi.org/10.1016/j.tim.2012.11.006.

45. Perry, C., and Rayat, A.C.M.E. (2021). Lentiviral Vector Bioprocessing. Viruses *13*, 268. https://doi.org/10.3390/v13020268.

46. Bauler, M., Roberts, J.K., Wu, C.-C., Fan, B., Ferrara, F., Yip, B.H., Diao, S., Kim, Y.-I., Moore, J., Zhou, S., et al. (2020). Production of Lentiviral Vectors Using Suspension Cells Grown in Serum-free Media. Mol. Ther. Methods Clin. Dev. *17*, 58–68. https://doi.org/10.1016/j.omtm.2019.11.011.

47. Bauler, M., Ferrara, F., Lowe, B., Beard, J.A., Wincek, C., Wielgosz, M.M., Park, J.J., Shang, N., Nandy, S., Li, C., et al. (2024). Genetic alteration of SJ293TS cells and modification of serum-free media enhances lentiviral vector production. Mol. Ther. Methods Clin. Dev. *32*, 101270. https://doi.org/10.1016/j.omtm.2024.101270.

48. Bernadin, O., Amirache, F., Girard-Gagnepain, A., Moirangthem, R.D., Lévy, C., Ma, K., Costa, C., Nègre, D., Reimann, C., Fenard, D., et al. (2019). Baboon envelope LVs efficiently transduced human adult, fetal, and progenitor T cells and corrected SCID-X1 T-cell deficiency. Blood Adv. *3*, 461–475. https://doi.org/10.1182/bloodadvances.2018027508.

49. Costa, C., Hypolite, G., Bernadin, O., Lévy, C., Cosset, F.-L., Asnafi, V., Macintyre, E., Verhoeyen, E., and Tesio, M. (2017). Baboon envelope pseudotyped lentiviral vectors: a highly efficient new tool to genetically manipulate T-cell acute lymphoblastic leukaemia-initiating cells. Leukemia *31*, 977–980. https://doi.org/10.1038/leu.2016.372.

50. Wienert, B., Shin, J., Zelin, E., Pestal, K., and Corn, J.E. (2018). In vitro–transcribed guide RNAs trigger an innate immune response via the RIG-I pathway. PLoS Biol. *16*, e2005840. https://doi.org/10.1371/journal.pbio.2005840.

51. Arndt, B., Poltorak, M., Kowtharapu, B.S., Reichardt, P., Philipsen, L., Lindquist, J.A., Schraven, B., and Simeoni, L. (2013). Analysis of TCR activation kinetics in primary human T cells upon focal or soluble stimulation. J. Immunol. Methods *387*, 276–283. https://doi.org/10.1016/j.jim.2012.11.006.

52. Lamers, C.H.J., van Elzakker, P., van Steenbergen, S.C.L., Sleijfer, S., Debets, R., and Gratama, J.W. (2008). Retronectin®-assisted retroviral transduction of primary human T lymphocytes under good manufacturing practice conditions: tissue culture bag critically determines cell yield. Cytotherapy *10*, 406–416. https://doi.org/10.1080/14653240801982961.

53. Radek, C., Bernadin, O., Drechsel, K., Cordes, N., Pfeifer, R., Sträßer, P., Mormin, M., Gutierrez-Guerrero, A., Cosset, F.L., Kaiser, A.D., et al. (2019). Vectofusin-1 Improves Transduction of Primary Human Cells with Diverse Retroviral and Lentiviral Pseudotypes, Enabling Robust, Automated Closed-System Manufacturing. Hum. Gene Ther. *30*, 1477–1493. https://doi.org/10.1089/hum.2019.157.

54. Engel, N.W., Steinfeld, I., Ryan, D., Anupindi, K., Kim, S., Wellhausen, N., Chen, L., Wilkins, K., Baker, D.J., Rommel, P.C., et al. (2025). Quadruple adenine base–edited allogeneic CAR T cells outperform CRISPR/Cas9 nuclease–engineered T cells. Proc. Natl. Acad. Sci. USA *122*, e2427216122. https://doi.org/10.1073/pnas.2427216122.

55. Wu, Y., Zeng, J., Roscoe, B.P., Liu, P., Yao, Q., Lazzarotto, C.R., Clement, K., Cole, M.A., Luk, K., Baricordi, C., et al. (2019). Highly efficient therapeutic gene editing of human hematopoietic stem cells. Nat. Med. *25*, 776–783. https://doi.org/10.1038/s41591-019-0401-y.

56. Milani, M., Annoni, A., Bartolaccini, S., Biffi, M., Russo, F., Di Tomaso, T., Raimondi, A., Lengler, J., Holmes, M.C., Scheiflinger, F., et al. (2017). Genome editing for scalable production of alloantigen-free lentiviral vectors for in vivo gene therapy. EMBO Mol. Med. *9*, 1558–1573. https://doi.org/10.15252/emmm.201708148.

57. Eyquem, J., Mansilla-Soto, J., Giavridis, T., van der Stegen, S.J.C., Hamieh, M., Cunanan, K.M., Odak, A., Gönen, M., and Sadelain, M. (2017). Targeting a CAR to the TRAC locus with CRISPR/Cas9 enhances tumour rejection. Nature *543*, 113–117. https://doi.org/10.1038/nature21405.

58. Labun, K., Montague, T.G., Krause, M., Torres Cleuren, Y.N., Tjeldnes, H., and Valen, E. (2019). CHOPCHOP v3: expanding the CRISPR web toolbox beyond genome editing. Nucleic Acids Res. *47*, W171–W174. https://doi.org/10.1093/nar/gkz365.

59. Riberdy, J.M., Zhou, S., Zheng, F., Kim, Y.-I., Moore, J., Vaidya, A., Throm, R.E., Sykes, A., Sahr, N., Bonifant, C.L., et al. (2020). The Art and Science of Selecting a CD123-Specific Chimeric Antigen Receptor for Clinical Testing. Mol. Ther. Methods Clin. Dev. *18*, 571–581. https://doi.org/10.1016/j.omtm.2020.06.024.

60. Nguyen, P., Okeke, E., Clay, M., Haydar, D., Justice, J., O'Reilly, C., Pruett-Miller, S., Papizan, J., Moore, J., Zhou, S., et al. (2020). Route of 41BB/41BBL Costimulation Determines Effector Function of B7-H3-CAR.CD28ζ T Cells. Mol. Ther. Oncolytics *18*, 202–214. https://doi.org/10.1016/j.omto.2020.06.018.

61. Imai, C., Mihara, K., Andreansky, M., Nicholson, I.C., Pui, C.-H., Geiger, T.L., and Campana, D. (2004). Chimeric receptors with 4-1BB signaling capacity provoke potent cytotoxicity against acute lymphoblastic leukemia. Leukemia *18*, 676–684. https://doi.org/10.1038/sj.leu.2403302.

62. Vink, C.A., Counsell, J.R., Perocheau, D.P., Karda, R., Buckley, S.M.K., Brugman, M.H., Galla, M., Schambach, A., McKay, T.R., Waddington, S.N., and Howe, S.J. (2017). Eliminating HIV-1 Packaging Sequences from Lentiviral Vector Proviruses Enhances Safety and Expedites Gene Transfer for Gene Therapy. Mol. Ther. *25*, 1790–1804. https://doi.org/10.1016/j.ymthe.2017.04.028.

63. Connelly, J.P., and Pruett-Miller, S.M. (2019). CRIS.py: A Versatile and High-throughput Analysis Program for CRISPR-based Genome Editing. Sci. Rep. *9*, 4194. https://doi.org/10.1038/s41598-019-40896-w.

OMTA, Volume *34*

Supplemental information

Co-delivery of lentiviral vectors and Cas9-containing virus-like particles enables rapid, scalable manufacture of gene-edited CAR T cells

Francesca Ferrara, Matthew M. Wielgosz, Jeoungeun J. Park, Matthew Bauler, Chris Wincek, Nataly Mier, Yin Su, Li Tang, Brandon R. Lowe, Jordan A. Beard, Deanna M. Langfitt, Sheng Zhou, and Robert E. Throm

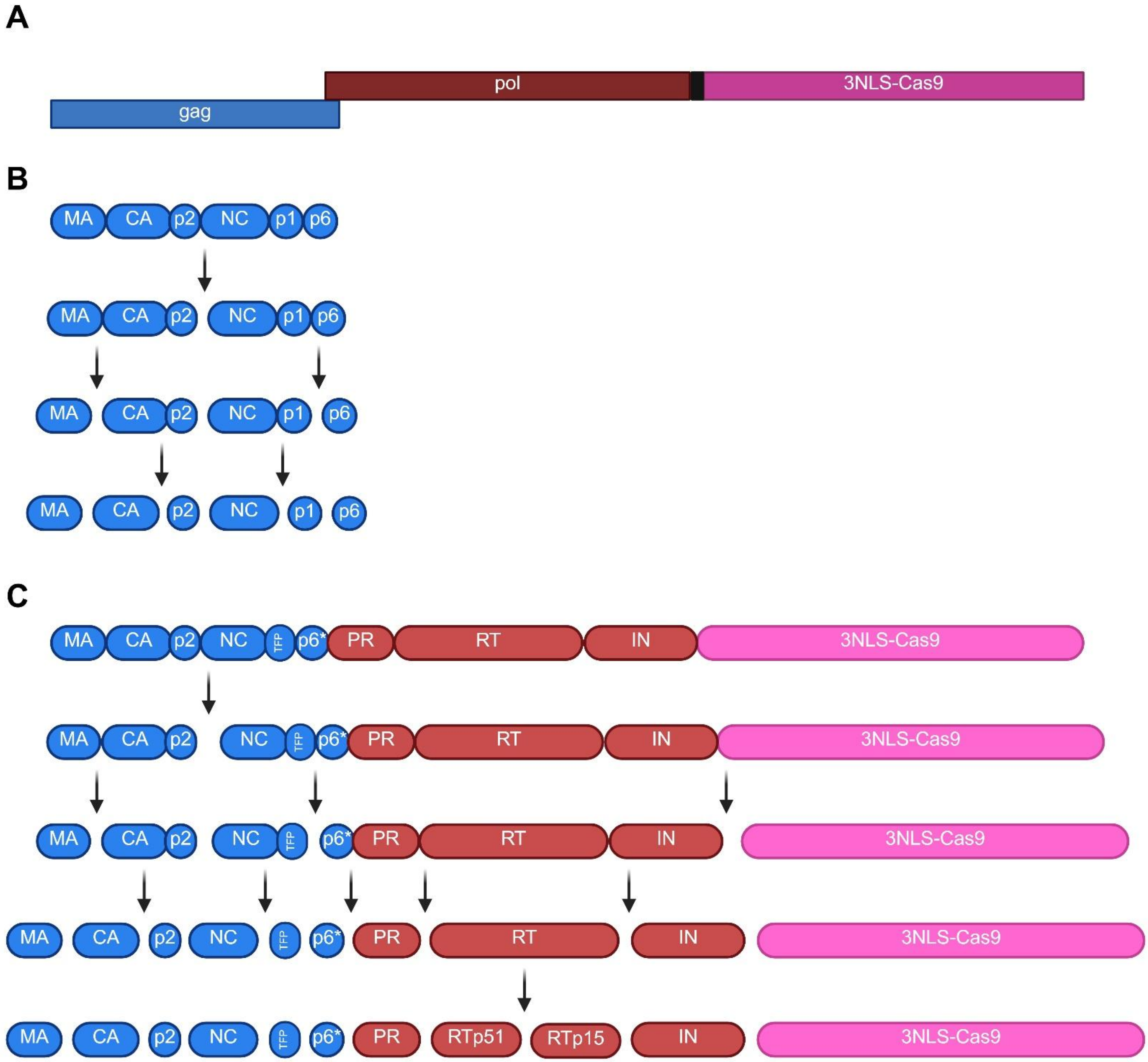

Figure S1: Expected translation and processing of the HIV-1 Gag-Pol-Cas9 fusion polyprotein.
Cas9 sequence was fused to the C-terminal domain of the Gag-Pol sequence using the HIV-1 matrix-capsid cleavage (QVSQNY↑PIVQNI) linker peptide (A). Two different polyproteins are translated from a common transcript and processed by the HIV-1 protease during particle maturation: a Gag polyprotein (B) and a Gag-Pol-Cas9 polyprotein (C).

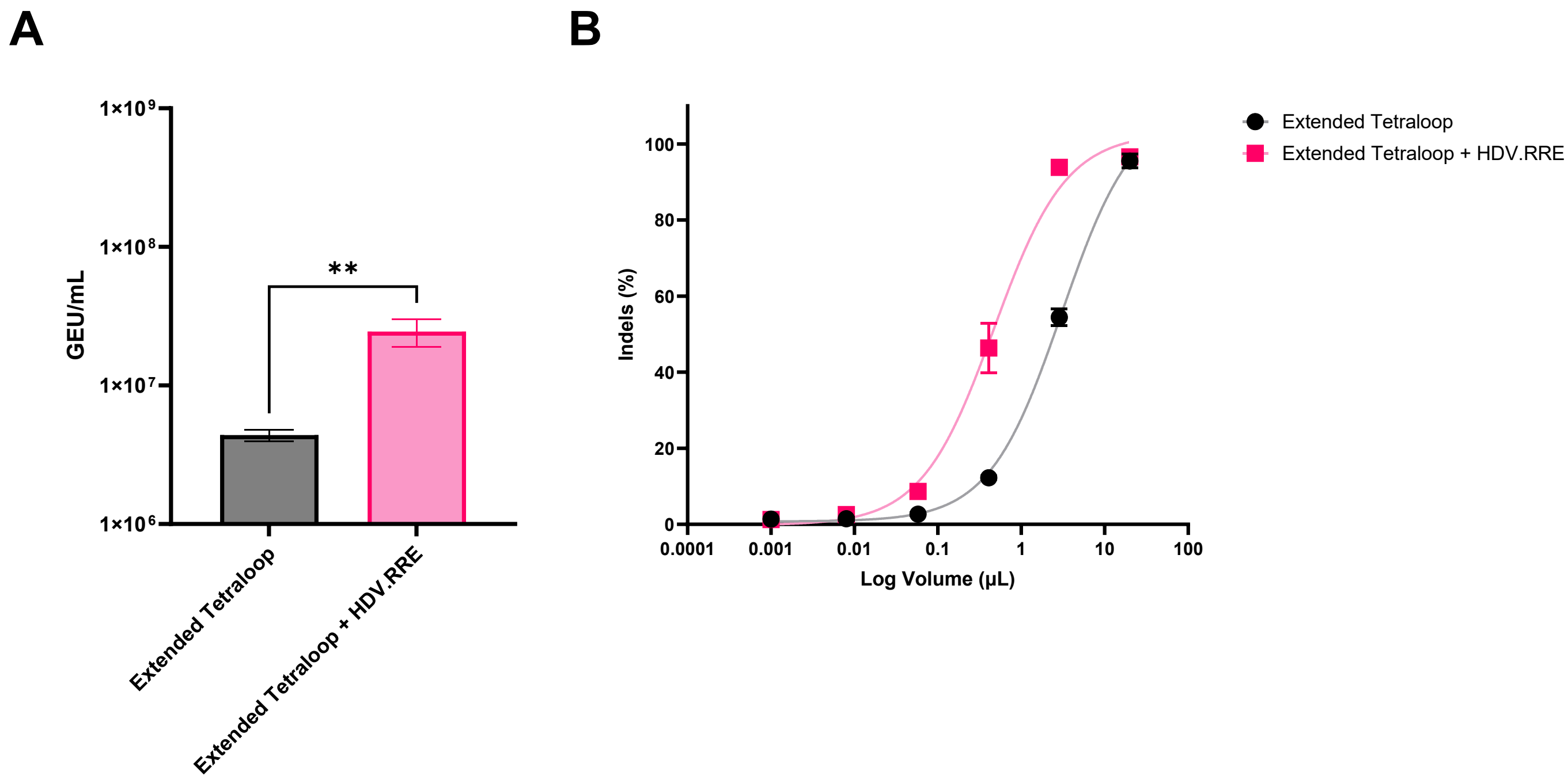

Figure S2: Effect of the Hepatitis D virus ribozyme and Rev Response Element in sgRNA scaffold for Cas9 VLP production

VLPs were produced using a plasmid expressing B2M sgRNA with a modified scaffold (extended tetraloop stem and without a premature termination sequence) with and without the HDV ribozyme and RRE sequences at its 3. Editing activity was evaluated in transduction of HOS-mCat1 cells and is reported as gene editing units per mL (GEU/mL) (A) and as percentage of indels per µL of VLP tested (B). n=3.

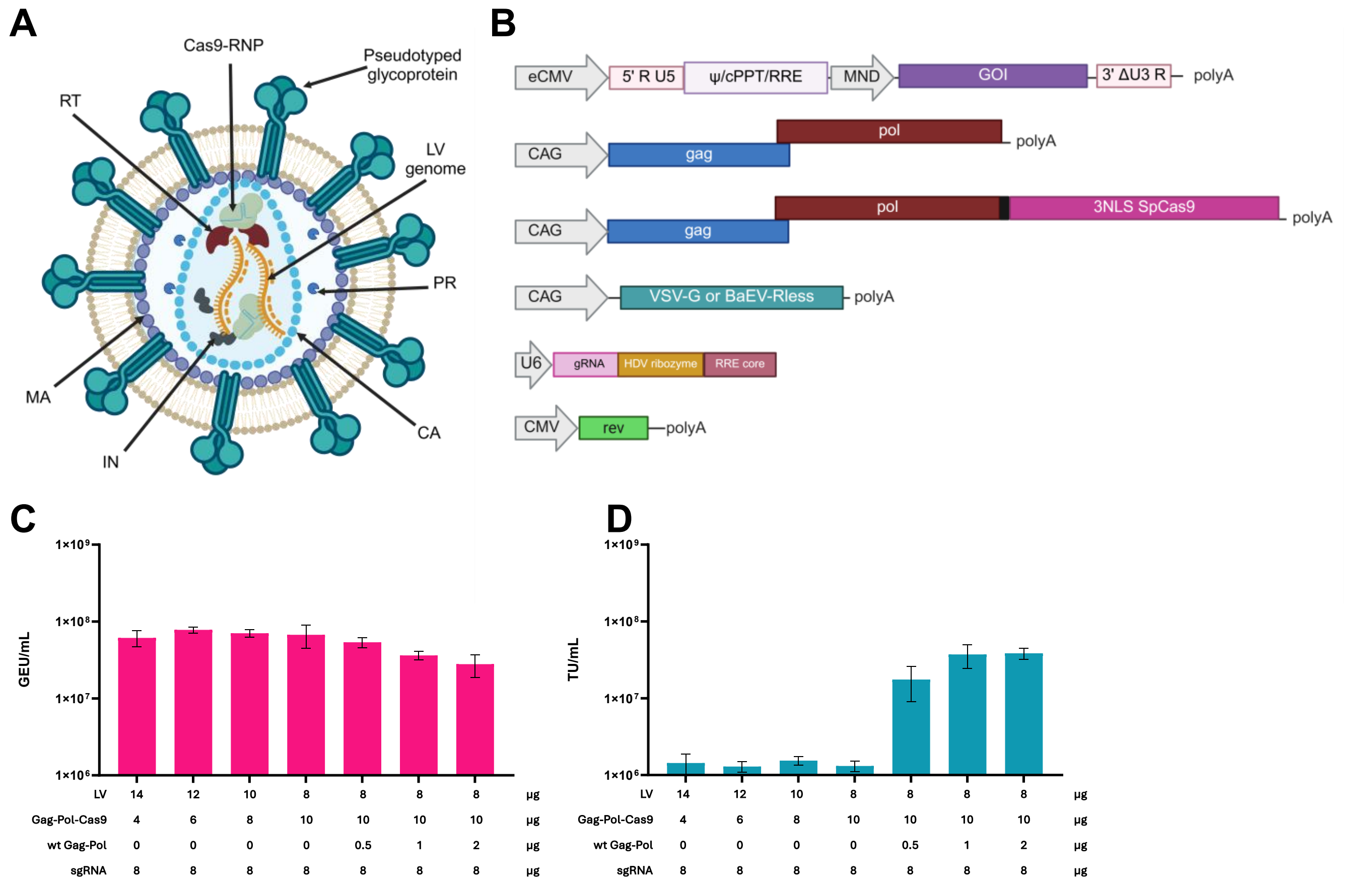

Figure S3: Production and activity of Lentiviral Vector Cas9 (LVC)
Schematic representation of the predicted structure of LVC based on HIV-1 structure (A). Design of plasmids used to produce LVC: a LV vector transfer plasmid; a structural plasmid expressing HIV-1 Gag-Pol; a structural plasmid expressing HIV-1 Gag-Pol fused with 3NLS-Cas9; a plasmid expressing an envelope protein (VSV-G); a plasmid expressing an sgRNA; and a plasmid encoding HIV-1 Rev (B). Evaluation of LVC gene editing activity on HOS-mCat1 cells produced using various plasmid ratios (C-D). Editing activity is reported as gene editing units per mL (GEU/mL), and transduction activity is reported as transduction units per mL(TU/mL). n = 3.

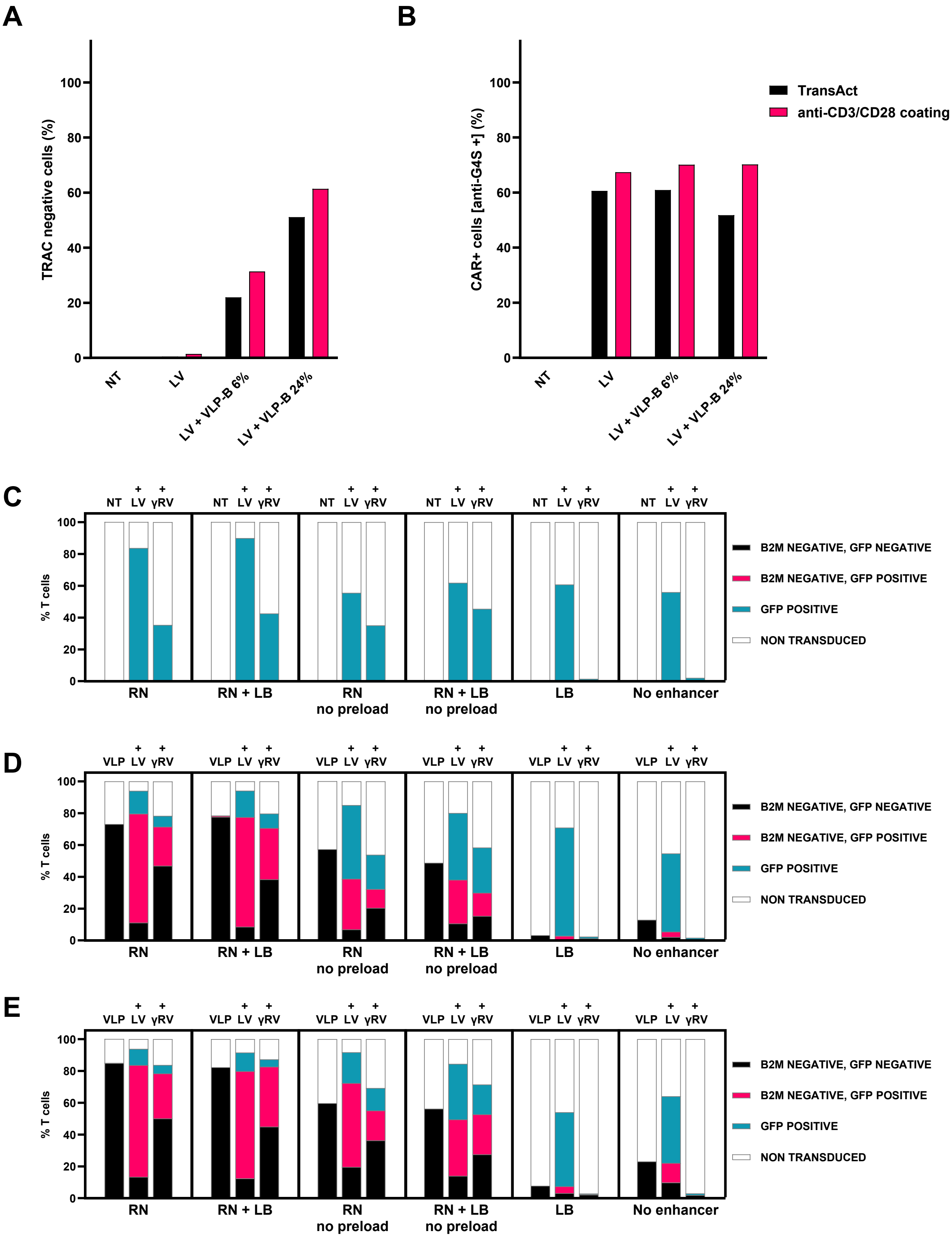

A
TRAC negative cells (%)
0
20
40
60
80
100
NT
LV
LV + VLP-B 6%
LV + VLP-B 24%
B
CAR+ cells [anti-G4S +] (%)
0
20
40
60
80
100
NT
LV
LV + VLP-B 6%
LV + VLP-B 24%
TransAct
anti-CD3/CD28 coating
C
NT
+ LV
+ γRV
% T cells
RN
RN + LB
RN no preload
RN + LB no preload
LB
No enhancer
B2M NEGATIVE, GFP NEGATIVE
B2M NEGATIVE, GFP POSITIVE
GFP POSITIVE
NON TRANSDUCED
D
VLP
+ LV
+ γRV
% T cells
RN
RN + LB
RN no preload
RN + LB no preload
LB
No enhancer
B2M NEGATIVE, GFP NEGATIVE
B2M NEGATIVE, GFP POSITIVE
GFP POSITIVE
NON TRANSDUCED
E
VLP
+ LV
+ γRV
% T cells
RN
RN + LB
RN no preload
RN + LB no preload
LB
No enhancer
B2M NEGATIVE, GFP NEGATIVE
B2M NEGATIVE, GFP POSITIVE
GFP POSITIVE
NON TRANSDUCED

Figure S4: Optimization of T-cells transduction conditions

Healthy donor T-cells were activated using TransAct™ or by incubation in anti-CD3 and anti-CD28 coated wells before being transduced with a CAR LV vector and two different quantities of VLPs pseudotyped with BaEV-Rless to edit the TRAC locus. TRAC knock-out (A), and CAR expressions (B) are measured using flow cytometry after staining with the anti-G4S and anti-TCR α/β antibodies.

The transduction enhancers are tested alone or in combination for transduction of GFP-expressing LV and γRV and 0 µL(C), 0.25 µl (D), or 0.75 µL (E) of VLP to edit the B2M locus. B2M knock-out and GFP expression were evaluated using flow cytometry. RN – RetroNectin; RN +LB – RetroNectin and LentiBOOST™; RN – RetroNectin without preloading; RN +LB – RetroNectin and LentiBOOST™ without preloading; LB – LentiBOOST. n=1

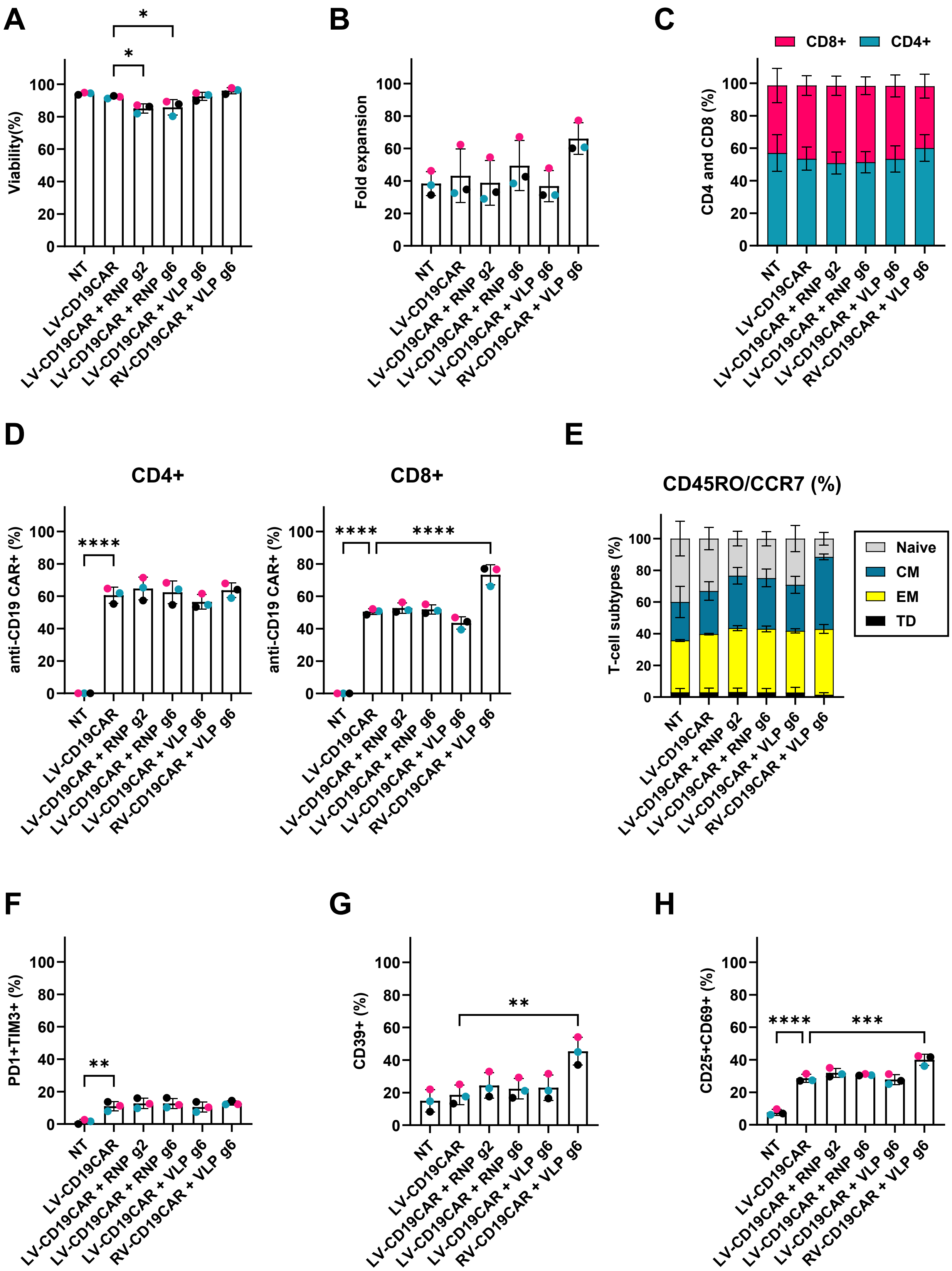
A
*
*
Viability(%)
NT
LV-CD19CAR
LV-CD19CAR + RNP g2
LV-CD19CAR + RNP g6
LV-CD19CAR + VLP g6
RV-CD19CAR + VLP g6
B
Fold expansion
C
CD8+
CD4+
CD4 and CD8 (%)
D
CD4+
CD8+
anti-CD19 CAR+ (%)
****
****
****
E
CD45RO/CCR7 (%)
T-cell subtypes (%)
Naive
CM
EM
TD
F
PD1+TIM3+ (%)
**
G
CD39+ (%)
**
H
CD25+CD69+ (%)
****
***

Figure S5: Characterization of gene edited CAR T cells.

On day 1, T cells from three healthy donors were transduced with LV vector, or co-transduced with Cas9-VLP targeting DNMT3A and LV vector, or co-transduced with Cas9-VLP targeting DNMT3A and γRV vector, or transduced with LV then electroporated with RNP the next day. On day 2, cell viability was assessed post-transduction and post-electroporation (A). On day 7, cell fold expansion was calculated (B). $CD4^+$ and $CD8^+$ cells percentages are evaluated by flow cytometry (C); CAR expression in percentage and as mean fluorescence intensity is evaluated in $CD4^+$ and $CD8^+$ cell subsets(D). T-cell subtypes were characterized based on CD45RO and CCR7 expression (E); Exhaustion markers PD-1, TIM3, and CD39 are assessed using flow cytometry (F-G). The activation markers CD25 and CD69 are also monitored (H). One-way ANOVA is used for statistical analysis (* $p \leq 0.05$; ** $p \leq 0.01$; *** $p \leq 0.001$; **** $p \leq 0.0001$). n=3

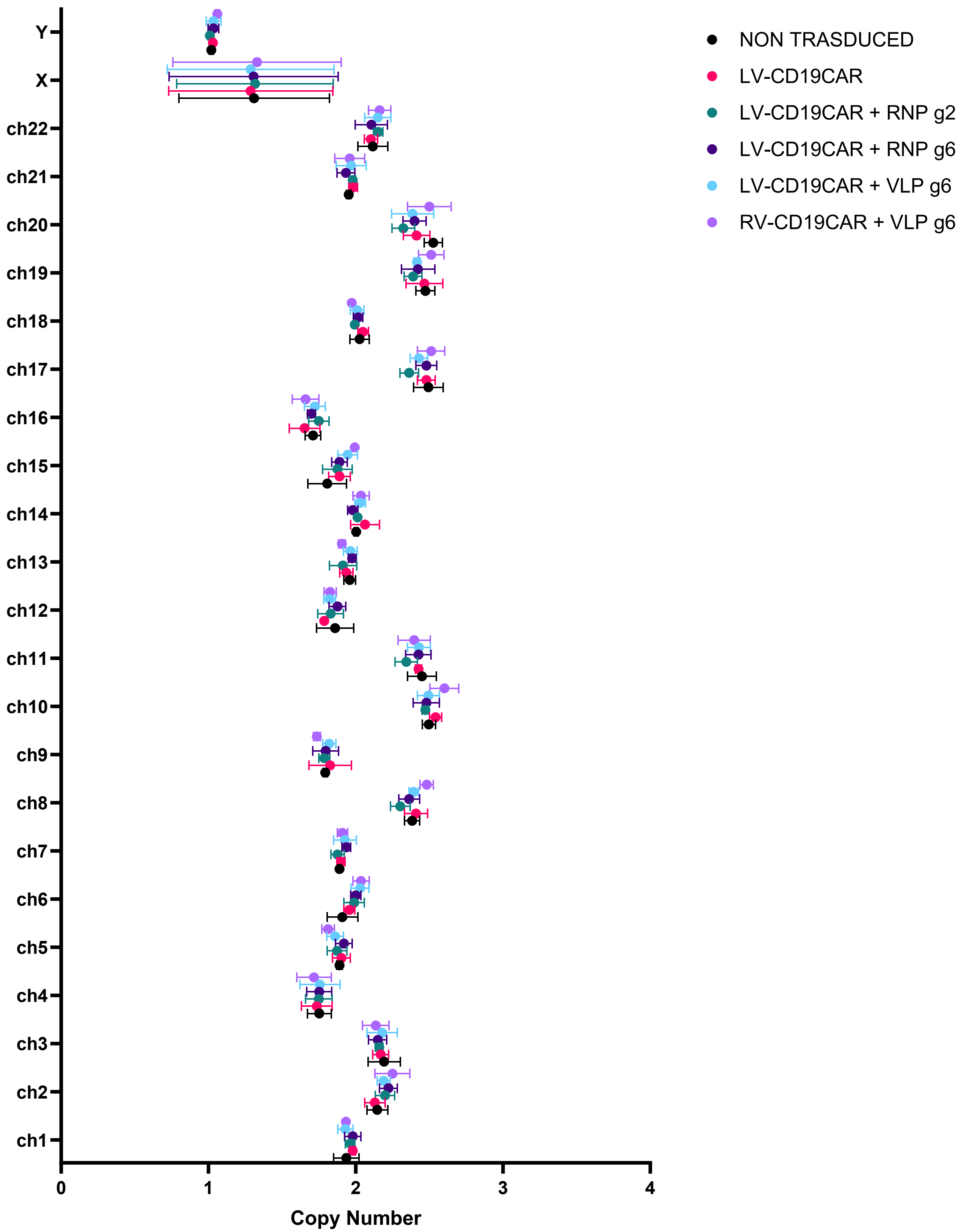
Y
X
ch22
ch21
ch20
ch19
ch18
ch17
ch16
ch15
ch14
ch13
ch12
ch11
ch10
ch9
ch8
ch7
ch6
ch5
ch4
ch3
ch2
ch1
0
1
2
3
4
Copy Number
NON TRASDUCED
LV-CD19CAR
LV-CD19CAR + RNP g2
LV-CD19CAR + RNP g6
LV-CD19CAR + VLP g6
RV-CD19CAR + VLP g6

Figure S6: Aneuploidy analysis of edited CAR T-cells

Genomic DNA of T-cells from three healthy donors co-transduced with an LV or γRV vector expressing CAR and edited by electroporation of RNPs or transduction using VLP was extracted and analyzed using the iCS-digital™ PSC: Aneuploidy test (Stem Genomics). Genomic DNA from paired, non-transduced cells were used as a control and as a baseline to establish chromosome copy number. Chromosome copy number with standard deviation was plotted for each chromosome. Standard deviations for the X chromosome were higher because one donor is female (two copies), and two donors are male (1 copy).

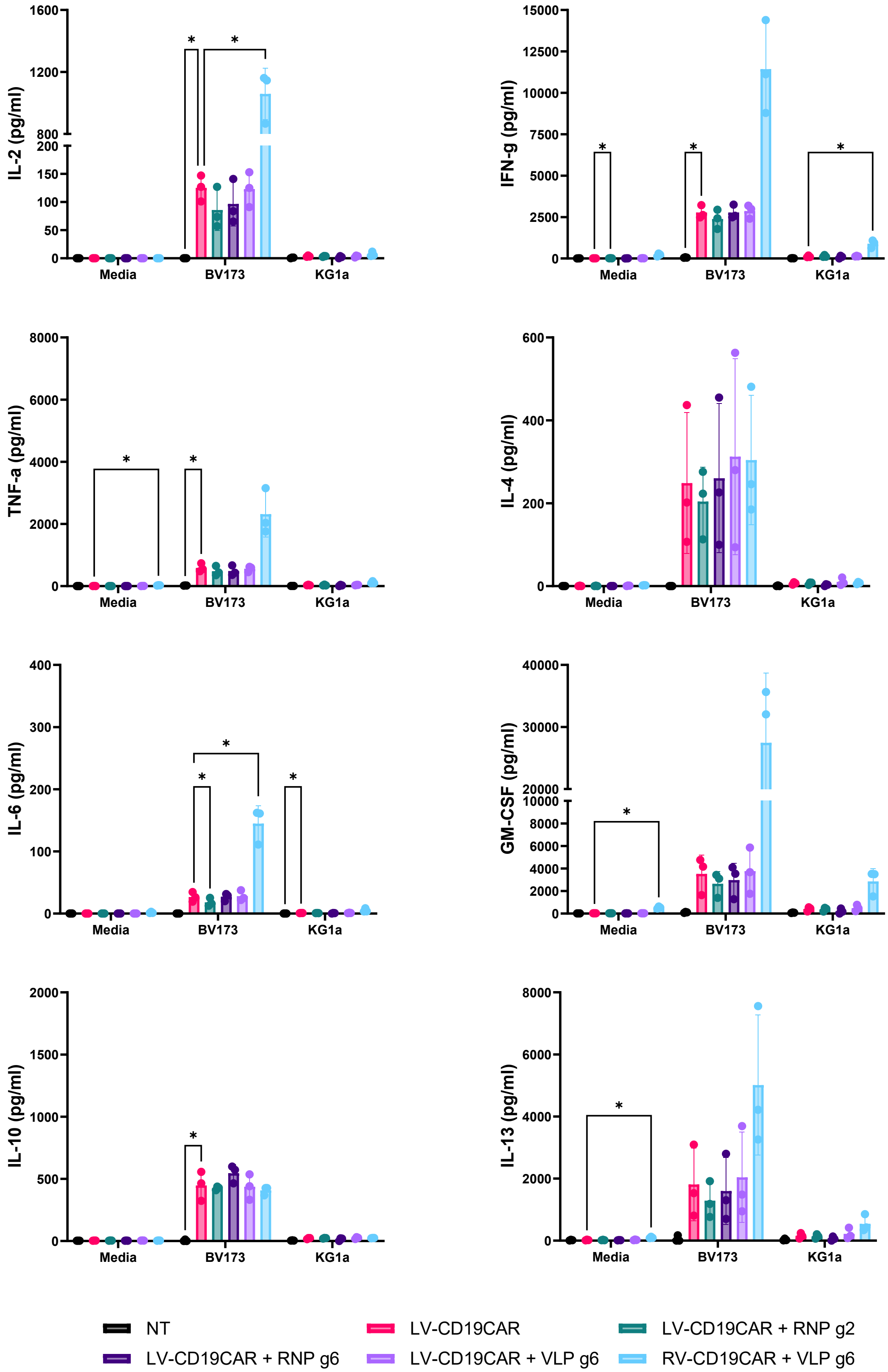
IL-2 (pg/ml)
1600
1200
800
200
150
100
50
0
*
*
Media
BV173
KG1a
IFN-g (pg/ml)
15000
12500
10000
7500
5000
2500
0
*
*
*
Media
BV173
KG1a
TNF-a (pg/ml)
8000
6000
4000
2000
0
*
*
Media
BV173
KG1a
IL-4 (pg/ml)
600
400
200
0
Media
BV173
KG1a
IL-6 (pg/ml)
400
300
200
100
0
*
*
*
Media
BV173
KG1a
GM-CSF (pg/ml)
40000
30000
20000
10000
8000
6000
4000
2000
0
*
Media
BV173
KG1a
IL-10 (pg/ml)
2000
1500
1000
500
0
*
Media
BV173
KG1a
IL-13 (pg/ml)
8000
6000
4000
2000
0
*
Media
BV173
KG1a
NT
LV-CD19CAR
LV-CD19CAR + RNP g2
LV-CD19CAR + RNP g6
LV-CD19CAR + VLP g6
RV-CD19CAR + VLP g6

Figure S7: Cytokine secretion during in vitro CAR-T cell–mediated killing

DNMT3A-edited CD19 CAR T cells generated using either ribonucleoprotein (RNP) or virus-like particle (VLP) delivery were co-cultured with CD19-positive (BV173) or CD19-negative (KG1a) target cells overnight. Following co-culture, supernatants were collected and analyzed for release of IL-2, IFN-γ, TNF-α, IL-4, IL-6, GM-CSF, IL-10, and IL-13. Two-way ANOVA is used for statistical analysis (* $p \leq 0.05$; ** $p \leq 0.01$; *** $p \leq 0.001$; **** $p \leq 0.0001$). n=3

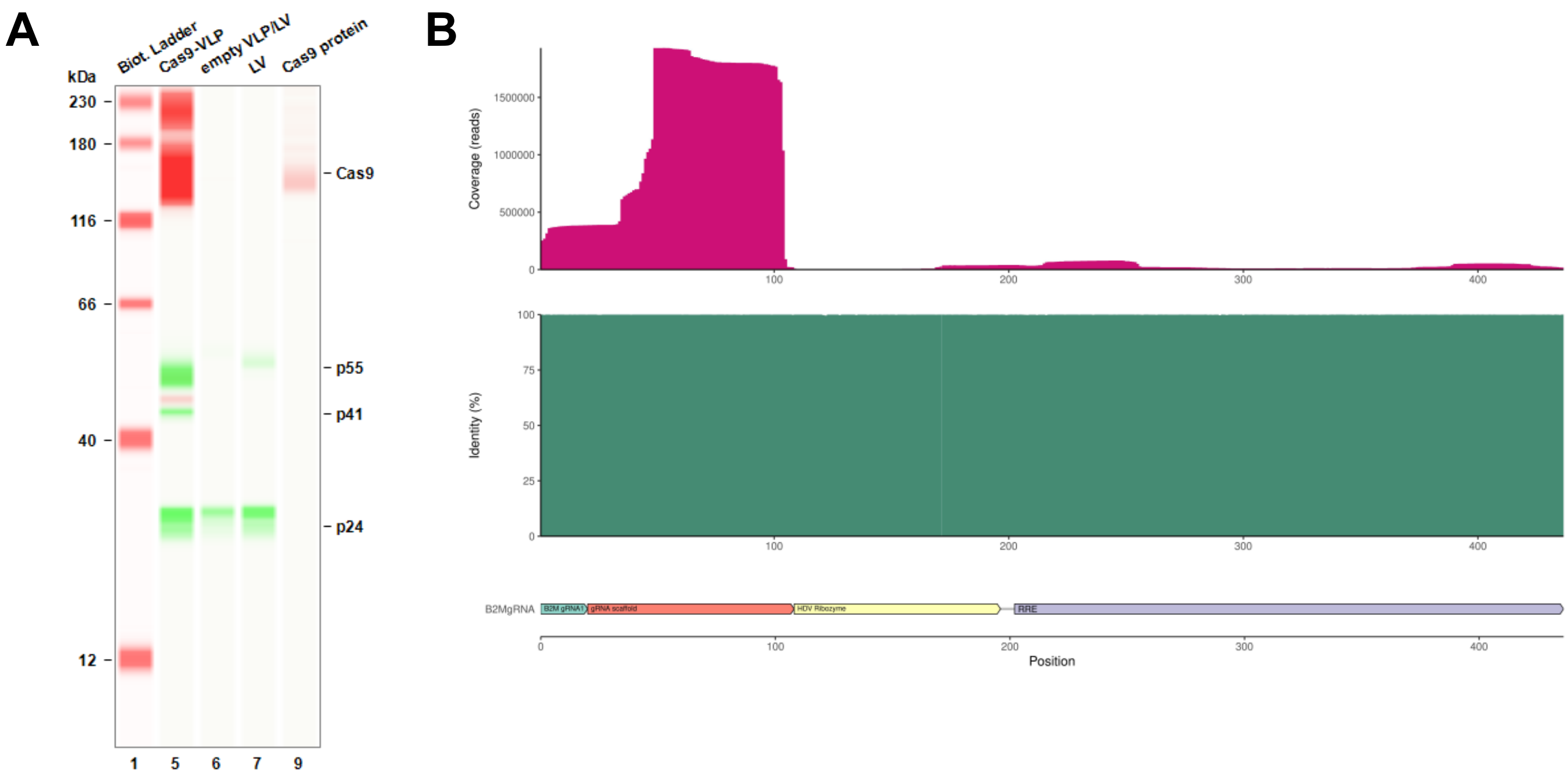

Figure S8: Characterization of Virus-Like particles
Cas9 and p24 in VLPs were detected by capillary western blotting (A). The B2M sgRNA loaded on VLP was extracted and subjected to next-generation sequencing and bioinformatic analysis to align with expected sequence (B).

A

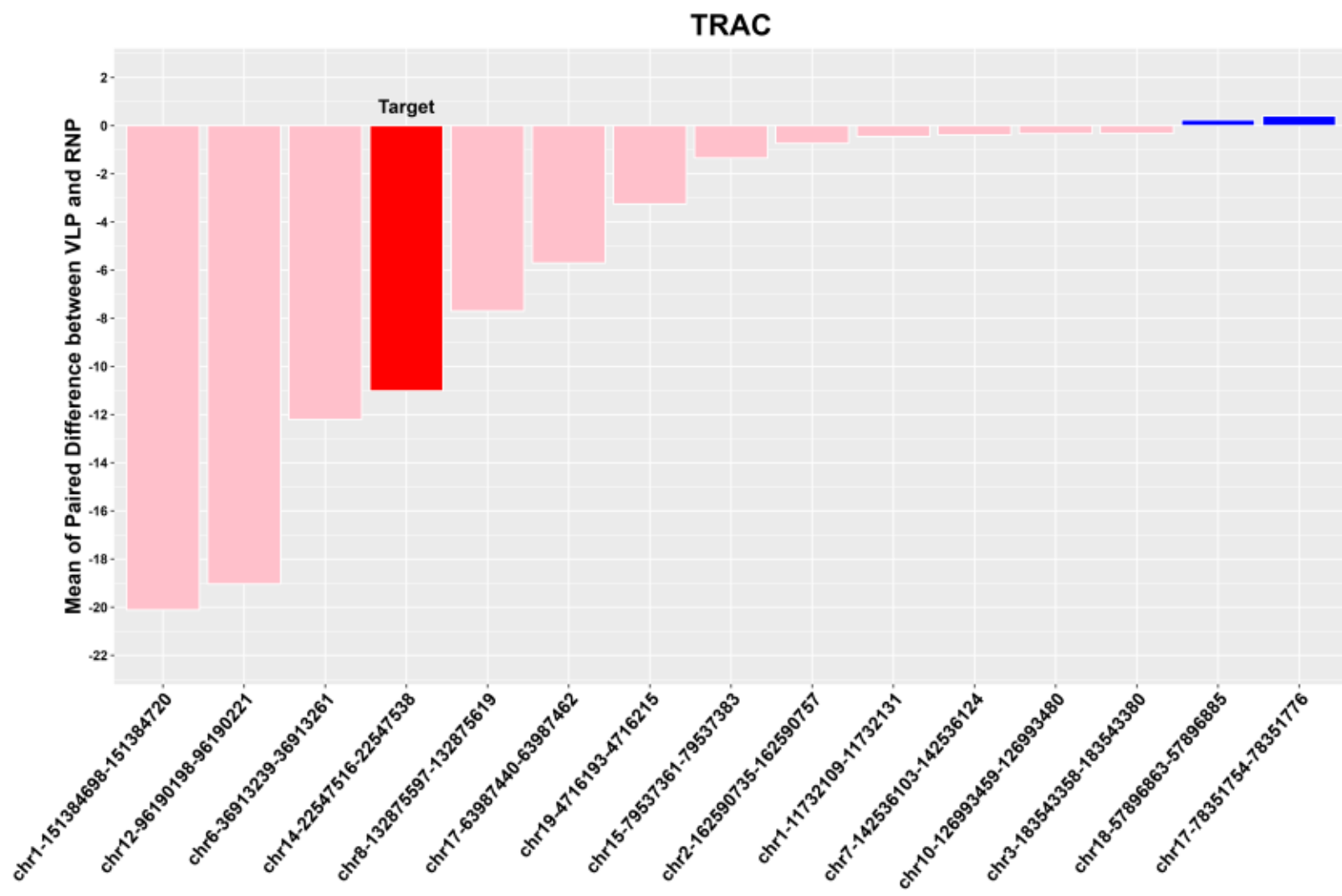
TRAC
Target
Mean of Paired Difference between VLP and RNP
chr1-151384698-151384720
chr12-96190198-96190221
chr6-36913239-36913261
chr14-22547516-22547538
chr8-132875597-132875619
chr17-63987440-63987462
chr19-4716193-4716215
chr15-79537361-79537383
chr2-162590735-162590757
chr1-11732109-11732131
chr7-142536103-142536124
chr10-126993459-126993480
chr3-183543358-183543380
chr18-57896863-57896885
chr17-78351754-78351776

B

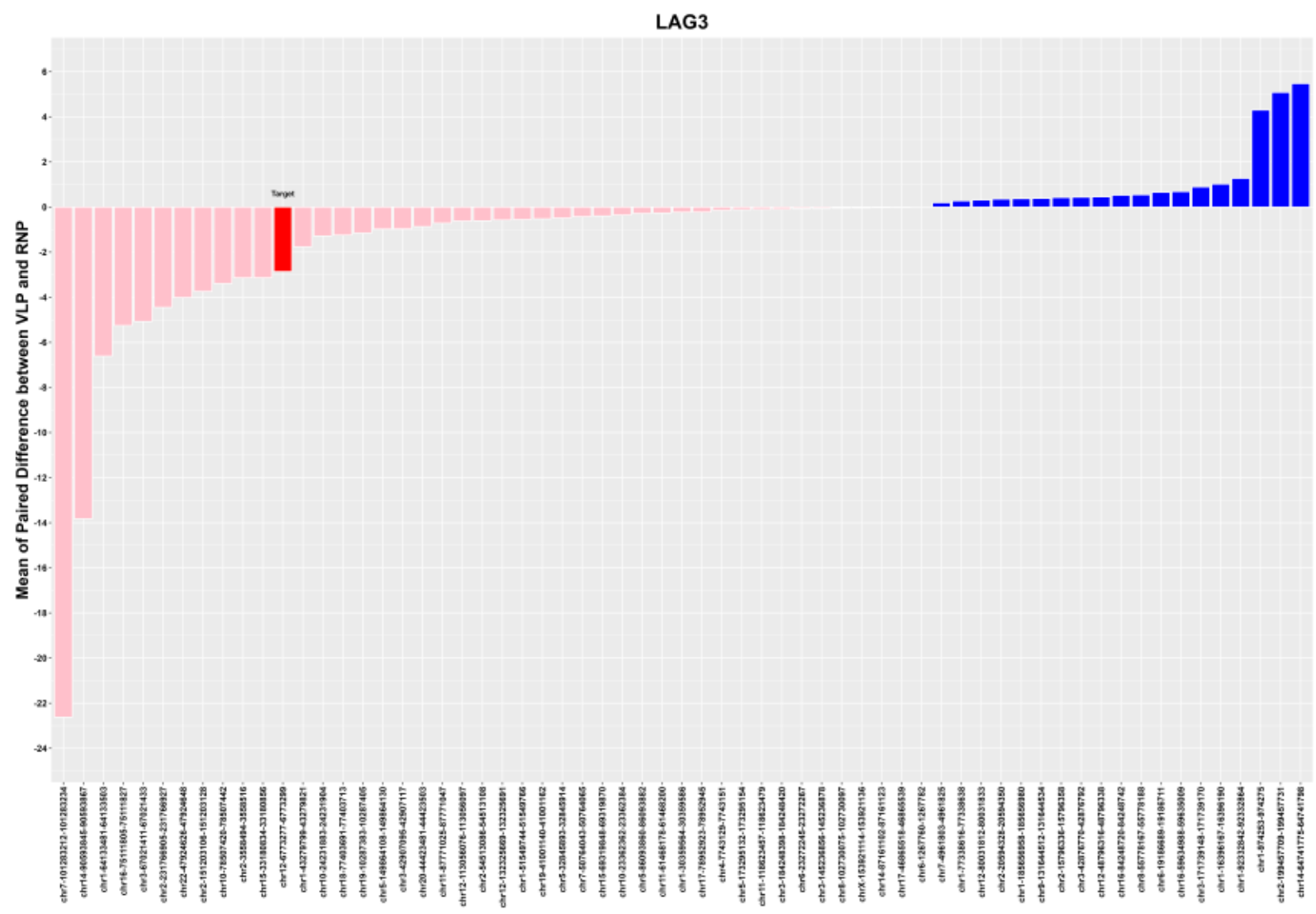
LAG3
Target
Mean of Paired Difference between VLP and RNP

C

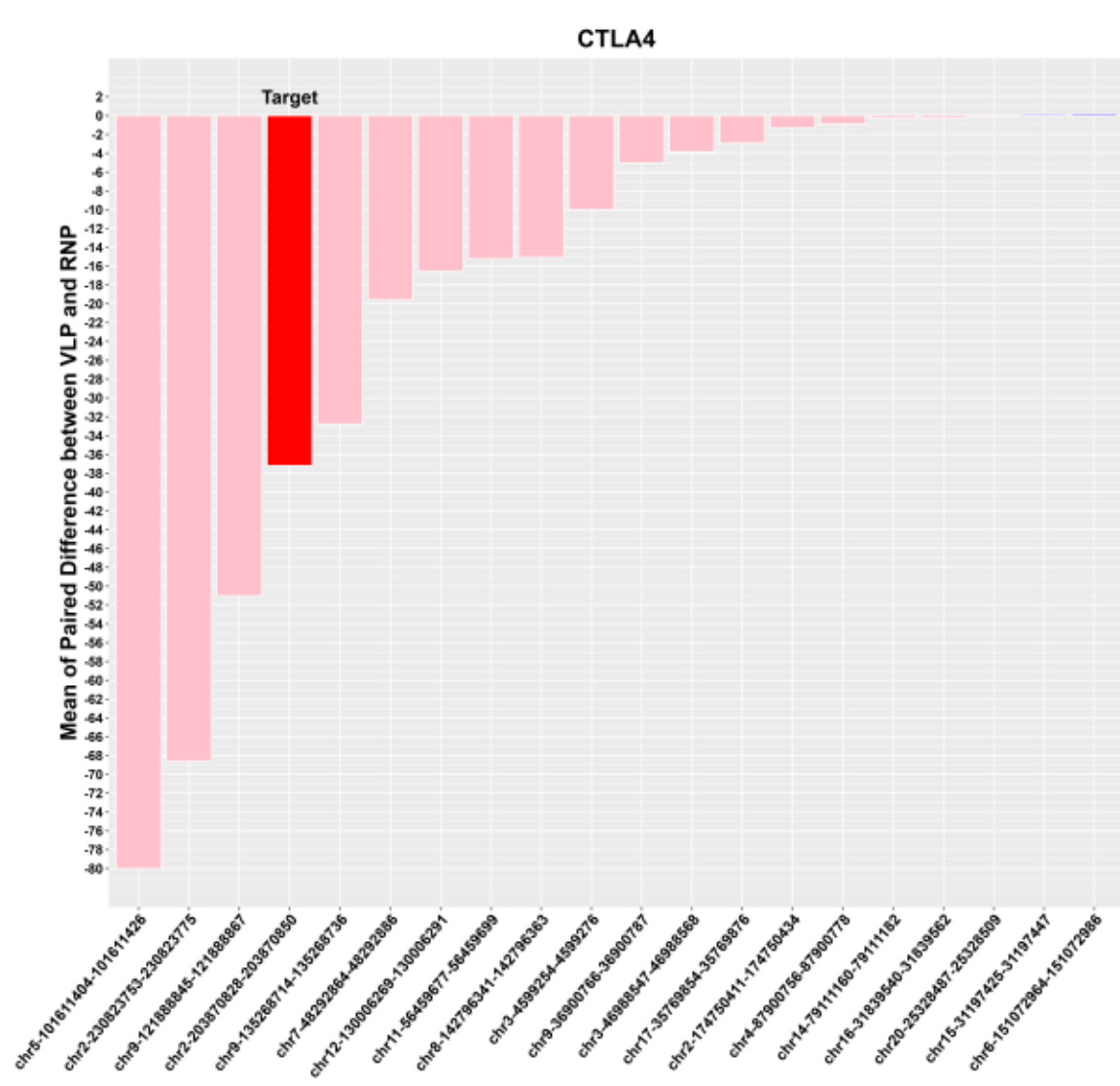
CTLA4
Target
Mean of Paired Difference between VLP and RNP
chr5-101611404-101611426
chr2-230823753-230823775
chr9-121888845-121888867
chr2-203870828-203870850
chr9-135268714-135268736
chr7-48292864-48292886
chr12-130006269-130006291
chr11-56459677-56459699
chr8-142796341-142796363
chr3-4599254-4599276
chr9-36900766-36900787
chr3-46988547-46988568
chr17-35769854-35769876
chr2-174750411-174750434
chr4-87900756-87900778
chr14-79111160-79111182
chr16-31839540-31839562
chr20-25328487-25328509
chr15-31197425-31197447
chr6-151072964-151072986

Figure S9: Mean of paired difference between off-targets detected after editing using Cas9-VLPs and electroporated RNPs

After curation of rhAmpSeq data for TRAC (A), LAG3 (B), and CTLA-4 (C), the mean of paired difference was calculated between Cas9-VLPs editing and electroporated RNPs editing for every in which editing was considered significant (above 7%). The means of paired differences were graphed and ordered to highlight which loci had significantly different editing between the two groups.

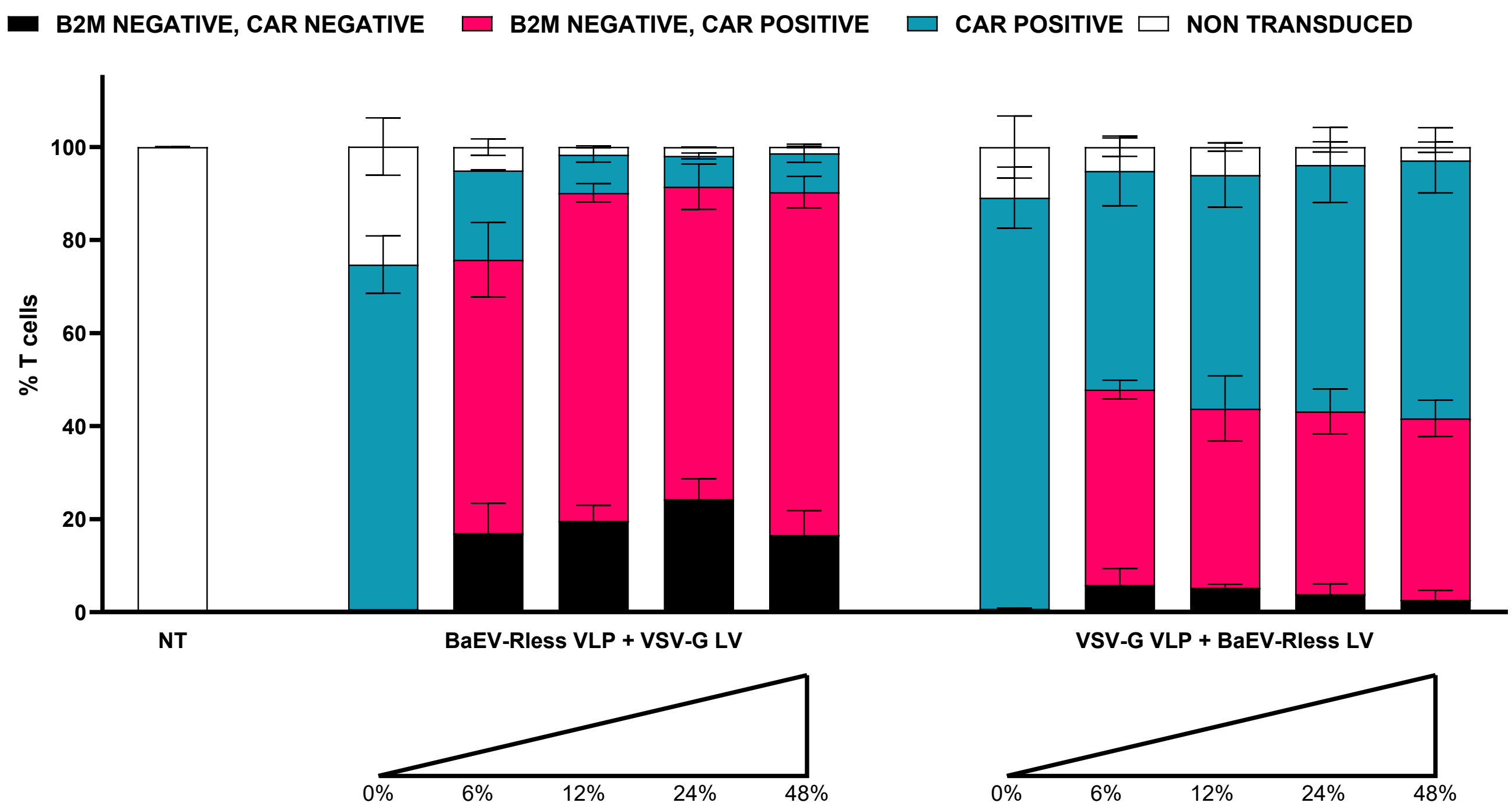

Figure S10: Evaluation of VSV-G and BaEV-Rless for pseudotyping LV and VLP
Healthy human T cells were co-transduced with either LV pseudotyped with VSV-G and an increasing amount of VLP pseudotyped with BaEV-Rless, or with LV pseudotyped with BaEV-Rless, and an increasing amount of VLP pseudotyped with VSV-G. Seven days post-transduction, CAR and TRAC expression were assessed by cell surface staining using an anti-G4S and anti-B2M. n=2

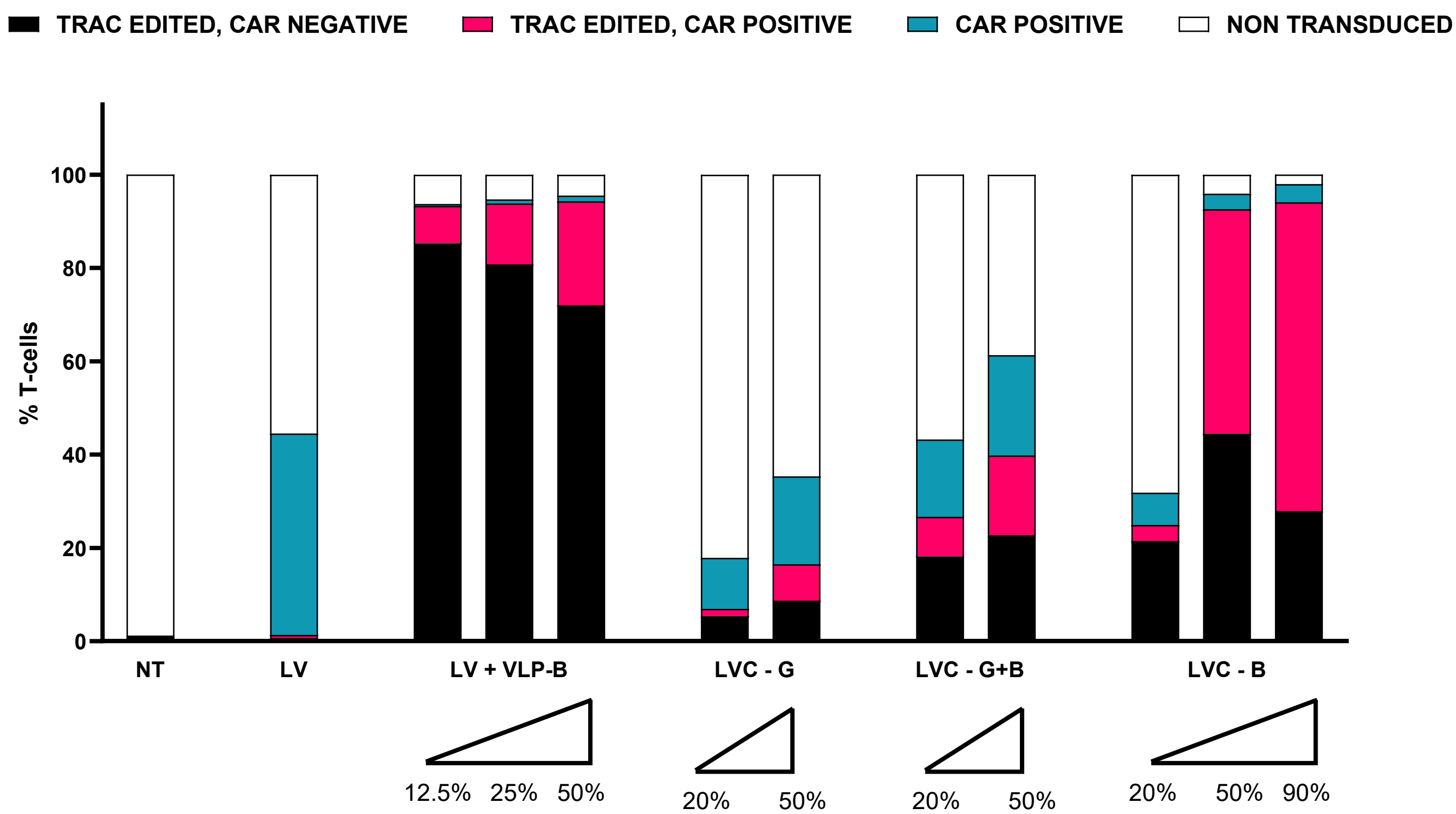

Figure S11: Comparison of transduction of LVC pseudotyped with different glycoproteins
Healthy human T cells were transduced with either VSV-G LV only, VSV-G LV and BaEV-Rless VLP, LVC pseudotyped with VSV-G, LVC pseudotyped with VSV-G and BaEV-Rless, or LVC pseudotyped with BaEV-Rless. LV and LVC expressed a bi-specific anti-CD19CD22 CAR. VLP and LVC targeted the TRAC locus. Seven days post-transduction, CAR and TRAC expression were assessed by cell surface staining using an anti-G4S and anti-TCR α/β antibodies. n=1

Table S1: Characterization of Virus-Like Particle Preparations used in healthy-donor T-cells transductions

Unique ID	Type	Envelope	Target	Figure	p24 (µg/mL)	Cas9 (µg/mL)	Average p24 (µg/mL)	Standard Deviation p24 (µg/mL)	Average Cas9 (µg/mL)	Standard Deviation Cas9 (µg/mL)
**13014**	LVC	VSV-G	B2M	2	68.50	5.17	54.43	30.26	4.09	2.21
**13210**	LVC	VSV-G	TRAC	2	19.70	1.55				
**13719**	LVC	VSV-G	DNMT3A	3	75.10	5.57				
**13800**	VLP	BaEV-Rless	B2M	2	201.61	5.16	175.29	45.14	8.66	4.08
**13207**	VLP	BaEV-Rless	TRAC	2	107.71	5.19				
**13724**	VLP	BaEV-Rless	DNMT3A	3	197.00	11.25				
**13958**	VLP	BaEV-Rless	DNMT3A	5, 6, S5	194.83	13.03				
**13798**	VLP	VSV-G	B2M	2	123.41	5.45	80.37	52.20	4.25	2.26
**13204**	VLP	VSV-G	TRAC	2	22.30	1.65				
**13723**	VLP	VSV-G	DNMT3A	3	95.41	5.66				

Table S2: Raw p-value and adjusted p-value of the paired t-test performed during the rhAmpSeq analysis for editing of TRAC locus by VLPs or electroporated RNPs

TargetID	Target Category	Raw P-value	Adjusted P-value
**chr14-22547516-22547538**	On-target	0.0182	
**chr1-151384698-151384720**	Off-target	0.0007	0.0045
**chr6-36913239-36913261**	Off-target	0.0007	0.0045
**chr12-96190198-96190221**	Off-target	0.0010	0.0045
**chr8-132875597-132875619**	Off-target	0.0046	0.0160
**chr17-63987440-63987462**	Off-target	0.0070	0.0196
**chr19-4716193-4716215**	Off-target	0.0107	0.0249
**chr15-79537361-79537383**	Off-target	0.0778	0.1557
**chr2-162590735-162590757**	Off-target	0.2004	0.3508
**chr7-142536103-142536124**	Off-target	0.3501	0.5331
**chr3-183543358-183543380**	Off-target	0.3808	0.5331
**chr1-11732109-11732131**	Off-target	0.4217	0.5367
**chr10-126993459-126993480**	Off-target	0.4738	0.5527
**chr17-78351754-78351776**	Off-target	0.6446	0.6942
**chr18-57896863-57896885**	Off-target	0.8115	0.8115

Table S3: Raw p-value and adjusted p-value of the paired t-test performed during the rhAmpSeq analysis for editing of LAG3 locus by VLPs or electroporated RNPs

TargetID	Target Category	Raw P-value	Adjusted P-value
chr12-6773277-6773299	On-target	0.2454	
chr2-231766905-231766927	Off-target	0.0011	0.0686
chr16-75111805-75111827	Off-target	0.0032	0.0983
chr7-101283212-101283234	Off-target	0.0116	0.2399
chr14-90593845-90593867	Off-target	0.0223	0.3418
chr1-43279799-43279821	Off-target	0.0298	0.3418
chr2-151203106-151203128	Off-target	0.0395	0.3418
chr1-64133481-64133503	Off-target	0.0422	0.3418
chr15-33180834-33180856	Off-target	0.0486	0.3418
chr22-47924626-47924648	Off-target	0.0551	0.3418
chr3-67021411-67021433	Off-target	0.0551	0.3418
chr16-59634988-59635009	Off-target	0.0617	0.3476
chr1-16396167-16396190	Off-target	0.0717	0.3614
chr12-80031812-80031833	Off-target	0.0758	0.3614
chr5-149864108-149864130	Off-target	0.1202	0.5323
chr1-185656958-185656980	Off-target	0.1828	0.7554
chr3-171739148-171739170	Off-target	0.1959	0.7591
chr8-55778167-55778188	Off-target	0.2535	0.8982
chr20-44423481-44423503	Off-target	0.2714	0.8982
chr1-92332842-92332864	Off-target	0.2945	0.8982
chr12-132325669-132325691	Off-target	0.3109	0.8982
chr2-15796336-15796358	Off-target	0.3144	0.8982
chr18-77403691-77403713	Off-target	0.3357	0.8982
chr7-4961803-4961825	Off-target	0.3468	0.8982
chr2-199457709-199457731	Off-target	0.3516	0.8982
chr12-48796316-48796338	Off-target	0.3636	0.8982
chr16-84248720-84248742	Off-target	0.4103	0.8982
chr2-3558494-3558516	Off-target	0.4243	0.8982
chr10-23362362-23362384	Off-target	0.4269	0.8982
chr2-54513086-54513108	Off-target	0.4633	0.8982
chr10-24231883-24231904	Off-target	0.4634	0.8982
chr14-64741775-64741798	Off-target	0.4726	0.8982
chr12-113056076-113056097	Off-target	0.4966	0.8982
chr10-78507420-78507442	Off-target	0.5031	0.8982
chr7-50764043-50764065	Off-target	0.5056	0.8982
chr3-42907095-42907117	Off-target	0.5070	0.8982
chr17-78952923-78952945	Off-target	0.5323	0.9068
chr19-41001140-41001162	Off-target	0.5537	0.9068
chr15-69319848-69319870	Off-target	0.5631	0.9068
chr3-42876770-42876792	Off-target	0.5897	0.9068

Tables S3 continued

TargetID	Target Category	Raw P-value	Adjusted P-value
chr5-86093860-86093882	Off-target	0.6139	0.9068
chr2-20594328-20594350	Off-target	0.6168	0.9068
chr5-32845893-32845914	Off-target	0.6224	0.9068
chr1-30359564-30359586	Off-target	0.6289	0.9068
chr11-87771025-87771047	Off-target	0.6512	0.9176
chr11-61468178-61468200	Off-target	0.6852	0.9434
chr9-131644512-131644534	Off-target	0.7000	0.9434
chr11-118623457-118623479	Off-target	0.7570	0.9816
chr4-7743129-7743151	Off-target	0.7776	0.9816
chr3-184248398-184248420	Off-target	0.8039	0.9816
chr6-19186689-19186711	Off-target	0.8102	0.9816
chr5-173295132-173295154	Off-target	0.8358	0.9816
chr3-145236856-145236878	Off-target	0.8519	0.9816
chr1-974253-974275	Off-target	0.8539	0.9816
chr1-77338616-77338638	Off-target	0.8701	0.9816
chrX-153921114-153921136	Off-target	0.8708	0.9816
chr1-51549744-51549766	Off-target	0.8921	0.9876
chr6-23272245-23272267	Off-target	0.9107	0.9905
chr8-102730075-102730097	Off-target	0.9266	0.9905
chr19-10287383-10287405	Off-target	0.9612	0.9936
chr6-1267760-1267782	Off-target	0.9831	0.9936
chr17-46865518-46865539	Off-target	0.9834	0.9936
chr14-87161102-87161123	Off-target	0.9936	0.9936

Table S4: Raw p-value and adjusted p-value of the paired t-test performed during the rhAmpSeq analysis for editing of CTLA-4 locus by VLPs or electroporated RNPs

TargetID	Target Category	Raw P-value	Adjusted P-value
**chr2-203870828-203870850**	On-target	0.0020	
**chr2-230823753-230823775**	Off-target	0.0005	0.0089
**chr5-101611404-101611426**	Off-target	0.0023	0.0177
**chr9-135268714-135268736**	Off-target	0.0028	0.0177
**chr11-56459677-56459699**	Off-target	0.0038	0.0181
**chr9-121888845-121888867**	Off-target	0.0080	0.0255
**chr3-4599254-4599276**	Off-target	0.0101	0.0255
**chr12-130006269-130006291**	Off-target	0.0105	0.0255
**chr7-48292864-48292886**	Off-target	0.0107	0.0255
**chr8-142796341-142796363**	Off-target	0.0373	0.0787
**chr17-35769854-35769876**	Off-target	0.0421	0.0799
**chr2-174750411-174750434**	Off-target	0.0811	0.1401
**chr9-36900766-36900787**	Off-target	0.0946	0.1457
**chr4-87900756-87900778**	Off-target	0.0997	0.1457
**chr3-46988547-46988568**	Off-target	0.1305	0.1771
**chr16-31839540-31839562**	Off-target	0.5753	0.7287
**chr14-79111160-79111182**	Off-target	0.6177	0.7335
**chr15-31197425-31197447**	Off-target	0.8981	0.9538
**chr6-151072964-151072986**	Off-target	0.9036	0.9538
**chr20-25328487-25328509**	Off-target	0.9779	0.9779

Table S5: Sequences of the primers used to generate the gRNA-expressing plasmid and for Targeted Deep Sequencing amplicons

Target	gRNA	Protospacer	Sequence-specific primer for mutagenesis	Targeted Deep Sequencing FOR primer	Targeted Deep Sequencing REV primer
**TRAC**	g2	GCTGGTACACGGCAGGGTCA	-	TCCCAGTCCATCACGAGCAGCTGGT	TTGCTCCAGGCCACAGCACTGTTGC
**TRAC**	g3	CAGGGTTCTGGATATCTGT	ACAGATATCCAGAACCCTGCGGTGTTTCGTCCTTTCC	TCCCAGTCCATCACGAGCAGCTGGT	TTGCTCCAGGCCACAGCACTGTTGC
**B2M**	g1	GAGTAGCGCGAGCACAGCTA	TAGCTGTGCTCGCGCTACTCGGTGTTTCGTCCTTTCC	TGGCTTGGAGACAGGTGACGGT	GGAGAACTTGGAGAAGGGAAGTCACG
**DNTM3A**	g2	CCTGCATGATGCGCGGCCCA	TGGGCCGCGCATCATGCAGGCGGTGTTTCGTCCTTTCC	TCCCGATGACCCTGTCTTCCCGTGC	AGGTAGAAGCCATTAGTGAGCTGGC
**DNTM3A**	g6	GCACTGCAAAACGAGCTCAG	CTGAGCTCGTTTTGCAGTGCGGTGTTTCGTCCTTTCC	GGTCTGGACTCCTCTTTGCATCGGGT	GCGAGGTGGAGAGAAAGGAGTCGCT
**LAG3**	g9	GAAGGCTGAGATCCTGGAGG	-	CCTGCACCCTGTTTCTCCCTCGGGA	GAGGGAGTGACACCTCAGGGCAGGG
**CTLA-4**	g9	GGACTGAGGGCCATGGACAC	-	GCTTCGGCAGGCTGACAGC	GCATAGAAGACTGCAATGCAACAGGT

Table S6: Antibodies used in the study

Target	Dye	Clone	Manufacturer	Cat#
**Biotin-APC**	APC	REA746	Miltenyi Biotec	130-110-952
**Cas9**	-	Polyclonal	Takara	632607
**CCR7**	FITC	REA546	Miltenyi Biotec	130-120-468
**CD19-biotin**	-	-	Miltenyi Biotec	130-129-550
**CD25**	PE	4E3	Miltenyi Biotec	130-113-282
**CD39**	BV605	A1	Biolegend	328236
**CD4**	BV785	SK3	Biolegend	344642
**CD4**	APC-Cy7	RPA-T4	BD Bioscience	561841
**CD45RO**	BV650	UCHL1	Biolegend	304232
**CD69**	APC-Cy7	FN50	Biolegend	310914
**CD8**	BV510	SK1	BD Bioscience	563919
**DNMT3A**	-	Polyclonal	GeneTex	GTX129125
**G4S Linker**	PE	E7O2V	Cell Signaling Technology	71645
**GAPDH**	-	0411	Santa Cruz	sc-47724
**HIV-1 Gag p24**	-	749122	R&D Systems Inc	MAB73602
**PD1**	PerCP5.5	EH12.2H7	Biolegend	329914
**TCR α/β**	APC	IP26	Biolegend	306718
**TIM3**	PE-Cy7	F38-2E2	Biolegend	345014
**β2-microglobulin**	PE-Cy™7	2M2	Biolegend	316318
